# Pathogenic bacteria remodel central metabolic enzyme to build a cyclopropanol warhead

**DOI:** 10.1038/s41557-022-01005-z

**Published:** 2022-07-29

**Authors:** Felix Trottmann, Keishi Ishida, Mie Ishida-Ito, Hajo Kries, Michael Groll, Christian Hertweck

**Affiliations:** 1 Department of Biomolecular Chemistry, Leibniz Institute for Natural Product Research and Infection Biology – Hans Knöll Institute (Leibniz-HKI), Jena, Germany; 2 Junior Research Group Biosynthetic Design of Natural Products, Leibniz Institute for Natural Product Research and Infection Biology – Hans Knöll Institute (Leibniz-HKI), Jena, Germany; 3 Center for Protein Assemblies, Chemistry Department, Technical University Munich, Garching, Germany; 4 Faculty of Biological Sciences, Friedrich Schiller University Jena, Jena, Germany

**Keywords:** Biosynthesis, Enzymes

## Abstract

Bacteria of the *Burkholderia pseudomallei* (BP) group pose a global health threat, causing the infectious diseases melioidosis, a common cause of pneumonia and sepsis, and glanders, a contagious zoonosis. A trait of BP bacteria is a conserved gene cluster coding for the biosynthesis of polyketides (malleicyprols) with a reactive cyclopropanol unit that is critical for virulence. Enzymes building this warhead represent ideal targets for antivirulence strategies but the biochemical basis of cyclopropanol formation is unknown. Here we describe the formation of the malleicyprol warhead. We show that BurG, an unusual NAD^+^-dependent member of the ketol-acid reductoisomerase family, constructs the strained cyclopropanol ring. Biochemical assays and a suite of eight crystal structures of native and mutated BurG with bound analogues and inhibitors provide snapshots of each step of the complex reaction mechanism, involving a concealed oxidoreduction and a C–S bond cleavage. Our findings illustrate a remarkable case of neofunctionalisation, where a biocatalyst from central metabolism has been evolutionarily repurposed for warhead production in pathogens.

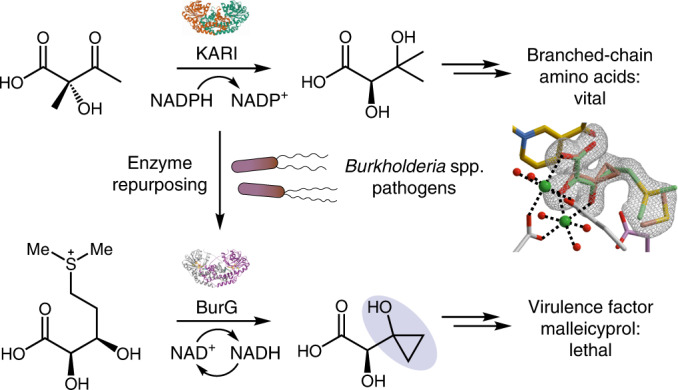

## Abstract (web summary)

*Burkholderia pseudomallei* is a species of bacteria that poses a global health threat and, more generally, bacteria of the *Burkholderia pseudomallei* group cause severe diseases that are recalcitrant to treatment with antibiotics. Now, it has been shown how these infamous pathogens repurpose the widespread enzyme BurG to produce a reactive cyclopropanol head group found in the virulence-promoting malleicyprol toxins. Interrupting the synthesis of the cyclopropanol warhead is a potential route for developing antivirulence treatments.

## Main

**Fig. 1:**
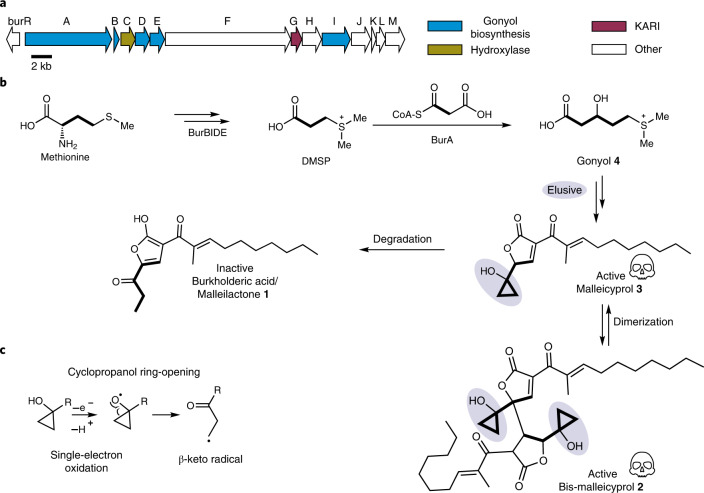
Malleicyprols, virulence factors encoded by conserved locus in BP group pathogens. **a**, Gene cluster coding for malleicyprol biosynthesis. KARI, ketol-acid reductoisomerase **b**, Structures of malleicyprols **2** and **3** and their degradation product **1**, and selected steps in the biosynthesis of the C5 building block. See Extended Data Fig. 1. for detailed biosynthetic pathway. **c**, Ring-opening of cyclopropanols mediated by single-electron oxidation.

**Extended Data Fig. 1:**
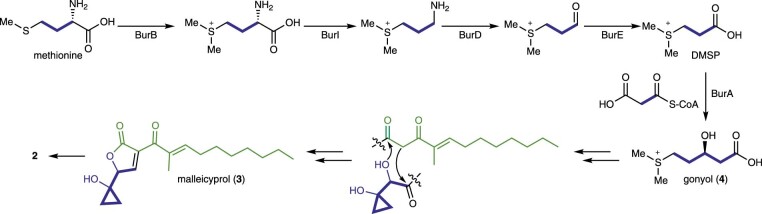
Model of biosynthetic pathway to malleicyprol. More detailed model of the biosynthetic pathway to malleicyprol.

A holistic view on the molecular mechanisms underlying *Burkholderia pseudomallei* (BP)-mediated diseases is urgently needed for the development of therapies, including vaccination and antivirulence strategies. Beyond typical disease-promoting factors^1^ such as secretion systems, capsular polysaccharides, a protein toxin^2^ and siderophores^3–5^, recent studies indicate that the role of the specialized secondary metabolome of BP bacteria has been greatly underestimated^6,7^. All members of the BP complex, including the closely related, yet less virulent model organism *Burkholderia thailandensis*, harbour a highly conserved gene locus (*bur*/*mal*, Fig. 1a) coding for an unusual polyketide synthase^8,9^. According to animal^10,11^ and cell-based assays using targeted mutants^8,12^, this gene cluster is critical for virulence. The isolated metabolite (burkholderic acid/malleilactone **1**; Fig. 1b) initially assigned as product of the assembly line, however, could not explain the observed phenotypes^8,9^. Reinvestigation of the metabolome revealed previously overlooked cyclopropanol-substituted congeners named malleicyprols (bis-malleicyprol **2** and malleicyprol **3**; Fig. 1b), which show high toxicity in cell assays and in a nematode model^13^. The effect of malleicyprols is attributed to a cyclopropanol warhead that is known to readily form β-keto radicals upon single-electron oxidation and ring opening (Fig. 1c)^14–16^. Consequently, the previously detected propanone-substituted metabolite **1** represents the inactive form. Whereas mutational analyses, in vitro assays and isotope-labelling experiments indicated that the reactive cyclopropanol derives from methionine via the zwitterionic gonyol **4** (Fig. 1b and Extended Data Fig. 1)^17^, the key biosynthetic steps that install the cyclopropanol warhead have remained obscure.

## Results and discussion

### The elusive cyclopropanol precursor

**Fig. 2:**
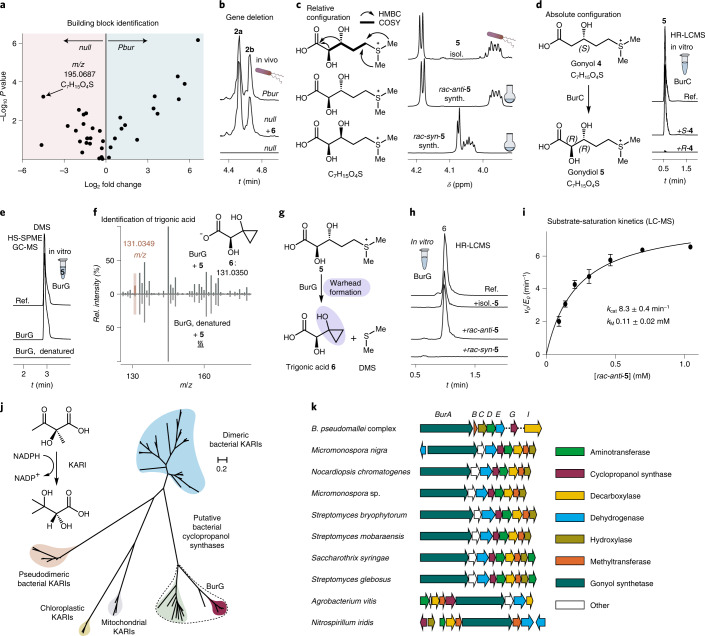
Substrate identification and reconstitution of enzymatic cyclopropanol formation. **a**, Metabolomics analysis of *B. thailandensis Pbur* versus *B. thailandensis Pbur BurG::Kan* (malleicyprol *null* mutant) cell extracts (biological triplicates, *n* = 3). Data filtered for sulfur-containing compounds; see Supplementary Fig. 1 for non-filtered analysis. **b**, Chemical complementation of the *null* mutant with **6** restores malleicyprol production (isoforms **2a** and **2b**) to wild-type level. **c**, Structure elucidation of isolated (isol.) gonydiol **5**, and comparison of key ^1^H NMR shifts with synthetic (synth.) racemic *syn*- and *anti*-isoforms. COSY, correlated spectroscopy; HMBC, heteronuclear multiple bond correlation. **d**, Formation of **5** catalysed by hydroxylase BurC in assays with addition of *R*-**4** or *S*-**4**; top trace: reference (**5**). **e**, Detection of DMS as product of BurG reactions by GC-MS. **f**, Mirror plot of enzymatic assay (top) of BurG incubated with **5** versus denatured BurG (bottom) indicates a compound with the *m*/*z* value of **6** as enzymatic product; see Extended Data Fig. 3 for chromatogram. **g**, BurG-catalysed transformation of **5** to **6**. **h**, Monitoring of enzymatic turnover of isolated **5** and synthetic *rac*-*syn-***5** or *rac*-*anti*-**5** into **6** via HR-LCMS. **i**, Steady-state kinetics of formation of **6** from *rac*-*anti*-**5**; mean and standard deviation obtained from two independent preparations (*n* = 2). **j**, Phylogenetic analysis of BurG, orthologues and canonical KARIs. The scale bar indicates amino acid substitutions per site. **k**, Alignment of biosynthetic gene cluster encoding for BurG orthologues clustered with the genes associated with biosynthesis of **5**. Source data

**Extended Data Fig. 2:**
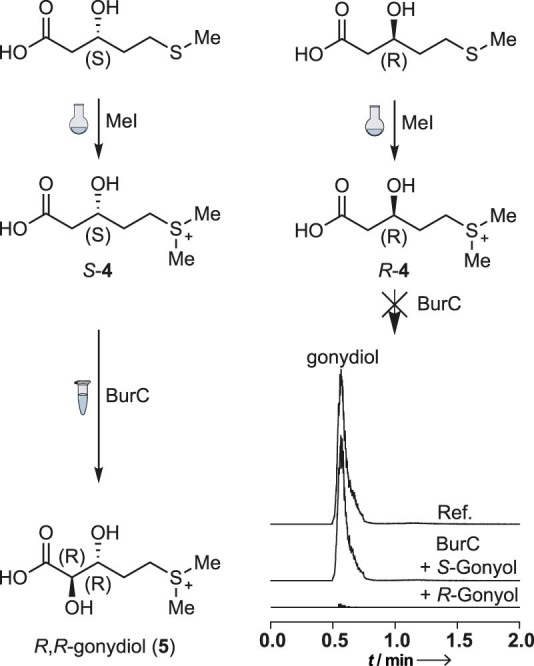
Absolute configuration of gonydiol. Synthesis of *R*- and *S*-gonyol (**4**) and subsequent enzymatic transformation to gonydiol (**5**) establishes the absolute configuration of naturally occurring gonyol and gonydiol. HR-LCMS extracted ion monitoring of gonydiol (*m*/*z* of 195.0686 in positive-ion mode) after addition of *R*-**4** or *S*-**4** to reaction mixtures containing the hydroxylase BurC. Source data

Elucidating the biochemical basis of cyclopropanol formation was challenging because no other pathways to cyclopropanol-bearing natural products^18^ were known and the *bur/mal* gene clusters did not point to plausible biocatalysts. Hence both the substrate and the timing of the reaction were unclear. Therefore, we first sought to elucidate the steps downstream of gonyol formation by systematically inactivating uncharacterized genes of the *bur*/*mal* cluster in the engineered malleicyprol overproducer strain *B. thailandensis Pbur*^9^. Monitoring metabolic changes by comparative liquid chromatography–high-resolution mass spectrometry (LC-HRMS) analyses we noted that a mutant lacking *burG,* in lieu of malleicyprols, produces a new compound (**5**) with an *m*/*z* of 195.0687 (M + H^+^; Fig. 2a and Supplementary Fig. 1) and a deduced chemical formula of C_7_H_15_O_4_S (Fig. 2b). Its polarity and molecular composition suggested that **5** represents a biosynthetic intermediate that could arise from hydroxylation of gonyol (C_7_H_15_O_3_S). Isolation of **5** for a full structure elucidation proved to be challenging due to its high polarity. Eventually, using an optimized purification protocol, we obtained **5** as its trifluoroacetate (TFA) salt in sufficient amount (1.3 mg l^−1^) and purity to unequivocally elucidate its structure. NMR data suggested that **5** is a sulfonium-substituted carboxylic acid with two hydroxy substituents at the α and β positions (Fig. 2c). We confirmed the structure of **5** (named gonydiol) and determined the *anti*-configuration of the hydroxy groups by chemical synthesis (Supplementary Fig. 2) of *anti*- and *syn*-configured reference compounds and NMR comparisons (Fig. 2c). We reasoned that **5** could result from hydroxylation of **4** by the gene product of *burC*, a putative α-ketoglutarate-dependent dioxygenase. To reconstitute the biotransformation, we cloned *burC*, heterologously produced His_6_-tagged BurC in *Escherichia coli*, and synthesized enantiomerically pure *R*- and *S***-4** as potential substrates (Extended Data Fig. 2). In the in vitro assays BurC specifically transforms *S*-gonyol into *anti*-gonydiol (Fig. 2d). Consequently, the absolute configuration of natural gonydiol, a precursor of the cyclopropanol-bearing building block, is 2*R*,3*R*.

### Discovery of a cyclopropanol synthase

Accumulation of **5** in the *burG*::*Kan* mutant strongly suggested that this metabolite is the substrate for BurG downstream of the pathway towards malleicyprol. A sequence similarity search using BLAST predicted that BurG belongs to the family of ketol-acid reductoisomerases (KARIs; EC 1.1.1.86), well-characterized enzymes that play key roles in the biosynthesis of branched-chain amino acids^19^. To study the function of BurG, we heterologously produced a His_6_-tagged variant in *E. coli* and incubated purified His_6_-BurG with isolated and synthetic substrates. We noted the characteristic smell of dimethylsulfide (DMS) when opening closed reaction tubes of assays containing natural or the synthetic *anti*-**5**. In contrast, DMS was not detectable in assays containing either the *syn*-isomer or heat-denatured BurG. We corroborated these findings by solid-phase microextraction–gas chromatography–mass spectrometry (SPME–GC–MS) gas-phase analysis of the respective assays (Fig. 2e).

**Extended Data Fig. 3:**
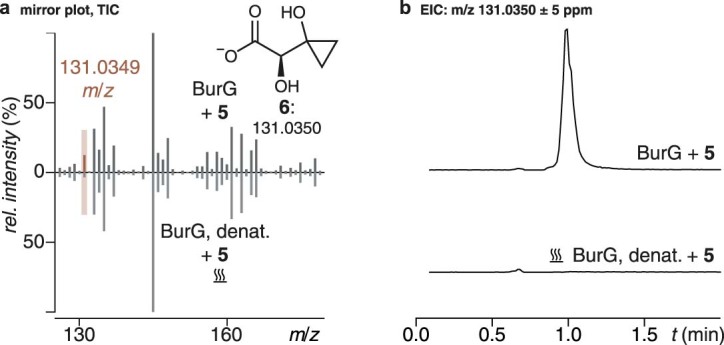
Identification of trigonic acid (6). **a**, Mirror plot of enzymatic assay (top) of BurG incubated with **5** vs. denatured BurG (bottom) indicates a compound with the *m*/*z* value of **6** as enzymatic product. **b**, Extracted ion chromatogram (EIC) in negative ion mode of the enzymatic assay (top) vs. denatured BurG (bottom). Source data

**Extended Data Fig. 4:**
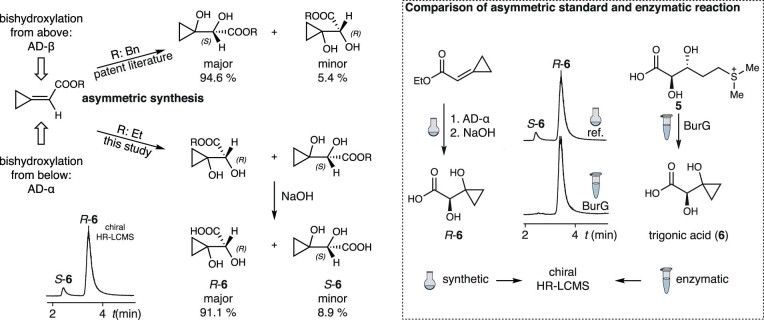
Determination of stereochemistry of trigonic acid (6). Left panel: stereoselectivity of the asymmetric dihydroxylation (AD)-mix β leads to mainly *S*-configurated products^20^; Bn: benzyl protecting group. Consequently AD-mix α forms mainly *R*-configurated products. Chiral HR-LCMS (bottom left) validates the successful asymmetric synthesis with AD-mix α after hydrolysis of the formed ethyl ester (Et). Right panel: chiral HR-LCMS of asymmetric synthesis products (ref.) vs. enzymatically formed **6** shows the stereochemistry of enzymatically formed **6** to be of *R*-configuration. Source data

Consequently, BurG catalyses a specific C–S bond cleavage reaction to form DMS and a second reaction product, probably composed of the remaining five carbon atoms of **5**. By comparison of the averaged mass spectra of total ion currents we identified a compound (**6**, named trigonic acid) with *m*/*z* 131.0349 that is only present in the positive assays with intact BurG (Fig. 2f and Extended Data Fig. 3). The deduced chemical formula of C_5_H_8_O_4_ for the uncharged species of **6** is in agreement with a compound resulting from the elimination of DMS from **5** (C_7_H_15_O_4_S [M + H]^+^) and indicated an additional ring double-bond equivalent compared to **5**. We thus anticipated that BurG catalyses a cyclization reaction, tentatively yielding a cyclopropanol residue as in **6** (Fig. 2g). To prove the proposed structure of **6** we synthesized *rac*-**6** and confirmed its identity with the enzymatically formed product by HR-LCMS (Fig. 2h; see Supplementary Fig. 3 for tandem MS). Moreover, complementation of *B. thailandensis Pbur burG::Kan* cultures with synthetic *rac-***6** fully restored malleicyprol production (Fig. 2b). To elucidate the absolute configuration of **6**, we synthesized enantiomerically enriched *R*-**6** (e.e. 82%) by asymmetric Sharpless bishydroxylation^20,21^ and proved its identity with the product of the enzyme assay by chiral HR-LCMS analysis (Extended Data Fig. 4). These results not only demonstrate that the cyclopropanol-substituted molecule **6** in its *R*-configuration is a true pathway intermediate but also that BurG catalyses an unusual cyclization reaction yielding the malleicyprol warhead.

### BurG catalyses a transient redox reaction

The formation of a strained cyclopropane ring markedly differs from the biotransformations known for canonical KARIs. Notably, the genomes of BP bacteria harbour additional KARI genes (*ilvC*) within the operons for branched-chain amino acid biosynthesis. A phylogenetic analysis (Fig. 2j and Supplementary Fig. 4) of BurG-like enzymes and native KARIs revealed a separate clade for tentative cyclopropanol synthases within the KARI family. We identified nine other organisms (Fig. 2k) that harbour *burG* orthologues along with all genes required for gonydiol biosynthesis.

HHpred analyses^22^ predicted the typical KARI Rossmann fold domains for these BurG-type enzymes, pointing to the binding of a nucleotide cofactor. Indeed, HR-LCMS analysis of a denatured BurG preparation revealed the presence of the nucleotide cofactor NAD^+^ (Supplementary Fig. 5). This finding is surprising because KARIs are known to prefer NADPH as cofactor. In this context it is noteworthy that considerable effort has been directed towards engineering KARIs to utilize NADH instead of NADPH^23^ and to discover rare NADH-preferring KARIs for biotechnological purposes^24^.

**Fig. 3:**
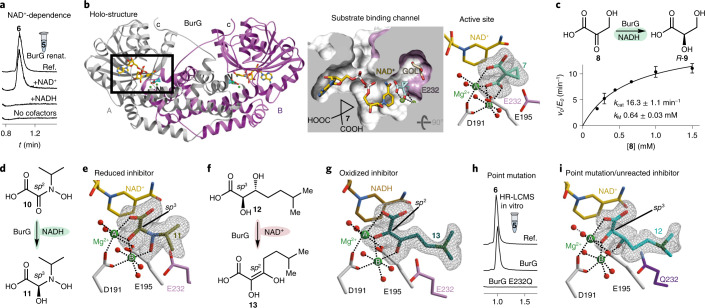
Concealed redox cycle catalysed by BurG. **a**, Gonydiol **5** transformation assay with NAD^+^-free BurG with addition of NAD^+^, NADH or in the absence of cofactor; top: **6** as reference. **b**, Full view of the 3D structure of BurG co-crystallized with the KARI inhibitor **7** (left). A and B, subunits; N and C, termini. The black rectangle displays the area for the zoom in the substrate binding channel (middle) and active site zoom (right) (PDB 7PCG). GOL, glycerol. **c**, Steady-state kinetics of NADH-mediated reduction of hydroxypyruvate **8** to glyceric acid **9** through BurG; mean and standard deviation as obtained from two independent protein preparations (*n* = 2). **d**, Scheme of the transformation of hydroxamate **10** to hemiaminal **11**. **e**, 3D structure of BurG co-crystallized with **11** after incubation with **10** and NADH (PDB 7PCL). Two magnesium atoms (A and B) are shown as green spheres. **f**, BurG-mediated oxidation of carba-gonydiol **12** to the enolate **13**. **g**, 3D structure of BurG with **13** obtained after co-crystallization with **12** and NAD^+^ (PDB 7PCM). **h**, Single amino acid substitution (E232Q) renders BurG inactive. **i**, 3D structure of BurG E232Q co-crystallized with **12** (PDB 7PCO). Source data

To test whether NAD^+^ is essential for the BurG-mediated cyclopropanol formation we removed the cofactor from the enzyme preparation by using an extensive dialysis protocol. In the absence of NAD^+^, BurG is incapable of forming **6**, yet the activity can be restored by addition of NAD^+^. Addition of NADH, however, cannot restore the reaction (Fig. 3a). These results provide evidence that bound NAD^+^ is essential for the BurG-catalysed reaction.

**Extended Data Fig. 5:**
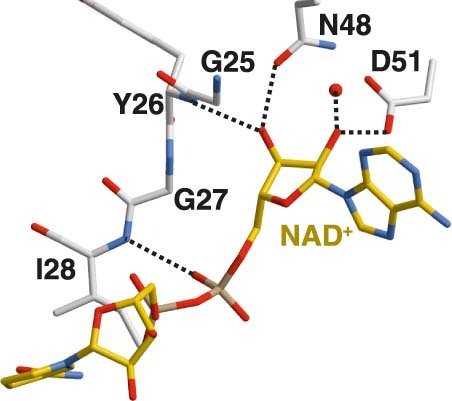
Binding mode of NAD^+^ in the active site of BurG. The amino acid residues Asn48 and Asp51 exclude the negatively charged phosphate group of NADP^+^ (as commonly found in canonical KARIs) from binding in the active centre of BurG.

To gain more insight into the reaction mechanism and the role and position of NAD^+^ in the enzyme, we performed protein crystallography. Due to the close phylogenetic relation between BurG and the KARIs, we turned to surrogates that had proven successful in previous studies in obtaining ligand-bound three-dimensional (3D) structures such as the KARI inhibitor cyclopropane-1,1-dicarboxylate **7** (ref. ^19^). We raised crystals of holo-BurG in the absence or presence of **7** as surrogate and solved the structures at a resolution of 1.8 Å and 1.9 Å, respectively. The obtained 3D structure of the complex (Fig. 3b; PDB 7PCG) clearly displays **7** bound between two magnesium ions with NAD^+^ in close proximity, thus enabling its participation in biocatalysis (Fig. 3b). The amino acid residues Asn48 and Asp51 exclude the negatively charged phosphate group of NADP^+^ from binding in the active centre of BurG (Extended Data Fig. 5). A co-crystallized glycerol molecule (used as cryoprotectant) maps the entire specificity pocket next to the catalytic centre. The positioning of NAD^+^ in the active site of BurG indicates that an oxidation event occurs during cyclopropanol formation. Monitoring the BurG assays by HR-LCMS, however, did not show the presence of any oxidized derivatives of **5** or **6** (or any other pathway intermediates). Therefore, BurG is either not redox proficient or a transient redox step occurs during its catalytic cycle. Such a cryptic (or covert) redox reaction would first form NADH by substrate oxidation, followed by NAD^+^ regeneration through ligand reduction.

To investigate the redox proficiency of BurG and its ability to regenerate NAD^+^ from NADH we devised an absorbance-based assay with the substrate surrogate hydroxypyruvate **8**. BurG was incubated with **8** and NADH and the light absorption of the dinucleotide at 340 nm was monitored spectrophotometrically. A clearly observed signal decrease corresponds to the formation of NAD^+^ and glyceric acid **9**, the reduced form of **8** (Fig. 3c). In substrate saturation kinetics we determined a *k*_cat_ of 16.3 ± 1.1 min^−1^ (Fig. 3c and Supplementary Fig. 6) for this reaction, which resembles the *k*_cat_ for ketone reduction by other KARIs^19^. Further evidence for the redox proficiency of BurG was gleaned from protein crystallization experiments with the previously co-crystallized^19^ KARI inhibitor *N*-hydroxy-*N*-isopropyloxamic acid (IpOHA, **10**) in the presence of NADH (Fig. 3d; PDB 7PCL). The 3D structure at 2.05 Å resolution unexpectedly shows that the hydroxamate **10** was reduced to the corresponding hemiaminal **11**, as evidenced by the tetrahedral geometry of the surrogate in the 3D structure (Fig. 3e). Notably, the stereochemical course of the reduction is identical with the native product of BurG. Taken together, BurG is a redox-proficient enzyme that promotes cyclopropanol formation via a transient and thus concealed oxidation event. This finding is surprising because characterized enzymes that recycle their NAD^+^ cofactors are scarce^25–27^.

**Extended Data Fig. 6:**
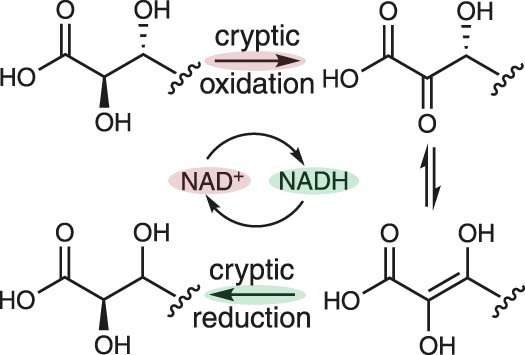
Cryptic redox cycle. Scheme representing the general concept of a cryptic redox cycle.

**Extended Data Fig. 7:**
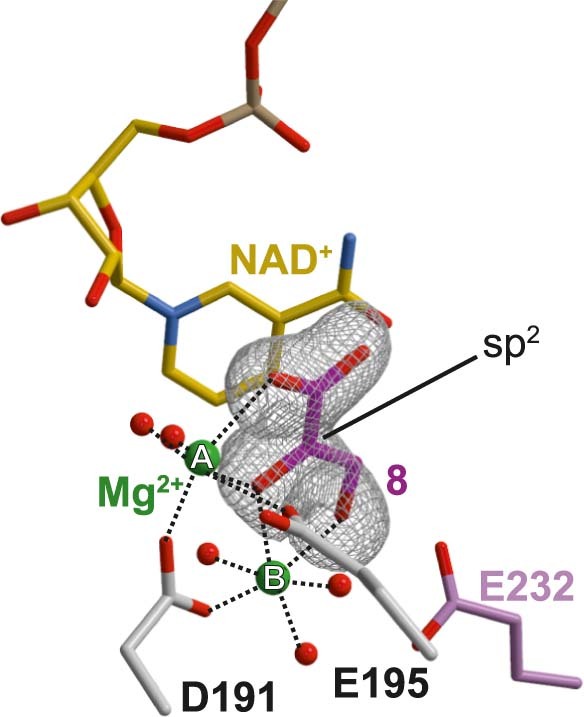
Complex structure of BurG co-crystallized with hydroxypyruvate (8) (PDB ID: 7PCI). The F_O_-F_C_ electron density map (contoured to 3 σ) for **8** bound to the Mg-atoms is shown as grey mesh. The ligand has been omitted for phasing. E232 is located on subunit B and marked in pink.

To provide further experimental evidence for the proposed transient redox cycle (Extended Data Fig. 6) we captured the enzyme in a state with bound NADH after the oxidation event occurred. Therefore, we synthesized the carba analogue of gonydiol, *rac*-**12**, which cannot be converted into a cyclopropanol (Fig. 3f), and performed protein crystallography. Analysis of the obtained 3D structure with a resolution of 2.05 Å of BurG with *rac*-**12** revealed that the surrogate had been converted into the oxidized form **13** that is complexed between the two magnesium atoms and NADH in the active site of the enzyme (Fig. 3g; PDB 7PCM). The planar geometry of the complexed ligand indicates an *sp*^2^ hybridization of both the α and β carbons, indicating that **13** is stabilized in the enolic form. The transformation of *rac*-**12** into the trapped enolate **13** provides evidence for an intermediary oxidation during the catalytic cycle of BurG. We obtained similar results in co-crystallization assays with BurG, NAD^+^ and the substrate surrogate hydroxypyruvate **8**. By analogy to **13**, the crystal structure depicts a planar geometry of **8** indicating it is bound in its enolized form to the active site of BurG (Extended Data Fig. 7; PDB 7PCI).

### Mechanism of cyclopropanol formation

Our structural analysis revealed that the carboxylate of a glutamic acid side chain (E232) is in close proximity (3 Å) to the β carbons of the substrate surrogates and thus probably facilitates the deprotonation event necessary for enol formation. To probe the function of this putative catalytic residue we generated a point-mutated variant (E232Q) of BurG and assayed its activity with the native substrate gonydiol. As predicted, the mutation hampers the transformation of gonydiol into the cyclopropanol derivative **6** (Fig. 3h). The redox proficiency of BurG E232Q remained intact as indicated by a *k*_cat_ of 8.2 ± 0.3 min^−1^ for the NADH-mediated reduction of **8** (Supplementary Fig. 7). Co-incubations of BurG E232Q with *rac*-**12** yielded crystals for which we solved a 3D structure at a resolution of 1.55 Å. In contrast to native BurG, *rac*-**12** was not oxidized by BurG E232Q, as indicated by the tetrahedral geometry of the complexed surrogate (Fig. 3i; PDB 7PCO). The absence of oxidized **13** in BurG E232Q may indicate a role of E232 in shifting the internal redox equilibrium towards the oxidized state by deprotonating the substrate and forming the enolate. By analogy to glycerate oxidation, the oxidized state is probably energetically less favoured.^28^ The obtained near-atomic resolution structure with BurG E232Q and **12** enabled us to analyse the bound ligand and determine its absolute configuration (2*R*,3*R*). The observed chirality of the bound substrate confirmed our stereochemical analysis that gonydiol had the 2*R*,3*R* configuration.

**Fig. 4:**
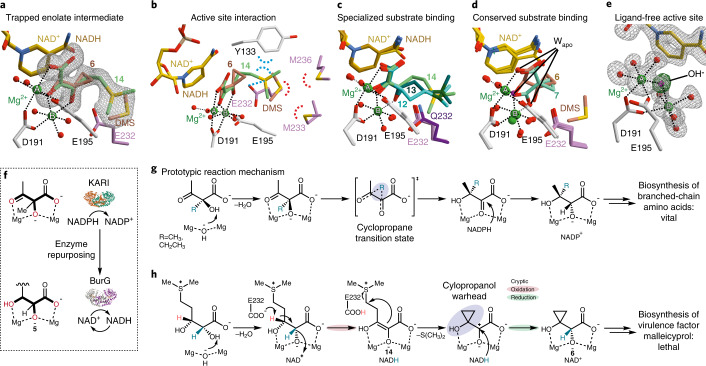
Parallels and differences between biocatalysis of BurG and canonical KARIs. **a**, Structures of BurG with the reaction products **6** and DMS and the trapped enolate intermediate **14** after co-crystallization with gonydiol **5** (PDB 7PCN). Two magnesium atoms (A and B) are shown as green spheres. **b**, Interaction of active site residues of BurG with **6**, **14** and DMS. **c**, Structural overlay of BurG bound to **12**, **13** and **14** indicates a strong interaction of the positively charged sulfonium moiety of **14** with E232 as compared to neutral **12** and **13**. **d**, Tight lock of the oxygen-functionalized compounds **6** and **7** through interaction with Mg^2+^ ions in the active centre of BurG. W_apo_ indicates the binding sites of water molecules as found in the apo structure that are replaced by ligand atoms in the depicted structures. **e**, Structure of unliganded holo-BurG illustrates a hydroxide ion bridged between two Mg^2+^ centres (PDB 7PCC). **f**, Comparison of BurG and KARIs displays a similar binding mode of substrates in the Mg^2+^ cluster. **g**, Proposed reaction mechanism^19^ for KARIs with cyclopropane transition state. **h**, Reaction mechanism proposed for BurG involving a concealed (transient) redox reaction.

**Extended Data Fig. 8:**
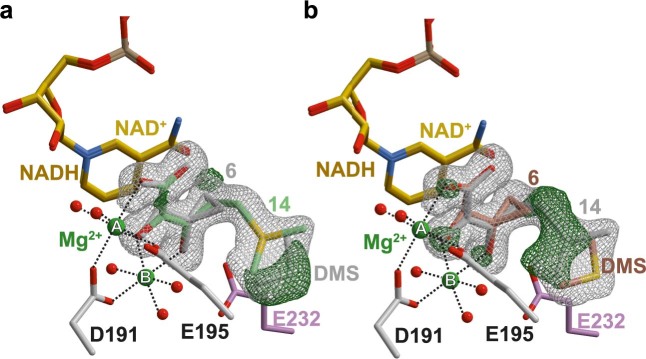
Complex structure of BurG co-crystallized with gonydiol (5) (PDB ID: 7PCN). The 2F_O_-F_C_ electron density maps (grey meshes, contoured to 1.0 σ) and the F_O_-F_C_ maps (green meshes, contoured to 3.0 σ) are depicted for the respective ligand. **a. 6** and DMS (shadowed in grey) have been omitted for phasing. **b. 14** (shadowed in grey) has been omitted for phasing. The F_O_-F_C_ electron density maps clearly depict the excluded ligands, proving that **14**, **6** and DMS are all bound to the active site. The occupancy of each ligand was set to 0.5. E232 is located on subunit B and marked in pink.

Apart from its function as a catalytic base, E232 also plays an active role in substrate recognition and positioning as we gleaned from crystallization assays with gonydiol and BurG. We solved a 3D structure (at 1.6 Å) for which the electron density map shows a mixed population of two subsets of the converted substrate (Fig. 4a and Extended Data Fig. 8; PDB 7PCN). In one subset the active centre is populated by the enzymatic reaction products **6** and DMS. The second subset clearly depicts the backbone of gonydiol, which was oxidized into its enolate **14**. Intermediate **14** is firmly held in place by ionic interaction of its sulfonium moiety with E232 and cation–*π* interaction with Y133 (Fig. 4b). In contrast, the neutral carba analogues **12** and **13** show a higher degree of freedom in their tail regions (Fig. 4c) due to the lack of ionic placement as visible in their crystallographic B factor.

The head region (C1–C3) of all co-crystallized ligands was consistently found to be firmly locked in a conserved binding mode that is facilitated by interactions with the two Mg^2+^ ions of the enzyme (see Fig. 4d,e for unliganded holo structure). In this head region, BurG shows remarkable similarity with regards to substrate structure and binding when compared to canonical KARIs (Fig. 4f). The 3D structure obtained for the holo version of BurG (PDB 7PCC) illustrates that the binding sites of the Mg-bound oxygen functionalities are populated by aqueous residues when no ligand is present (Fig. 4e). In addition to similarities in substrate binding and redox capability, canonical KARIs are thought to contain a cyclopropane transition state^19^ in their catalytic cascade (Fig. 4g). In the case of BurG, formation of **6** is probably driven by a gain in entropy and removal of the volatile DMS from the chemical equilibrium after cyclopropanol synthesis. Moreover, the enzyme surrounds the cationic sulfonium group of the substrate with a hydrophobic binding pocket lined with Met, Tyr and Val residues, which favour loss of the charge and thus promote catalysis (Fig. 4b).

**Extended Data Fig. 9:**
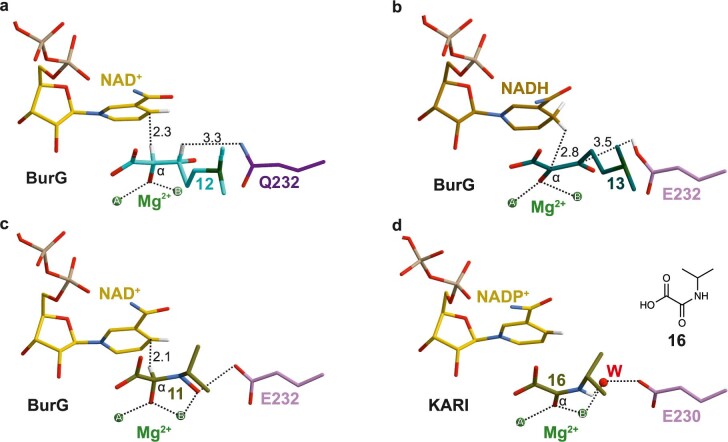
Cofactor and ligand orientation in the active site of BurG and KARIs. The structural comparison between BurG and KARIs illustrates the preferred reaction trajectories of hydride transfer in the distinct enzymes. The interactions of the ligand with the two Mg^2+^ ions (black dots) indicate that C_α_ is the preferred carbon atom for catalysis. **a**, Structure of the catalytically inactive BurG E232Q mutant in complex with surrogate **12**. The ligand adopts ideal orientation for hydride transfer to NAD^+^ (2.3 Å distance, angle between C(NAD^+^)HC_α_: 174°, PDB code: 7PCO). **b**, Structure of BurG co-crystallized with **12** and NAD^+^. The electron density shows the ligand oxidized to **13** resulting in NADH. Glu232 acts as base and is key to hydride transfer. Distance and angle between the cosubstrate and C_α_ (2.8 Å, C(NADH)HC_α_: 123°) are unfavourable for the back reaction. The replacement of sulfur by carbon in surrogate **13** prevents the elimination reaction and keeps the metabolite in its position (PDB code: 7PCM). **c**, Structure of BurG co-crystallized with **10** and NADH. The electron density shows the C_α_-atom of the ligand in sp^3^ configuration. Therefore, the molecule represents the carbinolamine **11** and the cosubstrate is oxidized to NAD^+^. Distance and angle between the cosubstrate and C_α_ allow for a back reaction (2.1 Å, C(NAD^+^)HC_α_: 168°). However, formation of the alkoxide is favoured by the interactions with both Mg^2+^ ions (PDB code: 7PCL). **d**, Active site architecture of the KARI from *Staphylococcus aureus* (PDB code: 6AQJ). Crystallization of the enzyme was carried out in presence of **10** and NADPH. It is reported^19^ that hydride transfer from NADPH to the nitrogen of **10** takes place, hereby forming NADP^+^ and converting the ligand to **16** via water (W) release. The complex structure lacks defined H-bonds between cosubstrate and ligand.

**Extended Data Fig. 10:**
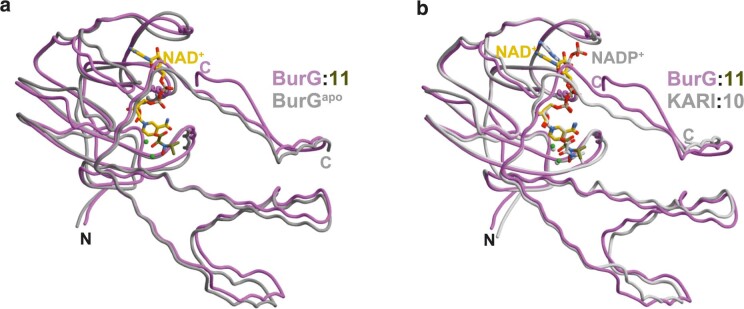
Structural overlays. **a**, Structural overlay of BurG (holo) bound to **11** (PDB code: 7PCL, coloured in pink) with apo BurG (PDB code: 7PCE, coloured in grey). The root mean square deviation (rmsd) between both structures is 1.2 Å over 283 C^α^-atoms (353 residues in total). BurG (apo) displays increased flexibility (high temperature factors) and individual secondary structure elements are shifted compared to BurG (holo). As a result, the catalytic centre in apo BurG is accessible to cofactors and ligands (open conformation). In contrast, the active site of BurG (holo) is sequestered in a closed chamber. **b**, Structural overlay of BurG (holo) (PDB code: 7PCL, coloured in pink) with the ketol-acid reductoisomerase (KARI, holo) from *Staphylococcus aureus* (PDB code: 6AQJ, coloured in grey). The KARI from *S. aureus* is bound to **10** while BurG is bound to **11** (the reduced form of **10)**. Both structures adopt the closed conformation and display a rmsd of 1.4 Å over 288 C^α^-atoms (sequence identity 35 %).

Based on these results, we devised a plausible reaction mechanism for the BurG-mediated cyclopropanol formation (Fig. 4h). First, by analogy to the initial step in the catalytic cycle of canonical KARIs^29^, the hydroxide bridged between the magnesium centres (shown in the ligand-free holo structure of BurG; Fig. 4e), would deprotonate the α-hydroxy moiety of **5**, thus forming the corresponding alkoxide. Next, the two magnesium ions would complex the alkoxide and position it above the pyridinium moiety of the NAD^+^ cofactor. In addition, the glutamate residue (E232) would ionically interact with the positively charged sulfonium group to firmly position the substrate in the binding pocket. Subsequent hydride transfer from the C-α of gonydiol to NAD^+^ (yielding NADH), and deprotonation at the β carbon by E232 would result in an enolate intermediate (**14**), as mimicked by hydroxypyruvate and the oxidized carba-gonydiol analogue **13**. The ideal positioning of C-α relative to the hydride-accepting NAD^+^-carbon (Extended Data Fig. 9) renders an alternative mechanism involving oxidation at C-β unlikely. Quenching of the negative charge at E232 through proton transfer would disrupt the ionic interaction, destabilize the sulfonium and trigger cyclization. Next, an intramolecular nucleophilic attack at the δ position would result in cyclopropyl ring formation and C–S bond cleavage followed by hydride transfer from NADH to the *si* face of the C-α atom. The proposed stereochemical course of hydride transfer is in line with the determined stereochemistry of the final product **6** and the orientation found of all surrogates in the active site of the enzyme (Fig. 4a). This key reductive step would regenerate the NAD^+^ cofactor, leaving the overall redox reaction neutral and retaining the configuration at C2. Our structural analysis indicates that the outlined reaction sequence takes place in a closed conformation of the active site (see Extended Data Fig. 10 for comparison of closed and open/apo conformations). This enzymatic envelope would protect the oxidized and highly reactive intermediates from unwanted side reactions. Hence, final expulsion of the reaction products from the active site would prepare the enzyme for the start of a new catalytic cycle.

## Discussion

We reason that the KARI-fold is ideally positioned as enzymatic ancestor from primary metabolism to be repurposed for gonydiol binding, a transient redox reaction, and cyclopropanol construction by elimination of DMS. The fact that canonical KARIs facilitate their reaction sequence to branched carbon backbones through a cyclopropane transition state hints to the evolutionary trajectory (neofunctionalisation) towards the cyclopropanol synthase BurG. Inheritance of the redox capability of KARIs allows BurG to activate its substrate gonydiol as the electrophilic intermediate **14**. This activation is vital for intramolecular electrophilic displacement of DMS as leaving group and subsequent cyclopropanol formation. Most characterized biosynthetic pathways towards cyclopropyl^30–35^ moieties, as in the genotoxin colibactin^36,37^, for example, also employ electrophilic displacement reactions^38^. Generation of the enolic nucleophile, however, is commonly achieved via a thioester linkage between enzyme and substrate, and in all studied cases either methylthioadenosine or chloride are employed as leaving groups. The KARI-mediated reaction discovered here may inspire chemical engineers who seek to incorporate highly strained rings in a biocatalytic fashion^39^. Our work offers the prospects of finding new cyclopropanol-forming enzymes and the corresponding natural products. More importantly, our mechanistic and structural study of the key enzyme responsible for malleicyprol biosynthesis sets the basis for selectively inhibiting the formation of virulence-conferring toxins from pathogenic *B. pseudomallei* complex bacteria.

## Methods

### Bacterial strains and general culture conditions

The *B. thailandensis* wild-type strain E264 (DSM13276) was obtained from DSMZ. Cultures of *B. thailandensis* E264, *B. thailandensis Pbur* and *B. thailandensis Pbur burG::Kan* were either grown in MM9^9^ liquid medium, LB-liquid or LB-agar medium at 30 °C. Cultures of mutant strains were supplemented with either tetracycline (45 μg ml^−1^, *B. thailandensis Pbur*) or tetracycline (45 μg ml^−1^) in addition to kanamycin (150 μg ml^−1^, *B. thailandensis Pbur burG::Kan*). Liquid cultures were grown in 300 ml (50–75 ml culture volume) and 1 litre (250 ml culture volume) baffled Erlenmeyer flasks with orbital shaking at 30 °C (*B. thailandensis*) or 37 °C (*E. coli*). To obtain biomass for metabolite extraction, the grown cells were pelleted by centrifugation (6,000*g*, 10 min).

### Suicide plasmid preparation for *B. thailandensis* mutant

A gene fragment (2,349 bp) containing *burG* was PCR-amplified using primers Isomerase-fw and Isomerase-rv with 5 Prime Extender Polymerase (VWR International). The amplicon was purified using the GFX PCR DNA and Gel Band Purification Kit (GE Healthcare), and the obtained DNA was cloned into the pGEM T-easy vector (Promega). The resulting plasmid (pGEM-Isomerase) was verified by sequencing (GATC biotech) and restricted with the restriction enzyme *Sma*I and treated with SAP (Shrimp Alkaline Phosphatase, New England Biolabs). pGEM-Kan was restricted by *Not*I and blunted by Klenow treatment. This blunted kanamycin resistance cassette gene was then cloned into the *Sma*I-restricted plasmid, generating the Kan-inserted suicide plasmid pGEM-*burG::Kan*.

### Preparation of gene insertion mutant *B. thailandensis Pbur burG::Kan*

*B. thailandensis E264 Pbur* was precultured in LB medium supplemented with tetracycline (45 μg ml^−1^) for 16 h at 30 °C. The cultured cells were inoculated in LB medium with tetracycline (45 μg ml^−1^) and grown at 30 °C until an OD_600_ of 0.4–0.6 was reached. Subsequently, the culture broth was centrifuged and the supernatant was removed. Precipitated cells were resuspended in sucrose solution (300 mM) and centrifuged. After repeating this washing step three times, the washed cells were resuspended in 300 mM sucrose and subjected to electroporation (200 kV, Eporator, Eppendorf) with pGEM-*burG::Kan* (1–10 µg). The transformed cells were then cultured in LB medium (1 ml) for 4 h at 30 °C with shaking and subsequently plated on LB-agar plates supplemented with tetracycline (45 µg ml^−1^) and kanamycin (150 μg ml^−1^). After 3 days, positive colonies were observed and confirmed by colony PCR with the primer pair Isomerase fw2 and Isomerase rv2 using Taq Polymerase (New England Biolabs). See Supplementary Fig. 9 for PCR verification.

### Preparation of His_6_-tagged BurC

The gene encoding BurC was amplified by PCR using the primer pair burC4-fw-NdeI and burC-rv-EcoRI. The obtained PCR product was subsequently ligated into the pCR2.1 vector. After sequence analysis (GATC biotech), the gene fragment was restricted with *Nde*I and *Eco*RI and then cloned into the *Nde*I and *Eco*RI sites of pET28a(+) vector, generating pET28a-*burC*. To prepare a seed culture, *E. coli* BL21 (DE3) pET28-*burC* was grown overnight in LB medium with 50 μg ml^−1^ of kanamycin. An aliquot (2.5 ml) of this seed culture was used to inoculate 250 ml LB medium supplemented with kanamycin (50 μg ml^−1^) in a 1 litre baffled Erlenmeyer flask. The culture was grown at 37 °C with orbital shaking until an OD_600_ of 0.5–0.7 was reached. Protein production was induced by addition of isopropylthiogalactoside (IPTG; 0.5 mM as a final concentration) followed by incubation at 15 °C for 18 h. Cells were harvested by centrifugation (9,000*g*, 4 °C, 10 min) and frozen at −20 °C until further use. For protein extraction, the cells were resuspended in 50 mM Tris–HCl (pH 8.0), 200 mM NaCl, 10% glycerol, 0.5 mM dithiothreitol, lysozyme (200 μg ml^−1^), and phenylmethylsulfonyl fluoride (100 μM), followed by incubation at 37 °C for 30 min. Then, the cells were lysed using a sonicator (Bandelin Sonopuls HD2200), and centrifuged (9,200*g*, 4 °C, 35 min) to obtain a clear lysate. The cleared lysate was loaded onto a Ni-IDA agarose (Biontex) column with manual handling. Equilibration and washing buffers were 50 mM Tris–HCl (pH 7.5), 500 mM NaCl, 10 mM imidazole and 30 mM imidazole, respectively; at least 10 column volumes were used for each step. The elution was carried out using a stepwise increase to 500 mM imidazole in the same buffer. The obtained fractions were analysed for target protein by SDS–polyacrylamide gel electrophoresis (SDS–PAGE; molecular weight of His_6_-BurC, 39.9 kDa). Protein containing fractions were dialysed against 20 mM sodium phosphate buffer (pH 7.2) and 200 mM NaCl, and concentrated using an Amicon Ultra-30K concentrator (Merck Millipore). See Supplementary Fig. 10 for SDS–PAGE analysis.

### Preparation of His_6_-tagged BurG

The gene encoding BurG was amplified by PCR using the primer pair burG-fw-NheI and burG-rv-HindIII. The obtained amplicon was ligated into the pJET1.2 vector and verified by sequence analysis (GATC biotech). Subsequently, the gene fragment was restricted with *Nhe*I and *Hin*dIII and then cloned into the *Nhe*I- and *Hin*dIII-restricted pET28a(+) vector, generating pET28a-*burG*. To prepare a seed culture, *E. coli* BL21 (DE3) pET28a-*burG* was grown overnight in LB medium supplemented with 50 μg ml^−1^ of kanamycin. An aliquot (2.5 ml) of this seed culture was used to inoculate 250 ml LB medium supplemented with kanamycin (50 μg ml^−1^) in a 1 litre baffled Erlenmeyer flask. The culture was grown at 37 °C with orbital shaking until an OD_600_ of 0.5–0.7 was reached. Protein production was induced by addition of IPTG (0.5 mM as final concentration), followed by incubation at 15 °C for 18 h. Cells were harvested by centrifugation (9,000*g*, 4 °C, 10 min) and frozen at −20 °C until further use. For protein extraction, the cells were resuspended in 50 mM Tris–HCl buffer (pH 8.0) supplemented with 200 mM NaCl, 10% glycerol, 0.5 mM dithiothreitol, lysozyme (1 mg ml^−1^) and PMSF (100 μM), followed by incubation at 37 °C for 30 min. Then, the cells were lysed using a sonicator (Bandelin Sonopuls HD2200), and centrifuged (9,200*g*, 4 °C, 35 min) to obtain a clear lysate. The cleared lysate was applied to a Ni-IDA agarose (Biontex) column with manual handling. Equilibration and washing buffers were 50 mM Tris–HCl (pH 7.5), 500 mM NaCl, 10 mM imidazole or 30 mM imidazole, respectively; at least 10 column volumes were used for each step. The elution was carried out using a stepwise increase to 500 mM imidazole in the same buffer. The obtained fractions were analysed for target protein by SDS–PAGE (molecular weight of His_6_-BurG, 40.6 kDa). Protein containing fractions were dialysed against 50 mM Tris–HCl (pH 7.5), 200 mM NaCl buffer and concentrated using an Amicon Ultra-30K concentrator (Merck Millipore). See Supplementary Fig. 10 for SDS–PAGE analysis.

### Generation of pET28a-*burG_E232Q*

A codon-optimized, point-mutated synthetic *burG E232Q* gene fragment was obtained from Integrated DNA Technologies. This gene fragment was ligated into a pJET1.2 vector (Thermo Fisher Scientific) and introduced into *E. coli* XL1 Blue by electroporation. The obtained plasmid pJET-*burG_E232Q* was verified by sequencing (GeneWiz) followed by restriction with *Hin*dIII and *Nhe*I. This obtained gene fragment was cloned into an *Hin*dIII- and *Nhe*I-restricted pET28a vector, yielding pET28-burG_E232Q. (See Supplementary Information for sequence.)

### Thermal stability of BurG and BurG E232Q

The thermal stability of BurG and BurG E232Q protein preparations was determined by using a Tycho NT.6 (NanoTemper Technologies) monitoring the fluorescence of each protein preparation at 330 and 350 nm during a thermal ramp. Inflection temperatures (*T*_i_ BurG, 60.5 ± 0.1 °C; *T*_i_ BurG E232Q, 61.85 ± 0.05 °C) were obtained in technical duplicates at a concentration of 70 µM enzyme supplemented with 2 mM MgSO_4_ and 4 mM NAD^+^.

### Dialysis of BurG preparations

A preparation of His_6_-tagged BurG was diluted with denaturing buffer (pH 8.0) to a final concentration of 8 M urea, 100 mM Tris–HCl and 200 mM NaCl. The resulting solution (1 ml) was pipetted into a dialysis bag (Spectra-Por, 3,500 dalton molecular weight cut-off, Spectrum Labs) and the bag was incubated in 3 litres of the same buffer as mentioned above at 4 °C for 24 h. Regular buffer exchange resulted in a final dilution factor of 6.4 × 10^17^. Next, the denaturing buffer was exchanged for refolding buffer (0.5 M l-arginine, 50 mM Tris–HCl and 100 mM NaCl, pH 8.0) followed by incubation for 12 h at 4 °C with regular buffer exchange to remove residual urea. This was followed by the removal of l-arginine through a further buffer exchange step to reaction buffer (50 mM Tris–HCl and 100 mM NaCl, pH 8.0) and incubation for 5 h at 4 °C with regular buffer exchange. Complete removal of NAD^+^ was validated by analysis of the refolded protein preparation via LC-HRMS. The resulting NAD^+^-free solution was subjected to further gonydiol transformation assays.

### Substrate saturation kinetics (spectrophotometry)

The reduction activity of BurG (E232Q) was measured with hydroxypyruvate and NADH/NADPH as substrates in varying concentrations in semi-micro disposable ultraviolet cuvettes (Brand) with a final reaction volume of 0.9 ml. Purified tag-free BurG (E232Q) as prepared for protein crystallization (1 µM final concentration) was added to Tris–HCl buffer (100 mM, pH 7.5) containing MgSO_4_ (10 mM) and NADH/NADPH and the resulting mixture incubated at 30 °C for 5 min in a UV-1800 spectrophotometer (Shimadzu). Subsequently, hydroxypyruvate was added, and the absorption at 340 nm was monitored spectrophotometrically during the incubation at 30 °C. Each substrate concentration was measured in duplicate with enzyme obtained from independent preparations. No decrease in signal was observed when NADPH was used instead of NADH under similar conditions. The molarity of NADH at each time point measured in 1 s intervals was calculated by using a calibration curve obtained on the same instrument. Michaelis–Menten parameters were determined with one substrate at a fixed concentration (2 mM hydroxypyruvate or 0.1 mM NADH) while varying the second substrate at concentrations ranging from 0.020 to 1.5 mM for hydroxypyruvate (0.020–9 mM in the case of BurG E232Q) and from 6.68 to 75 µM for NADH. Notably, substrate inhibition occurred when concentrations higher than 2.25 mM hydroxypyruvate were used with wild-type BurG. Initial velocities were determined from the slope of the NADH degradation divided by the enzyme concentration (*v*_0_/[*E*_0_]) and plotted against the respective substrate concentration. The obtained curves were fitted to the Michaelis–Menten equation by nonlinear regression using GraphPad Prism v.8.2.1. Kinetic parameters were determined for each enzyme batch separately, averaged and are given with their geometric mean and interval. See Supplementary Figs. 6 and 7 for obtained Michaelis–Menten plots and parameters. Concentrations of used protein solutions were determined on a NanoDrop One (Thermo Fisher Scientific) with a calculated extinction coefficient of 24,870 M^−1^ cm^−1^ for BurG in technical triplicates.

### Substrate saturation kinetics (LC-HRMS)

The kinetic parameters of the formation of **6** from **5** as catalysed by BurG were determined with the same Orbitrap LC-HRMS system as described below. Enzyme reactions consisted of Tris–HCl buffer (100 mM, pH 7.5), MgSO_4_ (10 mM), NAD^+^ (1 mM) and purified tag-free BurG as prepared for protein crystallization (1.5 µM final concentration), and were incubated in 1.6 ml glass vials with a glass insert at 30 °C in the autosampler of the ultra-high-performance liquid chromatography (UHPLC) system. To start the enzymatic reaction, synthetic *rac*-*anti*-**5** as TFA salt was added in varying concentrations (final concentration, 91.4–1041.7 µM; final reaction volume, 48 µl) and the resulting mixture was promptly and repeatedly (six time points per concentration) injected onto a Nucleoshell Bluebird 2.7, C_18_ column (50 × 2 mm, Macherey-Nagel) run under isocratic conditions (solvent, H_2_O + 0.1% HCOOH; run time, 1 min; column oven temperature, 40 °C; post-column cooler, 25 °C; flow rate, 0.45 ml min^−1^_;_ injection volume, 2 μl) for mass spectrometric analysis. The mass spectrometer was operated in positive selected ion monitoring mode with a scan range of 106.5–156.6 *m*/*z* and a resolution of 7,500. TFA (112.98559 *m*/*z*) was used as lock mass. The *m*/*z* of **6** (131.0350 ± 10 ppm) was monitored via the extracted ion chromatogram, the resulting chromatograms smoothed (gaussian, nine points) and the area under the curve determined via the Genesis Peak Detection algorithm in XCalibur Qual Browser v.4.3.73.11 software (Thermo Fisher Scientific). A calibration curve with **6** injected in varying concentrations (0.0023–0.133 mM) dissolved in a matrix-matched solution (100 mM Tris–HCl buffer pH 7.5, 10 mM MgSO_4_, 1 mM NAD^+^) was recorded on the same system in technical duplicates (standard weighed in independently) and used to convert the determined peak areas to concentrations. Initial velocities were determined from the slope of the formation of **6** divided by the enzyme concentration (*v*_0_/[*E*_0_]) and plotted against the respective substrate concentration. The obtained curves were fitted to the Michaelis–Menten equation by nonlinear regression using GraphPad Prism v.8.2.1. Kinetic parameters were determined for each enzyme batch separately, averaged and are given with their geometric mean and interval. To represent the kinetic parameters for the eutomer ((2*R*,3*R*)*-***5**), the averaged *K*_M_ was divided by two. Concentrations of BurG preparations were determined on a NanoDrop One (Thermo Fisher Scientific) as mentioned above.

### Metabolite extraction for comparative LC-HRMS analysis

*B*. *thailandensis Pbur* and *B*. *thailandensis Pbur burG::Kan* were independently grown in 300 ml baffled Erlenmeyer flasks filled with 100 ml of MM9 medium that had been supplemented with either tetracycline (45 μg ml^−1^) or tetracycline (45 μg ml^−1^) plus kanamycin (150 μg ml^−1^) at 30 °C with orbital shaking for 24 h. Subsequently, the cells were harvested from a 50 ml aliquot by centrifugation (8,000*g*, for 15 min). The resulting cell pellet was resuspended in 25 ml methanol, sonicated and subsequently incubated for 60 min at room temperature. The centrifugation and extraction steps were repeated a second time and both obtained fractions were combined and concentrated in vacuo to yield crude extracts. These extracts were dissolved in 4 ml of methanol, and 100 µl of the resulting solution was then diluted with 200 µl methanol and filtered through a PTFE syringe filter. Subsequently the extracts were subjected to LC-HRMS analysis for a metabolomics analysis using the software Compound Discoverer 2.1 SP1 (Thermo Fisher Scientific). Both genotypes were analysed using a metabolomics workflow with and without a pattern scoring node to filter for sulfur-containing metabolites. The obtained metabolic profiles were compared using differential analysis.

### Chemical complementation of *B. thailandensis Pbur burG*::*Kan*

Trigonic acid **6** (400 µM final concentration) was added to MM9 medium (75 ml in a 300 ml baffled Erlenmeyer flask) supplemented with tetracycline (45 μg ml^−1^) and kanamycin (150 μg ml^−1^) and inoculated with a preculture of *B. thailandensis Pbur burG::Kan* to a final OD_600_ of 0.1. Controls consisted of *B. thailandensis Pbur* (omitting the kanamycin) and *B. thailandensis Pbur burG::Kan* without addition of **6** prepared in the same way. All cultures were incubated at 30 °C with orbital shaking for 24 h. The obtained cultures were extracted with EtOAc three times, and the organic phase dried over Na_2_SO_4_ and concentrated under reduced pressure. The obtained crude extracts were redissolved in methanol, filtered through a PTFE syringe filter and subjected to LC-HRMS. Similar results were obtained on independent days with independent starting cultures (biological triplicates in total).

### In vitro hydroxylation of gonyol

Purified His_6_-BurC (400 nM final concentration) was added to Tris–HCl buffer (50 mM, pH 7.5) containing ascorbate (0.1 mM), α-ketoglutarate (1 mM) and FeSO_4_ (0.1 mM). The reaction was started by addition of *R*- or *S*-gonyol (**4**; each as TFA salt; 150 µM final concentration). As a control, purified His_6_-BurC was heat-inactivated at 80 °C for 25 min and used in the same way. The resulting mixtures were incubated at 30 °C and aliquots taken at various time points (20, 50, 180, 210 and 360 min). Each aliquot was diluted with the same volume of methanol (typically 12.5 µl), filtered through a PTFE syringe filter and directly subjected to LC-HRMS analysis (Extended Data Fig. 2). Similar results were obtained on independent days with independent protein preparations (biological triplicates in total).

### In vitro cyclopropanol synthesis

Purified His_6_-BurG or His_6_-BurG E232Q (5 µM final concentration) were added to Tris–HCl buffer (100 mM, pH 7.5) containing MgSO_4_ (2 mM) and NAD^+^ (1 mM) and the resulting mixture incubated at 30 °C for 5 min. Subsequently *anti*-, *syn*- or natural gonydiol (**5**; each as TFA salt; 1.3 mM final concentration) were added followed by incubation at 30 °C with aliquots taken at various time points (typically after 60 min). Each aliquot was diluted with the same volume of methanol, then filtered through a PTFE syringe filter and directly subjected to LC-HRMS analysis.

Mg^2+^ can be substituted with Co^2+^ (in the form of CoSO_4_) to achieve formation of trigonic acid. However, Mg^2+^ cannot be substituted with Mn^2+^ or Zn^2+^. Sole addition of Mg^2+^ suffices (when no dialysis under denaturing conditions is performed during enzyme purification) to observe enzymatic transformations and no addition of NAD^+^ is necessary. However, NAD^+^ was always detected via LC-HRMS in BurG preparations unless dialysis under denaturing conditions followed by refolding of the protein were performed (see above). Dialysed and thus NAD^+^-free BurG was used in gonydiol transformation assays as outlined above with and without addition of NAD^+^ or NADH. Assays performed with tag-free BurG/BurG E232Q as prepared for protein crystallography (Protein crystallography) yielded similar results as observed for His_6_-tagged BurG. Similar results were obtained on independent days with independent protein preparations (assay repeated more than three times in biological replicates).

### Headspace SPME–GC–MS detection of DMS

Purified tag-free BurG as prepared for protein crystallization (5 µM final concentration) was added to Tris–HCl buffer (100 mM, pH 7.5) containing MgSO_4_ (2 mM) and NAD^+^ (1 mM). Subsequently synthetic *rac*-*anti*-gonydiol **5** (as TFA salt, 2 mM final concentration) was added to give a final volume of 200 µl. The resulting reaction mixture was pipetted into a 1.5 ml Eppendorf tube without lid which was placed inside a 20 ml headspace vial. The vial was sealed and incubated at 30 °C for 80 min. A control reaction was carried out in the same way as detailed above with enzyme preparation that had been denatured at 80 °C for 20 min. For headspace SPME a carbon wide range/polydimethylsiloxane fibre 95 µm thick and 10 mm long (Thermo Fisher Scientific) was used. The fibre was incubated at 260 °C for 4 min prior to headspace extraction. The headspace vial was incubated at 70 °C for 5 min followed by headspace extraction with the fibre for 5 min at 70 °C. For analysis, a TRACE 1310 gas chromatograph (Thermo Fisher Scientific) fitted with a BPX5 capillary column, 30 m × 0.25 mm × 0.25 µm (Trajan) coupled to a TSQ 900 mass spectrometer (Thermo Fisher Scientific) was used. The SSL injector was equipped with a topaz split liner (1 mm × 6.3 mm × 78.5 mm, Restek) heated to 250 °C and used in splitless mode. Helium was used as carrier gas at a flow rate of 0.6 ml min^−1^. The GC oven was set to a temperature of 30 °C with a hold time for 4 min followed by heating to 200 °C for 3.4 min and a subsequent hold time at 200 °C for 2.6 min. The source and transfer line temperatures of the mass spectrometer were 230 and 250 °C, respectively. The scan range of the mass spectrometer was set to 10–300 *m*/*z*. DMS was detected via the extracted ion chromatogram at an *m*/*z* of 62, and its identity validated through a database search (NIST 2.4) of the obtained electron ionization spectrum and through its identical retention time when compared with a commercial standard.

### Isolation of gonydiol 5

Cells obtained from a 2 litre culture of *B. thailandensis Pbur burG::Kan* were extracted with methanol (2 × 200 ml) and the solvent subsequently removed under reduced pressure. The obtained crude extract was then redissolved in H_2_O (300 ml) to form a turbid solution which was pelleted by centrifugation at 6,000*g* for 45 min to yield a clear yellow solution. This solution was then loaded onto 100 g of the cationic exchange resin Amberlite IR-120 in the H^+^ state. After washing with 300 ml of H_2_O, bound cations were eluted with 1 litre of HCl (1 M) and the solvent removed through lyophilization. The concentrated eluate was subsequently redissolved in water and further purified via repeated rounds of preparative HPLC using a Phenomenex Synergi Fusion-RP C_18_ column (80-4, 250 × 21.2 mm) with H_2_O + 0.1% TFA as mobile phase at a flow rate of 20 ml min^−1^. Fractions containing the target molecule (as judged by NMR and LC-HRMS analysis) were pooled, concentrated in vacuo and further purified via repeated rounds of preparative HPLC with a SeQuant ZIC-HILIC column (200-5, 250 × 10 mm, Merck) (solvent A, 98% H_2_O, 2% CH_3_CN + 0.1% HCOOH, pH 2.7; solvent B, 10% H_2_O, 90% CH_3_CN, 5 mM NH_4_OAc; isocratic conditions: 71% B; flow rate, 5 ml min^−1^). Fractions containing the target molecule were pooled, concentrated under reduced pressure and analysed via ^1^H NMR spectroscopy. We noted a distinct difference in the ^1^H NMR spectra when fractions that were obtained with TFA in the mobile phase were compared to fractions that were obtained with NH_4_OAc/HCOOH as additive. While the TFA salt of **5** showed two signals resonating at 4.19 ppm (1H) and 4.01 ppm (1H), respectively, both signals merged to one signal resonating at 3.91 ppm (2H) when no TFA was present. As it seemed reasonable that a separation of these signals would aid structure elucidation, we converted the obtained substance to its TFA salt by repeatedly (3×) redissolving it in 1 ml of 1% TFA and subsequent removal of the solvent in vacuo. Through this we obtained 2.3 mg of the TFA salt of **5** as colourless oil (1.15 mg l^−1^). [*α*]_D_^20^ = − 3.5 (*c* = 0.36, MeOH); ^1^H NMR (600 MHz, CD_3_OD): see Supplementary Table 1; ^13^C NMR (151 MHz, CD_3_OD): see Supplementary Table 1; HRMS: calculated for C_7_H_15_O_4_S ([M + H]^+^), 195.0686; found, 195.0688.

### LC-HRMS

LC-HRMS measurements were carried out with an UltiMate 3000 UHPLC (Thermo Fisher Scientific) coupled to a Thermo Fisher Scientific QExactive HF-X Hybrid Quadrupole-Orbitrap equipped with an electrospray ion source.

For general analysis, bacterial extracts were analysed by using a Kinetex 100-1.7 C_18_ column (50 × 2.1 mm, Phenomenex) and an elution gradient (solvent A, H_2_O + 0.1% HCOOH; solvent B, CH_3_CN; 5% B to 100% B in 4.5 min, 100% B for 2 min, 100% B to 5% B in 0.001 min, 5% B for 1.5 min; flow rate, 0.7 ml min^−1^, injection volume, 2 μl). For metabolomics analysis, extracts were analysed in positive-ion mode by using a Nucleoshell Bluebird 2.7 C_18_ column (50 × 2 mm, Macherey-Nagel) and an elution gradient (solvent A, H_2_O + 0.1% HCOOH; solvent B, CH_3_CN; 0% B for 1 min, 0% B to 98% B in 6 min, 98% B for 3 min, 98% B to 0% B in 0.01 min, 0% B for 2.99 min; flow rate, 0.4 ml min^−1^_,_ injection volume, 2 μl).

Enzyme assays were analysed on a Nucleodur HTec 100-2 C_18_ column (100 × 2 mm, Macherey-Nagel) with an elution gradient (solvent A, H_2_O + 0.1% HCOOH; solvent B, CH_3_CN; 5 %B for 0.5 min, from 5% B to 100% B in 6.5 min, 100% B for 3 min, 100% B to 5% B in 0.01 min, 5% B for 2.99 min; flow rate, 0.4 ml min^−1^, injection volume, 2 μl).

Detection of NAD^+^ (Supplementary Fig. 5) was carried out by using a Luna Omega PS 100-1.6 C_18_ column (100 × 2.1 mm, Phenomenex) with an elution gradient (solvent A, H_2_O + 0.1% HCOOH; solvent B, CH_3_CN; 0% B for 0.5 min, from 0% B to 99% B in 6.5 min, 99% B for 3 min, 100% B to 0% B in 0.01 min, 5% B for 2.9 min; flow rate, 0.4 ml min^−1^_,_ injection volume, 2 μl).

Chiral LC-HRMS analysis was carried out by using an Astec CHIROBIOTIC R column (100 × 4.6 mm, Supelco) at a constant temperature of 20 °C (solvent A, 33.3 mM NH_4_OAc; solvent B, MeOH; isocratic conditions: 75% B; flow rate, 0.5 ml min^−1^).

### NMR

NMR spectra were measured on Bruker Avance II 300 MHz, Bruker Avance III 500 MHz and Bruker Avance III 600 MHz spectrometers (600 MHz with cryo probe) in CD_3_OD and CD_3_CN. Spectra were referenced relative to the residual solvent peak (CD_3_OD: *δ*_H_ = 3.30, *δ*_C_ = 49.0 ppm; CD_3_CN: *δ*_H_ = 1.94, *δ*_C_ = 1.3; 118.3 ppm).

### Phylogenetic analysis

Sequences for KARI family proteins were obtained from the NCBI server (https://www.ncbi.nlm.nih.gov/) by Protein BLAST using the databases of non-redundant protein sequences, UniProtKB/Swiss-Prot and PDB. The respective amino acid sequences were aligned using the MAFFT server (https://mafft.cbrc.jp/alignment/server/)^40^ with MAFFT v.7 and default settings. An unrooted fast maximum-likelihood-based tree was generated using IQ-TREE (https://www.hiv.lanl.gov/content/sequence/IQTREE/iqtree.html)^41^ with automatic model selection mode (ModelFinder)^42^, where the LG+G4 was selected. Phylogenetic bootstrap analysis was performed by ultrafast approximate bootstrap with 1,000 bootstrap replicates^43^. The obtained tree was displayed using MEGA7^44^. See Supplementary Fig. 4 and Table 6 for detailed information.

### Reagents, chemical synthesis and compound characterisation

Unless stated otherwise, all reagents were purchased from Sigma-Aldrich or TCI. The procedures for the preparation of synthetic compounds used in this study, and the spectral data, can be found in the Supplementary Information. For preparation of dimethyldioxirane, see ref. ^45^.

### Statistics and reproducibility

Comparative LC-HRMS analysis: biological triplicates (Fig. 2a and Supplementary Fig. 1).

Chemical complementation of gene inactivation mutant with trigonic acid and gene inactivation mutant analysis: biological triplicates (Fig. 2b).

In vitro hydroxylation of gonyol: technical triplicates (Fig. 2d and Extended Data Fig. 2).

Headspace SPME–GC–MS detection of DMS: performed once (Fig. 2e).

In vitro cyclopropanol synthesis: assay repeated more than three times with independent protein preparations (Fig. 2h).

Chiral LC-HRMS of cyclopropanol formation to determine stereochemistry of natural trigonic acid: technical triplicates (Extended Data Fig. 4).

Determination of enantiomeric excess of synthetic *R*-trigonic acid: technical triplicates (Extended Data Fig. 4).

NAD^+^ detection in BurG preparations: biological triplicates (Supplementary Fig. 5).

Substrate saturation kinetics; spectrophotometry (**8** as substrate): BurG: biological duplicates (Fig. 3c and Supplementary Fig. 6); BurG E232Q: technical duplicates (Supplementary Fig. 7); LC-MS (**5** as substrate): biological duplicates (Fig. 2i).

Protein concentration determination: technical triplicates for each preparation.

In vitro cyclopropanol synthesis with BurG E232Q: biological duplicates (Fig. 3h)

Thermal stability of proteins: technical duplicates.

### Protein crystallography

#### Culture conditions

For protein production to be used in protein crystallization experiments, *E. coli* transformed with a plasmid containing BurG or BurG E232Q was grown in Fernbach shaking flasks at 37 °C containing 3 litres of lysogeny broth supplemented with kanamycin (50 mg l^−1^). At an OD_600_ of 0.6, IPTG was added (final concentration 1 mM), and incubation was continued overnight at 20 °C. Subsequently, the cells were harvested by centrifugation (8,000*g*, 20 min), washed with 0.9% (w/v) NaCl and stored at −20 °C.

#### Protein purification for crystallization

Frozen bacterial cell mass (25 g) obtained as detailed above (Culture conditions) was thawed in 50 ml of 100 mM Tris–HCl, pH 7.5, containing 500 mM NaCl and 20 mM imidazole/HCl (buffer A). Subsequently, the cells were disrupted by sonification (Branson Digital Sonifier 250, G. Heinemann) and the resulting suspension centrifuged at 40,000*g* for 20 min at 4 °C in a Sigma 4K15 centrifuge. The supernatant was loaded onto a 5 ml HisTrap HP column (GE Healthcare), which had been equilibrated with buffer A (flow rate, 5 ml min^−1^) using an ÄKTA Pure system (GE Healthcare). Unbound or loosely associated proteins were removed by washing with buffer A. BurG protein was eluted by applying a 50 ml linear gradient from buffer A to buffer B (100 mM Tris–HCl, pH 7.5, 500 mM NaCl, 500 mM imidazole). Fractions were combined and 200 U of thrombin from bovine plasma (Serva) was added. The solution was dialysed overnight at 4 °C against 20 mM Tris–HCl, pH 7.5, containing 100 mM NaCl and again applied to the HisTrap HP column equilibrated with buffer A. The percolate was concentrated to 1 ml and the solution was applied to a HiLoad 16/60 Superdex 75 pg column (GE Healthcare; flow rate, 1.5 ml min^−1^). Single peak fractions were concentrated to 40 mg ml^−1^ using a 10K Amicon Ultra Centrifugal Filter Device (Millipore), and stored at 4 °C. Analytical size exclusion chromatography was carried out with a Superdex 75 Increase 10/300 GL column (GE Healthcare; flow rate, 1 ml min^−1^). See Supplementary Fig. 11 for SDS–PAGE analysis.

#### Figure illustration of structural images

The electron densities represent F_o_–F_c_ maps (contoured to 3*σ*) with the respective ligands omitted for phasing. The following colour code was used: carbon atoms of subunit A (grey), subunit B (pink), E323Q-mutant (dark pink), NAD^+^ (gold), NADH (golden rod), **6** (brown) **7** (light blue), **8** (orchid), **11** (olive), **12** (cyan, carbon atom for the substituted sulfur atom in dark green), **13** (dark cyan, carbon atom for the substituted sulfur atom in dark green), **14** (light green), **15** (purple), DMS (brown). Nitrogen, oxygen and sulfur atoms are coloured in blue, red and yellow, respectively. The two magnesium atoms (named A and B) are shown as green spheres, water molecules are presented as red balls. Hydrogen bonds are drawn as black dots. Figures were created with the programs Bobscript^46^ and PyMOL v.2.3 (Schrödinger).

#### Protein crystallization

Crystallization experiments of BurG variants (17.5 mg ml^−1^) were performed using the sitting drop vapour diffusion method at 20 °C. For co-crystallization experiments, the appropriate ligand (100 mM stock solution in H_2_O) was added to BurG wild-type or mutated BurG to a final concentration of 2 mM. Crystallization droplets had a maximum volume of 0.4 µl with a ratio of either 1:1, 2:1 or 3:1 of protein and reservoir solution. BurG showed a strong crystallization preference for polyethylene glycol. Unfortunately, the majority of crystals had disordered layers. Therefore, suitable candidates with optimal lattice packing had to be identified by diffraction measurements. (See Supplementary Information for distinct crystallization parameters.) Crystals were cryoprotected by a 7:3 mixture of mother liquor and 100% (v/v) glycerol and subsequently vitrified in liquid nitrogen.

#### Protein structure determination

High-resolution datasets of BurG variants were recorded with synchrotron radiation of *λ* = 1.0 Å at the beamline X06SA, Swiss Light Source (SLS), Paul Scherrer Institute, Villigen, Switzerland. Reflection intensities were evaluated with the program package XDS and data reductions were carried out with XSCALE^47^ (Supplementary Table 2). The resolution limits were determined based on the following criteria: *I*/*σ*(*I*) > 2.0, *R*_merge_ < 60% and redundancy >3.0. First, we solved the BurG (holo) structure by Patterson search calculations applying PHASER^48^ and coordinates of the keto acid reductoisomerase from *Staphylococcus aureus* (PDB 6AQJ)^19^. Using COOT^49^ in combination with REFMAC^50^, the BurG sequence was built in iterative rounds. The model was completed with the cofactors NAD^+^ and Mg^2+^. Water molecules were automatically placed with ARP/wARP solvent^51^. Restrained and TLS (Translation/Libration/Screw) REFMAC refinements yielded excellent *R*_work_ and *R*_free_ values as well as root-mean-square deviation values of bond lengths and angles. For all other datasets BurG (holo) served as starting model for initial phasing. Model building and structural refinement followed the same procedure (Supplementary Table 2). Interestingly, the high-resolution electron density map at 1.6 Å in PDB 7PCN depicts a mixture of **14** and NADH as well as **6**, DMS and NAD^+^ at the active site. Note that **14** and NADH as well as **6**, DMS and NAD^+^ were incorporated with an occupancy of 0.5 each. In the data set PDB 7PCO (BurG_E232Q mutant), enol-oxaloacetate **15** was co-purified from the *E. coli* lysate. These findings are in contrast to BurG (wild-type) and show that the E232Q BurG mutant has a high affinity for the metabolite. All crystal structures have been deposited in the RCSB Protein Data Bank. See Supplementary Table 2 for molprobity^52^ clash scores.

### Reporting summary

Further information on research design is available in the Nature Research Reporting Summary linked to this article.

## Online content

Any methods, additional references, Nature Research reporting summaries, source data, extended data, supplementary information, acknowledgements, peer review information; details of author contributions and competing interests; and statements of data and code availability are available at 10.1038/s41557-022-01005-z.

## Supplementary information

Supplementary Information Synthetic procedures, distinct crystallization parameters, nucleotide sequence of synthetic *burG E232Q*, Supplementary Figs. 1–35, Tables 1–6 and references.

Reporting Summary

Supplementary Data 1 Source Data for the Supplementary Information

## Data Availability

NMR raw files of natural gonydiol and MS raw files used for comparative metabolomics analysis (Fig. 2a and Supplementary Fig. 1) are deposited on Zenodo. (10.5281/zenodo.6554506) and are available without restrictions. All other data are available in the main text, the Supplementary Information, the respective source data files or via the RCSB PDB (7PCC, 7PCE, 7PCG, 7PCI, 7PCL, 7PCM, 7PCN, 7PCO, 7PCT). Source data are provided with this paper.

## Source data

Source Data Fig. 2 Source Data for Figure 2
Source Data Fig. 3 Source Data for Figure 3
Source Data Extended Data Fig. 2 Source Data for Extended Data Figure 2
Source Data Extended Data Fig. 3 Source Data for Extended Data Figure 3
Source Data Extended Data Fig. 4 Source Data for Extended Data Figure 4
