# Supplementary material for: Pathogenic bacteria remodel central metabolic enzyme to build a cyclopropanol warhead
Source: Nat Chem. 2022 Jul 29;14(8):884–90. doi: 10.1038/s41557-022-01005-z (PMC9359912; doi:10.1038/s41557-022-01005-z)
Supplement: Supplementary file 1 — Synthetic procedures, distinct crystallization parameters, nucleotide sequence of synthetic burG E232Q, Supplementary Figs. 1–35, Tables 1–6 and references. [file 41557_2022_1005_MOESM1_ESM.pdf]

nature portfolio

https://doi.org/10.1038/s41557-022-01005-z

Supplementary information

Pathogenic bacteria remodel central metabolic enzyme to build a cyclopropanol warhead

In the format provided by the authors and unedited

Supplementary information

Pathogenic Bacteria Remodel Central Metabolic Enzyme to Build a Cyclopropanol Warhead

Authors: *Felix Trottmann[1], Keishi Ishida[1], Mie Ishida-Ito[1], Hajo Kries[2], Michael Groll*[3], Christian Hertweck*[1,4]*

Affiliations:

[1]Department of Biomolecular Chemistry, Leibniz Institute for Natural Product Research and Infection Biology – HKI, 07745 Jena, Germany.

[2]Junior Research Group Biosynthetic Design of Natural Products, Leibniz Institute for Natural Product Research and Infection Biology – HKI, 07745 Jena, Germany.

[3]Center for Protein Assemblies, Chemistry Department, Technical University Munich, 85748 Garching, Germany.

[4]Faculty of Biological Sciences, Friedrich Schiller University Jena, 07743 Jena, Germany.

*Correspondence to: michael.groll@tum.de (M.G.); christian.hertweck@leibniz-hki.de (C.H.)

Content

Synthetic procedures

Distinct crystallisation parameters

Nucleotide sequence of synthetic *burG E232Q*

Supplementary Figures (including spectral data)

Supplementary Figure 1. Comparative metabolomics analysis.

Supplementary Figure 2. Synthesis of gonydiol.

Supplementary Figure 3. HR-MS^2 spectra of trigonic acid.

Supplementary Figure 4. Extended phylogenetic tree.

Supplementary Figure 5. Detection of NAD^+ in denatured preparations of BurG.

Supplementary Figure 6. Michaelis Menten kinetics of BurG mediated reductions with hydroxypyruvate and NADH as substrates.

Supplementary Figure 7. Michaelis Menten kinetics of BurG E232Q mediated reductions with hydroxypyruvate and NADH as substrates.

Supplementary Figure 8. Complex structure of mutant BurG E232Q with bound enol-oxaloacetate.

Supplementary Figure 9. Verification of mutant strain.

Supplementary Figure 10. His_6-tagged enzyme preparations.

Supplementary Figure 11. Tag-free enzyme preparations

Supplementary Figure 12. ^1H NMR spectrum of natural gonydiol (**5**).

Supplementary Figure 13. Zoom into ^1H NMR spectrum of natural gonydiol (**5**).

Supplementary Figure 14. ^{13}C NMR spectrum of natural gonydiol (**5**).

Supplementary Figure 15. COSY spectrum of natural gonydiol (**5**).

Supplementary Figure 16. HSQC spectrum of natural gonydiol (**5**).

Supplementary Figure 17. HMBC spectrum of natural gonydiol (**5**).

Supplementary Figure 18. ^1H NMR spectrum of methyl (E)-5-dimethylsulfonio-pent-2-enoate.

Supplementary Figure 19. ^{13}C NMR spectrum of methyl (E)-5-dimethylsulfonio-pent-2-enoate.

Supplementary Figure 20. ^1H NMR spectrum of methyl *anti*-2,3-dihydroxy-5-dimethylsulfonio-pentanoate.

Supplementary Figure 21. ^{13}C NMR spectrum of methyl *anti*-2,3-dihydroxy-5-dimethylsulfonio-pentanoate.

Supplementary Figure 22. ^1H NMR spectrum of *anti*-gonydiol.

Supplementary Figure 23. ^{13}C NMR spectrum of *anti*-gonydiol.

Supplementary Figure 24. ^1H NMR spectrum of *syn*-gonydiol.

Supplementary Figure 25. ^{13}C NMR spectrum of *syn*-gonydiol.

Supplementary Figure 26. ^{1}H NMR spectrum of *R*-gonyol.

Supplementary Figure 27. ^{13}C NMR spectrum of *R*-gonyol.

Supplementary Figure 28. ^{1}H NMR spectrum of *S*-gonyol.

Supplementary Figure 29. ^{13}C NMR spectrum of *S*-gonyol.

Supplementary Figure 30. ^{1}H NMR spectrum of carba-gonydiol.

Supplementary Figure 31. ^{13}C NMR spectrum of carba-gonydiol.

Supplementary Figure 32. ^{1}H NMR spectrum of trigonic acid (**6**).

Supplementary Figure 33. ^{13}C NMR spectrum of trigonic acid (**6**).

Supplementary Figure 34. ^{1}H NMR spectrum of ethyl *R*-2-hydroxy-2-(1-hydroxycyclopropyl)acetate.

Supplementary Figure 35. ^{13}C NMR spectrum of ethyl *R*-2-hydroxy-2-(1-hydroxycyclopropyl)acetate.

Supplementary Tables

Supplementary Table 1. NMR shifts of gonydiol.

Supplementary Table 2. X-ray data collection and refinement statistics.

Supplementary Table 3. Bacterial strains used in this study.

Supplementary Table 4. Primers used in this study.

Supplementary Table 5. Vectors and plasmids used in this study.

Supplementary Table 6. KARI sequences used for phylogenetic analysis.

References

Synthetic procedures

Methyl (*E*)-5-dimethylsulfonio-pent-2-enoate

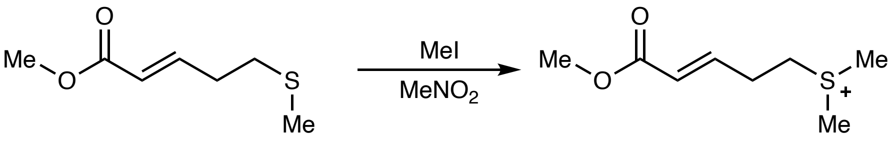

Iodomethane (713.6 mg, 5 mmol, 2.5 eq.) was added to a solution of methyl (*E*)-5-(methylthio)pent-2-enoate (321.0 mg, 2 mmol, 1 eq., obtained from Enamine Ltd., Kiev, Ukraine, product EN300-4784758) dissolved in 2.7 mL nitromethane and the resulting mixture stirred at room temperature for 20 h. Subsequently, the solvent was removed under reduced pressure. The residue was purified by preparative HPLC to remove the iodide counterion using a Phenomenex Synergi Fusion-RP C_{18} column (80-4, 250 × 21.2) and an elution gradient [solvent A: H_2O + 0.1% TFA, solvent B: CH_3CN 83%, 0% B for 10 min, from 0% to 100% B in 35 min; flow rate: 15 mL min^{-1}]. Through this, we obtained 523.3 mg of methyl (*E*)-5-(dimethylsulfonio)pent-2-enoate as TFA salt (89%, colourless oil). To check for iodide, 4.5 mg of the obtained product was dissolved in 0.5 mL of H_2O, and 0.1 mL of a $AgNO_3$ solution (0.1016 N) was added. A slight precipitate formed which fully dissolved when concentrated NH_4 solution was added, indicating that no residual iodide was present in the sample.

^{1}H NMR (500 MHz, CD_3OD): δ 6.93 (dt, *J* = 15.8, 6.9 Hz, 1H), 6.09 (dt, *J* = 15.8, 1.5 Hz, 1H), 3.73 (s, 3H), 3.46 (t, *J* = 7.6 Hz, 2H), 2.94 (s, 6H), 2.78 (m, 2H).

^{13}C NMR (126 MHz, CD_3OD): δ 167.7, 144.3, 125.2, 52.2, 42.8, 27.3, 25.2.

HRMS: Calculated for $C_8H_{15}O_2S$, ($[M]^+$): 175.0787; found: 175.0786.

Methyl *anti*-2,3-dihydroxy-5-dimethylsulfonio-pentanoate

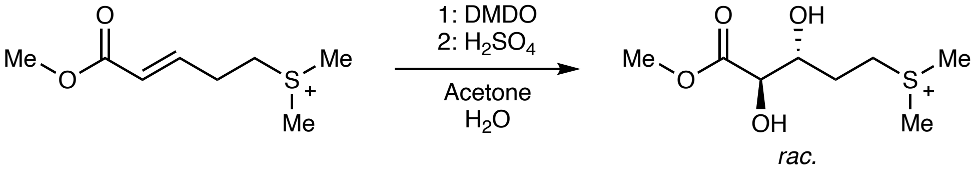

Dimethyldioxirane (DMDO) was prepared according to a known procedure[45] and obtained with a concentration of 50 mM. 4.8 mL of this DMDO solution (17.8 mg, 0.24 mmol, 2.2 eq) in acetone were added to 4.8 mL of an aqueous solution of the TFA salt of methyl (*E*)-5-(dimethylsulfonio)pent-2-enoate (32.6 mg, 0.11 mmol, 1 eq) and the resulting solution stirred for 16 h at room temperature. Subsequently 0.5 mL of H_2SO_4 (1 M) were added, followed by removal of the solvent under reduced pressure at 50 °C. The obtained residue was purified via preparative HPLC using a Nucleodur PolarTec 100-5 C_{18} column (250 × 10 mm, Macherey-Nagel) with H_2O + 0.1% TFA as mobile phase at a flow rate of 4 mL min^{-1}. After removal of the solvent *in vacuo* we obtained 12.3 mg (34%, colourless oil) of methyl *anti*-2,3-dihydroxy-5-dimethylsulfonio-pentanoate as racemic TFA salt.

^{1}H NMR (300 MHz, CD_3OD): δ 4.13 (d, *J* = 5.2, 1H), 3.93 (m, 1H), 3.76 (s, 3H), 3.51–3.33 (m, 2H), 2.91 (s, 3H), 2.89 (s, 3H), 2.14–1.95 (m, 2H).

^{13}C NMR (76 MHz, CD_3OD): δ 174.2, 75.6, 72.3, 52.6, 42.5, 27.8, 25.9, 25.4.

HRMS: Calculated for $C_8H_{17}O_4S$, ($[M]^+$): 209.0842; found, 209.0841.

***anti*-Gonydiol (*rac-anti*-5)**

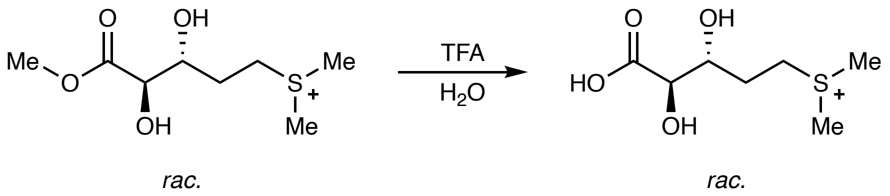

Methyl *anti*-2,3-dihydroxy-5-dimethylsulfonio-pentanoate (TFA salt, 6.1 mg, 0.019 mmol) was dissolved in 1.3 mL of H_2O, 0.78 mL of TFA were added, and the resulting solution was heated to 80 °C with stirring for 16 h. Subsequently the solvent was removed under reduced pressure and the residue purified via preparative HPLC using a Nucleodur PolarTec 100-5 C_{18} column (250 × 10 mm, Macherey-Nagel) with H_2O + 0.1% TFA as mobile phase at a flow rate of 4 mL min^{-1}. After concentration of the pure fraction *in vacuo* we obtained 4.8 mg of the TFA salt of *anti*-**5** (81%, colourless oil) in its racemic form.

^{1}H NMR (600 MHz, CD_3OD): δ 4.16 (d, *J* = 4.4, 1H), 3.98 (m, 1H), 3.48–3.37 (m, 2H), 2.91 (s, 3H), 2.89 (s, 3H), 2.13–2.0 (m, 2H).

^{13}C NMR (151 MHz, CD_3OD): δ 175.3, 75.3, 72.3, 42.6, 27.4, 25.9, 25.4.

HRMS: Calculated for $C_7H_{15}O_4S$, ($[M+H]^+$): 195.0686; found: 195.0686.

***syn*-Gonydiol (*rac*-*syn*-5)**

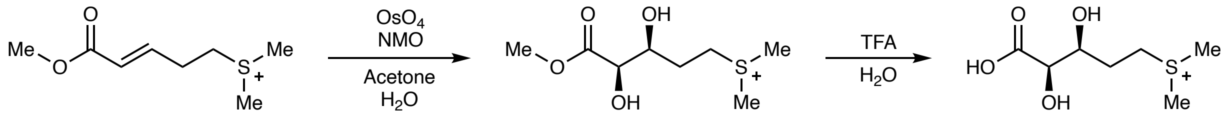

Methyl (*E*)-5-(methylthio)pent-2-enoate (TFA salt, 23,8 mg, 0.083 mmol, 1 eq.) was dissolved in a mixture of acetone/H_2O (7:3, 0.2 mL), 0.024 mL of an *N*-methylmorpholine-*N*-oxide (NMO) solution (470 mg mL^{-1}, 11.3 mg, 0.097 mmol, 1.2 eq.), and 2.5 µL of an OsO_4 solution (4% in H_2O, 100 µg, 393 nmol, 0.5 mol-%) were added and the solution stirred for 16 h at room temperature. After quenching with 20 mg of Na_2SO_3 the aqueous solution was extracted with EtOAc (3 × 2 mL) and the solvent removed under reduced pressure. Purification by preparative HPLC using a Nucleodur PolarTec 100-5 C_{18} column (250 × 10 mm, Macherey-Nagel) with H_2O + 0.1% TFA as mobile phase at a flow rate of 5 mL min^{-1} yielded 18 mg of the TFA salt of methyl *syn*-2,3-dihydroxy-5-dimethylsulfonio-pentanoate (colourless oil) in a semipure form. Methyl *syn*-2,3-dihydroxy-5-dimethylsulfonio-pentanoate (TFA salt, 12.4 mg, 0.038 mmol) was then dissolved in 2.6 mL of H_2O, 1.6 mL of TFA were added and the resulting solution heated to 80 °C with stirring for 4 h. Subsequently the solvent was removed under reduced pressure and the residue purified via preparative HPLC with a SeQuant ZIC-HILIC column (200-5, 250 × 10, Merck) [solvent A: 98% H_2O, 2% CH_3CN + 0.1% FA, pH 2.7 solvent B: 10% H2O, 90% CH_3CN, 5 mM NH_4OAc isocratic conditions: 71% B; flow rate: 5 mL min^{-1}]. After concentration under reduced pressure, the compound was converted to its TFA salt by repeatedly (3 ×) redissolving it in 1 mL of 1% TFA and subsequent removal of the solvent *in vacuo*. Through this we obtained 9.3 mg of the TFA salt of racemic *syn*-**5** as colourless oil (53% over two steps).

^1H NMR (600 MHz, CD_3OD): δ 4.07 (d, *J* = 2.9, 1H), 4.04 (m, 1H), 3.48–3.37 (m, 2H), 2.91 (s, 3H), 2.90 (s, 3H), 2.16–2.06 (m, 2H).

^{13}C NMR (151 MHz, CD_3OD): δ 175.9, 74.7, 72.0, 42.6, 29.0, 25.9, 25.4.

HRMS: Calculated for $C_7H_{15}O_4S$, ($[M+H]^+$): 195.0686; found: 195.0685.

***R*-Gonyol (*R*-4)**

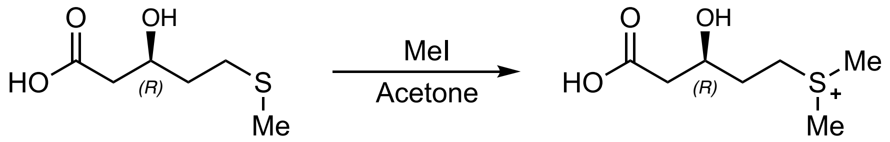

(*R*)-3-Hydroxy-5-(methylthio)pentanoic acid (117 mg, 0.713 mmol, 1 eq.) as obtained from Enamine Ltd. (Kiev, Ukraine, product EN300-1807922, 98% *ee* according to the manufacturer's specifications) was dissolved in 1.2 mL of acetone and 0.356 mL of iodomethane (2 M solution in *tert*-butyl methyl ether, 0.712 mmol, 0.99 eq) were added, and the resulting solution was stirred at room temperature for 17 h. After thorough removal of the solvent under reduced pressure the obtained residue was purified by preparative HPLC using a Phenomenex Synergi Fusion-RP C_{18} column (80-4, 250 × 21.2) with H_2O + 0.1% TFA as mobile phase at a flow rate of 15 mL min^{-1} to obtain 118 mg of *R*-**4** (57%, colourless oil) as TFA salt. To check for iodide, 4 mg of the obtained product was dissolved in 0.5 mL of H_2O and 0.1 mL of a $AgNO_3$ solution (0.1016 N) was added. No precipitate formed indicating that no residual iodide was present in the sample.

$[\alpha]_D^{23}$ = −2.6 (*c* = 0.32, MeOH);

^{1}H NMR (300 MHz, CD_3OD): δ 4.12 (m, 1H), 3.49–3.33 (m, 2H), 2.91 (s, 3H), 2.90 (s, 3H), 2.51 (d, *J* = 6.4, 2H), 2.10 (m, 1H), 1.93 (m, 1H).

^{13}C NMR (76 MHz, CD_3OD): δ 174.6, 67.7, 42.6, 42.3, 31.7, 25.8, 25.5.

HRMS: Calculated for $C_7H_{15}O_3S$, ($[M+H]^+$): 179.0736; found: 179.0735.

***S*-Gonyol (*S*-4)**

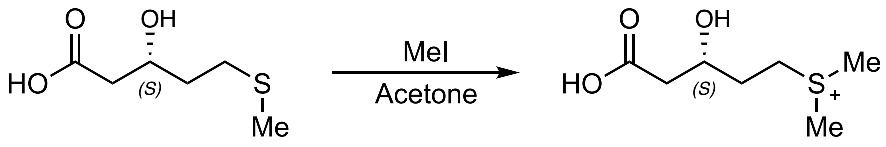

(*S*)-3-Hydroxy-5-(methylthio)pentanoic acid (111 mg, 0.676 mmol, 1 eq.) as obtained from Enamine Ltd. (Kiev, Ukraine, product EN300-1807923, 100% *ee* according to the manufacturer's specifications) was dissolved in 1.1 mL of acetone and 0.338 mL of iodomethane (2 M solution in *tert*-butyl methyl ether, 0.676 mmol, 1 eq) were added and the resulting solution stirred at room temperature for 17 h. After removal of the solvent under reduced pressure the obtained residue was purified by preparative HPLC using a Phenomenex Synergi Fusion-RP C_{18} column (80-4, 250 × 21.2) with H_2O + 0.1% TFA as mobile phase at a flow rate of 15 mL min^{-1} to obtain 29 mg of *S*-**4** (15%, colourless oil) as TFA salt. To check for iodide, 4 mg of the obtained product was dissolved in 0.5 mL of H_2O and 0.1 mL of a $AgNO_3$ solution (0.1016 N) was added. No precipitate formed indicating that no residual iodide was present in the sample.

$[\alpha]_D^{23}$ = +3.5 (*c* = 0.24, MeOH);

^{1}H NMR (300 MHz, CD_3OD): δ 4.12 (m, 1H), 3.49–3.33 (m, 2H), 2.91 (s, 3H), 2.90 (s, 3H), 2.51 (d, *J* = 6.4, 2H), 2.10 (m, 1H), 1.93 (m, 1H).

^{13}C NMR (76 MHz, CD_3OD): δ 174.8, 67.7, 42.7, 42.3, 31.7, 25.8, 25.5.

HRMS: Calculated for $C_7H_{15}O_3S$, ($[M+H]^+$): 179.0736; found: 179,0736

Carba-gonydiol (12, *anti*-2,3-dihydroxy-6-methylheptanoic acid)

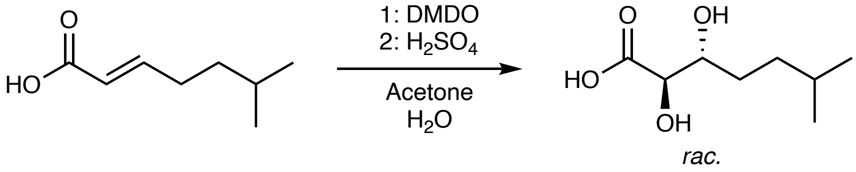

DMDO was prepared according to a known procedure[45] and obtained at a concentration of 74 mM. 18 mL of this DMDO solution (98.7 mg, 1.33 mmol, 2.1 eq) in acetone were added to (*E*)-6-methylhept-2-enoic acid (89 mg, 0.626 mmol, 1 eq., obtained from Enamine Ltd., Kiev, Ukraine, product EN300-7641056) in 4 mL acetone and the resulting solution stirred for 21 h at room temperature. Subsequently 20 mL of H_2SO_4 (0.1 M) were added followed by removal of the solvent under reduced pressure at 50 °C. The obtained residue was redissolved in 5 mL of H_2SO_4 (0.1 M) and extracted with EtOAc (3 ×). The organic phase was removed under reduced pressure followed by purification by preparative HPLC using a Phenomenex Synergi Fusion-RP C_{18} column (80-4, 250 × 21.2) with an elution gradient [solvent A: H_2O + 0.1% TFA, solvent B: CH_3CN 83%, 0% B for 5 min, from 0% to 100% B in 25 min; flow rate: 15 mL min^{-1}]. Through this 14.9 mg of **12** (14%) as racemate was obtained as white solid.

^{1}H NMR (300 MHz, CD_3CN): δ 4.04 (d, *J* = 4.6, 1H), 3.69 (m, 1H), 1.54 (m, 1H), 1.48 (m, 2H), 1.42–1.33 (m, 2H), 0.89 (d, *J* = 3.1, 3H), 0.87 (d, *J* = 3.1, 3H).

^{13}C NMR (76 MHz, CD_3CN): δ 174.0, 74.7, 73.8, 35.5, 30.7, 28.7, 23.0, 22.6.

HRMS: Calculated for $C_8H_{15}O_4$, ([M–H]$^-$): 175.0976; found: 175.0974.

***rac*-Trigonic acid (6)**

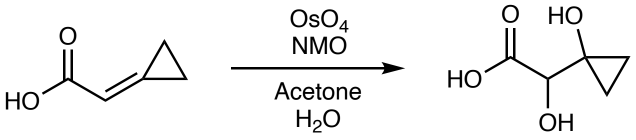

2-Cyclopropylideneacetic acid (397 mg, 4 mmol, 1 eq, obtained from Enamine Ltd., Kiev, Ukraine, product EN300-96997) was dissolved in a mixture of 3 mL acetone/H_2O (7:3), *N*-methylmorpholine-*N*-oxide (549.4 mg, 4.7 mmol, 1.2 eq.) and 80 µL of an OsO_4 solution (4% in H_2O, 3.2 mg, 12.6 µmol, 0.3 mol-%) were added and the solution stirred for 20 h at room temperature. After quenching by addition of 520 mg of Na_2SO_3 (dissolved in 3 mL of H_2O) the aqueous solution was acidified with 15 mL HCl and extracted with EtOAc (4 ×) followed by removal of the solvent under reduced pressure. Purification via preparative HPLC using a Phenomenex Synergi Fusion-RP C_{18} column (80-4, 250 × 21.2) with an elution gradient [solvent A: H_2O + 0.1% TFA, solvent B: CH_3CN 83%, 0% B for 5 min, from 0% to 100% B in 25 min; flow rate: 15 mL min^{-1}] yielded 196 mg of racemic **6** (37%, white solid).

^{1}H NMR (300 MHz, CD_3CN): δ 3.67 (s, 1H), 0.86–0.60 (m, 4H).

^{13}C NMR (76 MHz, CD_3CN): δ 174.1, 76.1, 57.7, 12.4, 12.2.

HRMS: Calculated for $C_5H_7O_4$, ($[M–H]^-$): 131.0350; found: 131.0348.

Ethyl *R*-2-hydroxy-2-(1-hydroxycyclopropyl)acetate

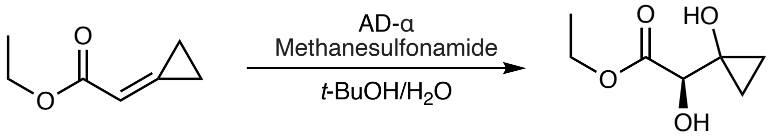

AD-mix-α (609 mg) was dissolved in 5 mL of a mixture of tert-butanol and H_2O (1:1), 41.5 mg of methansulfonamide were added and the resulting biphasic solution cooled to 0 °C. Subsequently, ethyl 2-cyclopropylideneacetate (53.5 mg, 0.424 mmol, obtained from Enamine Ltd., Kiev, Ukraine, product EN300-105471) was added, and the mixture was stirred for 16 h at 0 °C. The reaction was quenched by addition of 262 mg of Na_2SO_3 and stirring for 30 min while warming to room temperature followed by extraction with EtOAc (3 ×). The obtained organic phase was washed with brine (2 ×), dried over Na_2SO_4 and the solvent removed under reduced pressure. Purification via preparative HPLC using a Phenomenex Synergi Fusion-RP C_{18} column (80-4, 250 × 21.2) with an elution gradient [solvent A: H_2O + 0.1% TFA, solvent B: CH_3CN 83%, 0% B for 5 min, from 0% to 100% B in 25 min; flow rate: 15 mL min^{-1}] yielded 9.2 mg (14%, colourless oil) of ethyl *R*-2-hydroxy-2-(1-hydroxycyclopropyl)acetate with an *ee* of 82% as determined after hydrolysis (see below).

$[\alpha]_D^{20}$ = −44.3 (c = 0.38, MeOH);

^{1}H NMR (300 MHz, CD_3CN): δ 4.20 (q, J = 7.3, 2H), 3.65 (s, 1H), 2.17 (br s, 2H), 1.26 (t, J = 7.3, 3H), 0.84–0.59 (m, 4H).

^{13}C NMR (76 MHz, CD_3CN): δ 174.8, 67.7, 42.7, 42.3, 31.7, 25.8, 25.5.

HRMS: Calculated for $C_7H_{13}O_4$, ($[M+H]^+$): 161.0814; found: 161.0813.

***R*-Trigonic acid (*R*-6)**

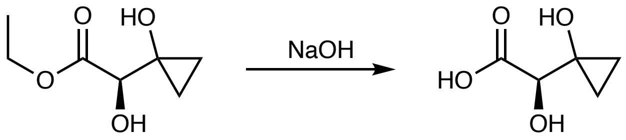

Ethyl (*R*)-2-hydroxy-2-(1-hydroxycyclopropyl)acetate (27.1 mg, 0.169 mmol) was dissolved in 2 mL NaOH (0.2 M) and stirred for 4 h at room temperature. The reaction mixture was washed with EtOAc and the remaining aqueous phase acidified by addition of 10 mL of HCl (1 M) followed by extraction with EtOAc (4 ×) and drying over Na_2SO_4. Purification by preparative HPLC using a Phenomenex Synergi Fusion-RP C_{18} column (80-4, 250 × 21.2) with an elution gradient [solvent A: H_2O + 0.1% TFA, solvent B: CH_3CN 83%, 0% B for 5 min, from 0% to 100% B in 25 min; flow rate: 15 mL min^{-1}] yielded 2.8 mg (13%, white solid) of *R*-**6**. The enantiomeric excess of the *R*-enantiomer over the *S*-enantiomer was determined by means of chiral LC-HRMS to be 82%.

[α]$_D^{20}$ = −46.5 (c = 0.47, MeOH);

^{1}H NMR: see above.

^{13}C NMR: see above.

HRMS: Calculated for $C_5H_7O_4$, ($[M–H]^-$): 131.0350; found: 131.0350.

Distinct crystallization parameters

BurG (holo) (PDB ID 7PCC): 0.1 M Bicine (pH 9.0), 20% PEG 8K; 2 mM NAD^+, 5 mM $MgCl_2$

BurG (apo) (PDB ID 7PCE): 0.1 M Na/K phosphate (pH 6.2), 0.2 M NaCl, 50% PEG 200

BurG:7 (PDB ID 7PCG): 0.1 M Tris (pH 8.5), 20% PEG 10K; 2 mM NAD^+, 5 mM $MgCl_2$, 2 mM **7**

BurG:8 (PDB ID 7PCI): 0.05 M imidazole (pH 8.0), 20% PEG 6K, 2 mM NAD^+, 5 mM $MgCl_2$, 2 mM **8**

BurG:11 (PDB ID 7PCL): 0.1 M Tris (pH 8.5), 20% PEG 10KJ; 2 mM NADH, 5 mM $MgCl_2$, 2 mM **10** (obtained from Enamine Ltd., Kiev, Ukraine, product EN300-124480)

BurG:13 (PDB ID 7PCM): 0.1 M Tris (pH 8.5), 0.8 M LiCl, 8% PEG 4K; 2 mM NAD^+, 5 mM $MgCl_2$, 2 mM **12**

BurG:14 / BurG:6 (PDB ID 7PCN): 0.1 M imidazole (pH 8.0), 10% PEG 8K, 2 mM NAD^+, 5 mM $MgCl_2$, 2 mM **5**

BurG-E232Q:12 (PDB ID 7PCO): 0.1 M Na acetate, 0.05 M Mg acetate, 10% PEG 8K, 2 mM NAD^+, 5 mM $MgCl_2$, 2 mM **12**

BurG-E232Q:15 (PDB ID 7PCT): 0.1 M Tris (pH 8.5), 0.2 M Na acetate, 16% PEG 4K.

Nucleotide sequence of synthetic *burG E232Q*

Underlined parts indicate the *burG* sequence with the mutated position highlighted in yellow:

GGTGCTAGCAACGATTTAATCTACCAAGATGAGCACGCTTCGTTGCAGCCTCTGGAAGGTCGTACTGTGGCAGTG
ATTGGATACGGTATTCAAGGACGCGCCTTTGCGGCCAACCTGCGCGATTCAGGAGTGGCAGTACGTGTAGGCAAC
ATTGACGACCGTTATTTTGAATTGGCGCGCGCAGAAGGCCATCGTGTAACGAACATTGCAGAAGCGGTGGCCCAT
GCCGATATCGTGTTATTGTTGATTCCTGACGAGGCGCACGGTGCTGTCTTCGACGTAGATATTGCGCCGAACTTG
CGTGATGGGGCACTGTTATGTGTGGCACACGGCCATAGTCTTGTCCAGGGAGACGTTCGTCCCTTGCCCGGTCGT
GATCTGGCGATGCTGGCCCCCCGTATGTACGGTGACCCCATTCGTCGCTACTATCTTGCAGGCCAGGGGGCCCCG
GCATATTTCGACATCGTAGCCGACCACACTGGCCGCGCGCGTGATCGCGTATTAGCGATTGCCCGTGCAGTAGGA
TTTACTCGCGCAGGGGTTATGGCTCTTGGTTACCGCCAGGAGACATTTCTGGATTTGTTTCAAGAGCAGTTCTTA
GCGCCAGCTTTAGTCGACTTGGTAGAGACTGGCTTTCAAGTATTAGTGGAGCGCGGCTTTAACCCCAAGGCCGCG
TTATTGGAAGTCTACGGTTCGGGTCAGATGGGAAAAATGATGTTGGATGGCGCGGATATCGGACTGGATGAAGTG
GTGGCATTACAGGGCTCACCCACGTGCCAGGTGGGATATCATCGCTGGCGTGGACGCACATTGCCGACAGCCGTG
CGCGAGCTTGCAGCCCGCGTTCTTGATCAAATCGAGGGTGGCGATTTCTCCGCCTACTTGAAGGAGCAAGCGTCT
AATGATTATGCCAGTCTGGACGACGCGCGTCGTGCGGCTCTTAAACGTCCCCTGAATGTGGCGCACGCACAAGTG
CGTGCTGCGTTCCGTTTCCCAACAGAGGCCGCTGGCGGTCTTTATCAGGCTGCGCAGGCACCTGCTGATGTCGAA
CCAGAGGCCGCACGTTGAAAGCTTACC

Supplementary Figures

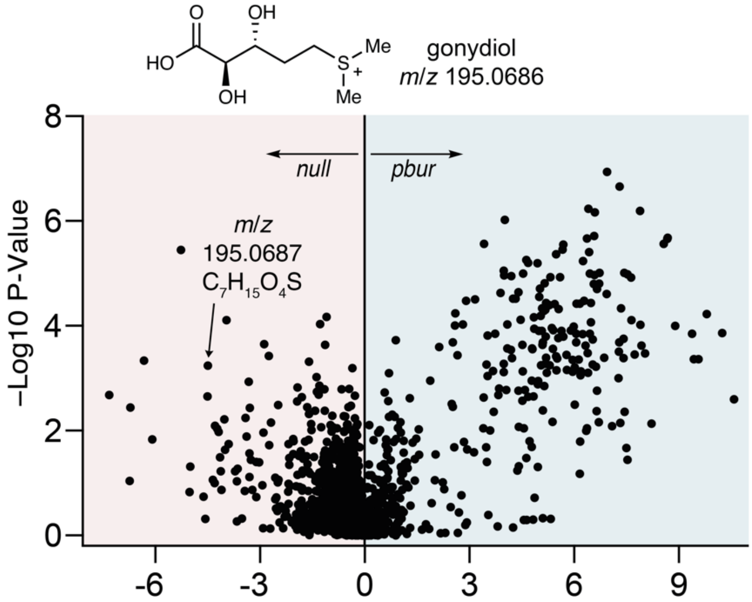

Supplementary Figure 1 | Comparative metabolomics analysis. Analysis of *B. thailandensis Pbur* vs. *B. thailandensis Pbur burG::Kan*; full, non-filtered view of metabolites in cell extracts (biological triplicates, n = 3) shows the upregulation of a compound with an *m*/*z* value of 195.0687 in the extract of *B. thailandensis Pbur burG::Kan* which corresponds to the biosynthetic intermediate gonydiol (**5;** *m*/*z* calculated: 195.0686). Accumulation of this compound in the gene-deletion mutant *B. thailandensis Pbur burG::Kan* indicates this compound as possible substrate for the corresponding enzyme BurG.

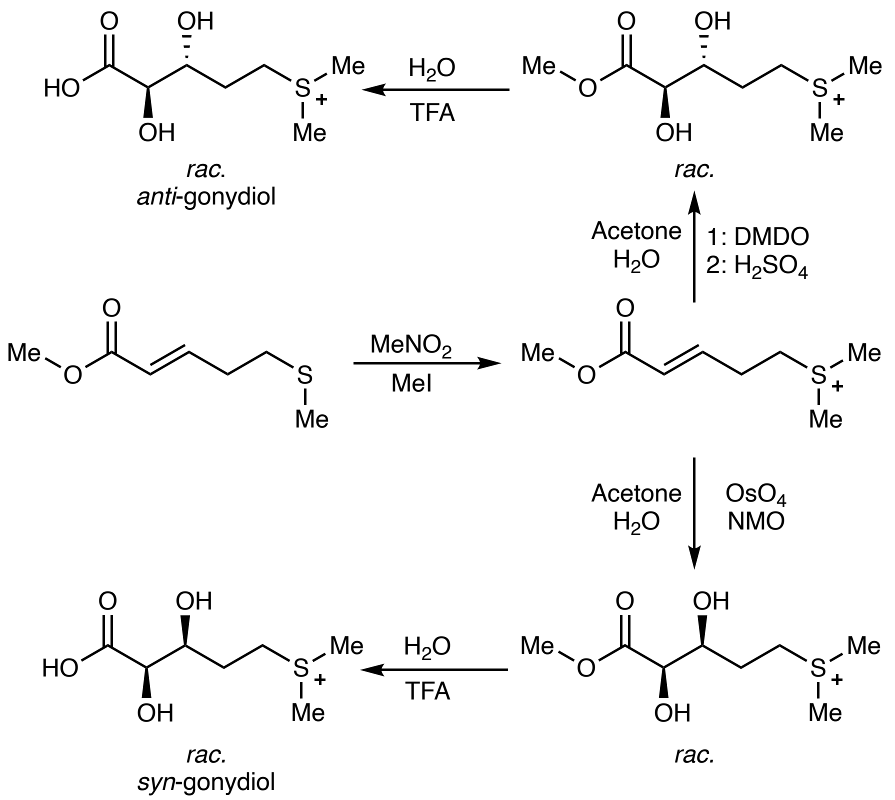

Supplementary Figure 2 | Synthesis of gonydiol. Route to synthetic *syn*- and *anti*-gonydiol (**5**) as racemic trifluoroacetate salts. TFA: trifluoracetic acid; NMO: *N*-methylmorpholine *N*-oxide; DMDO: dimethyldioxirane.

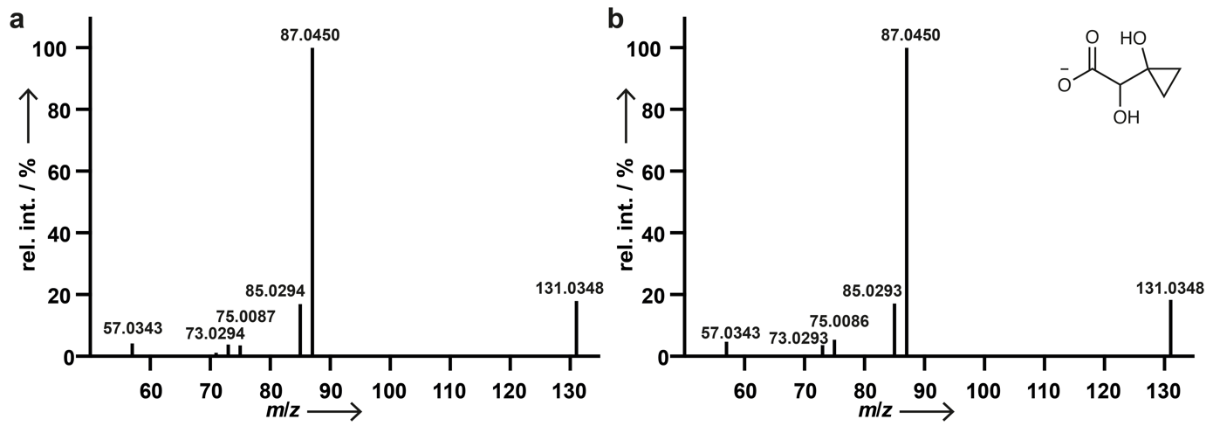

Supplementary Figure 3 | HR-MS^2 spectra of trigonic acid. Spectrum of **a**, enzymatically formed and **b**, synthetic trigonic acid acid (**6**). Spectra obtained with a normalized collision energy of 20 % in negative ion mode.

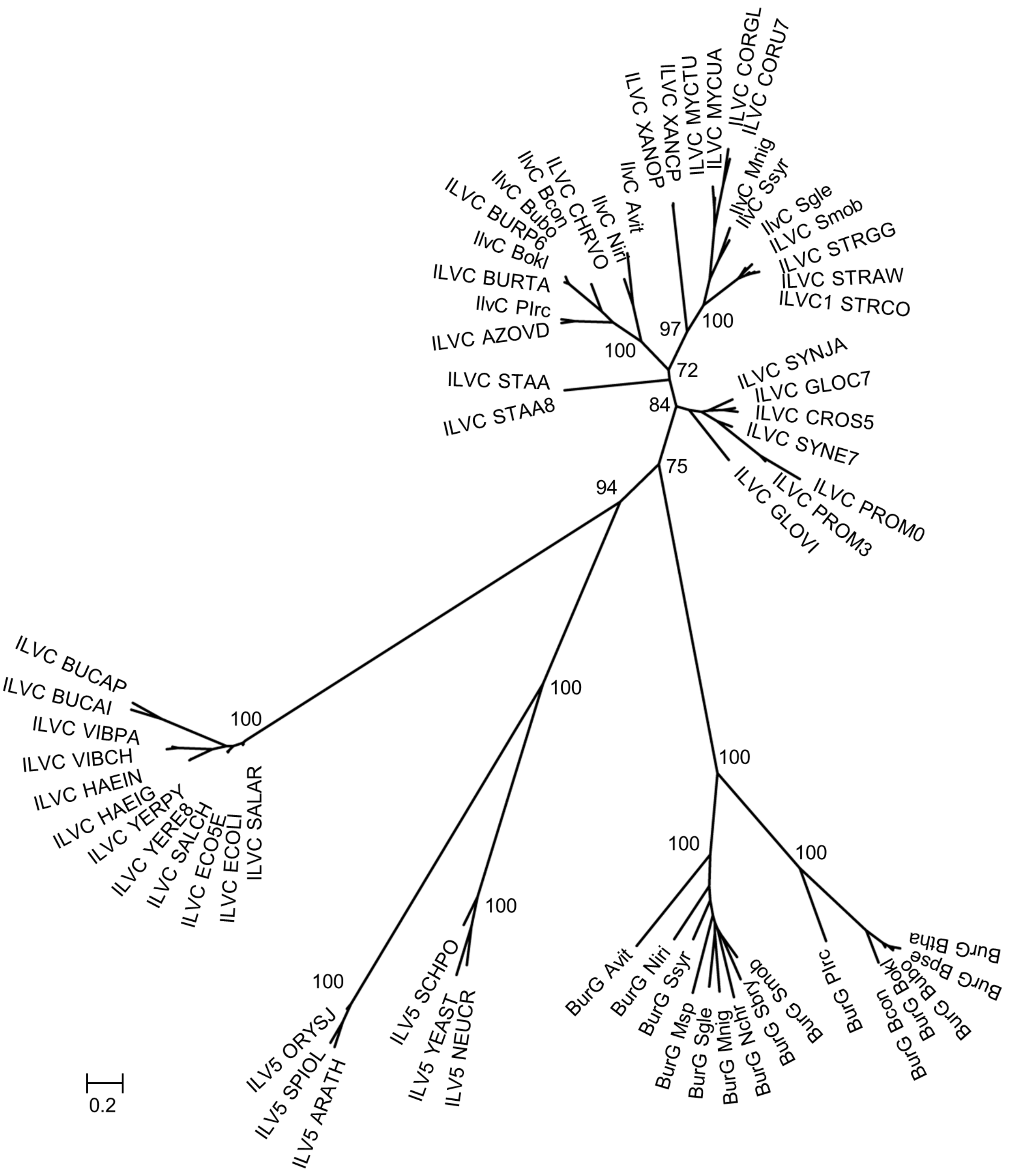

Supplementary Figure 4 | Extended phylogenetic tree. Phylogenetic analysis of amino acid sequences of BurG and other ketol-acid reductoisomerases (KARI, see Supplementary Table 6). The numbers at the nodes indicate the ultrafast bootstrap score (1,000 replicates, shown value: %) for reliability of the different groups. The scale bar shows amino acid substitutions per site.

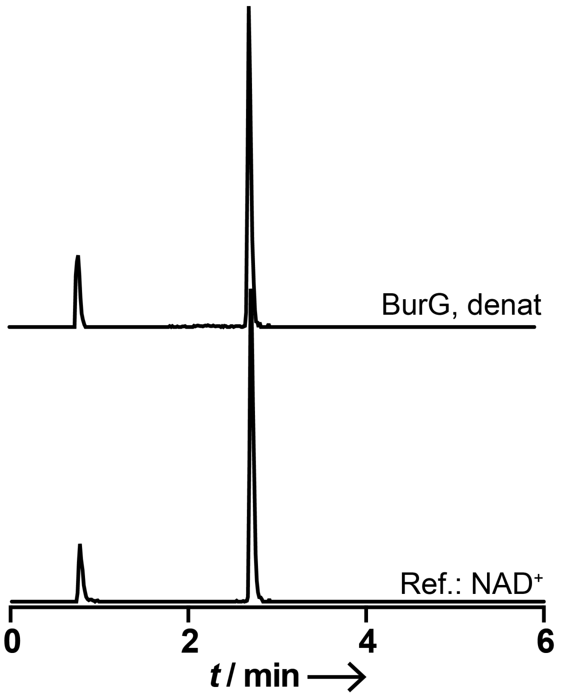

Supplementary Figure 5 | Detection of NAD$^+$ in denatured preparations of BurG. HR-LCMS extracted ion monitoring of NAD$^+$ (*m*/*z* of 664.1164 in negative ion mode); top: BurG preparation, bottom: commercial NAD$^+$ standard. No ions corresponding to either NADH, NADP$^+$ or NADPH were detected in positive or negative ion mode in the BurG preparation.

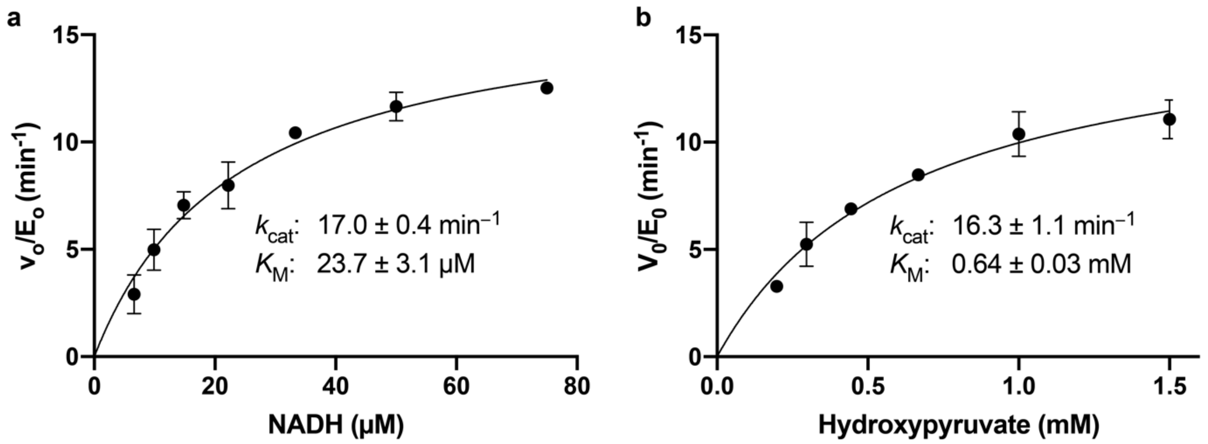

Supplementary Figure 6 | Michaelis Menten kinetics of BurG mediated reductions with hydroxypyruvate (8) and NADH as substrates. Error bars represent the standard deviation of the mean from biological duplicates (n = 2). **a,** Parameters determined for NADH with **8** at a fixed concentration (2 mM). **b,** Parameters determined for **8** with NADH at a fixed concentration (100 μM).

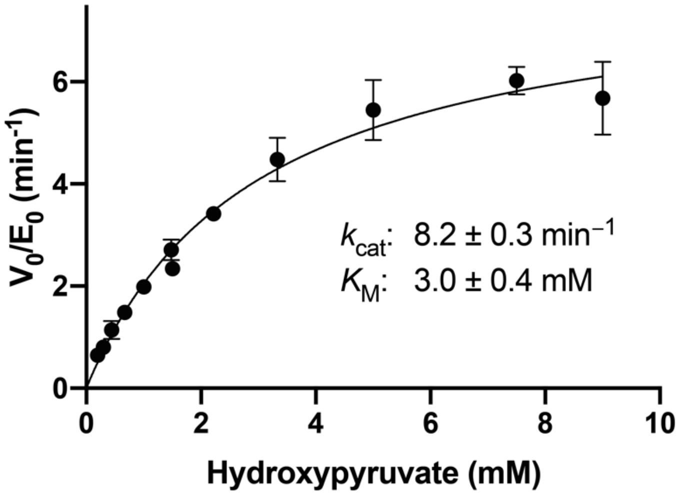

Supplementary Figure 7 | Michaelis Menten kinetics of BurG E232Q mediated reductions with hydroxypyruvate (8) and NADH as substrates. Error bars represent the standard deviation of the mean from technical duplicates (n = 2). Parameters determined for **8** with NADH at a fixed concentration (100 μM).

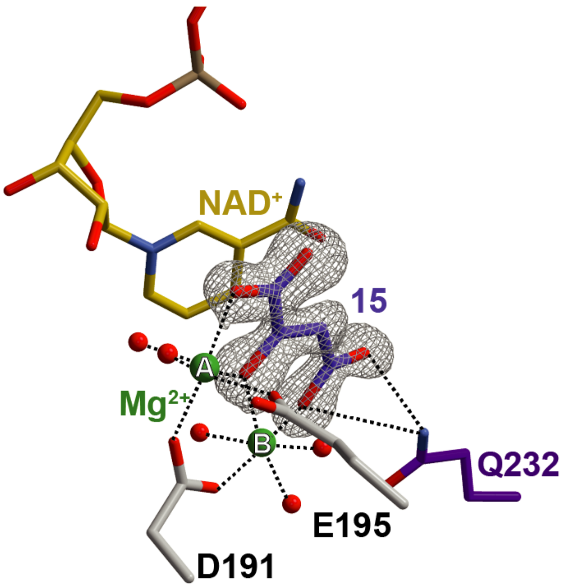

Supplementary Figure 8 | Complex structure of mutant BurG E232Q with bound enol-oxaloacetate (15) (PDB ID: 7PCT). The F_O-F_C electron density map for **15** bound to the Mg-atoms is shown as grey mesh contoured to 3σ. The ligand has been omitted for phasing. The introduced mutant Q232 is located on subunit B and marked in dark pink.

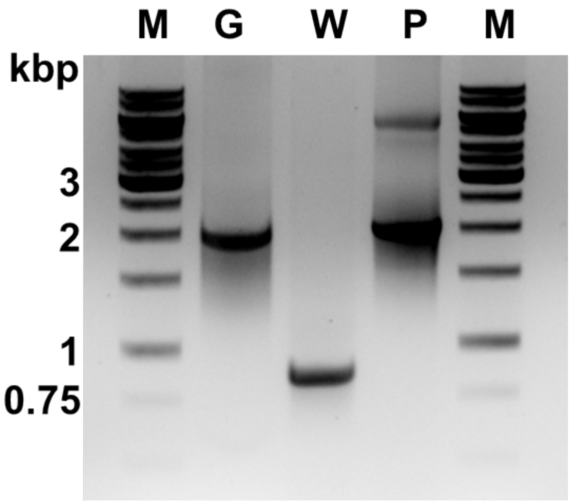

Supplementary Figure 9 | Verification of mutant strain. Agarose gel analysis of PCR products obtained with the primer pair Isomerase fw2 and Isomerase rv2 from: genomic DNA of *B. thailandensis Pbur burG*::*Kan* (lane G), genomic DNA of wild-type (lane W, expected size 839 bp) as a negative control, and pGEM-*burG*::*Kan* (lane P, expected size 1,871 bp) as a positive control. M: size marker.

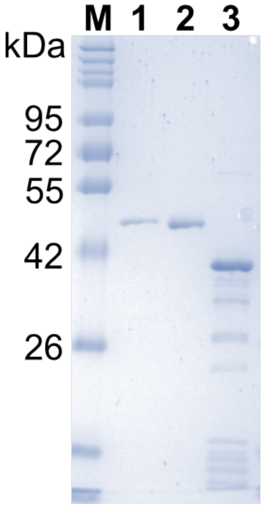

Supplementary Figure 10 | His$_6$-tagged enzyme preparations. Representative SDS-PAGE analysis of purified His$_6$-BurG (lane 1, 40.6 kDa), His$_6$-BurG E232Q (lane 2, 40.6 kDa), and His$_6$-BurC (lane 3, 39.9 kDa). M: size marker.

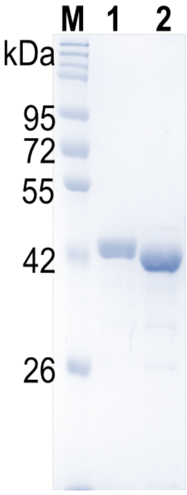

Supplementary Figure 11 | Tag-free enzyme preparations. Representative SDS-PAGE analysis of purified BurG (lane 1, 38.7 kDa) and BurG E232Q (lane 2, 38.7 kDa) after tag-cleavage by thrombin. M: size marker.

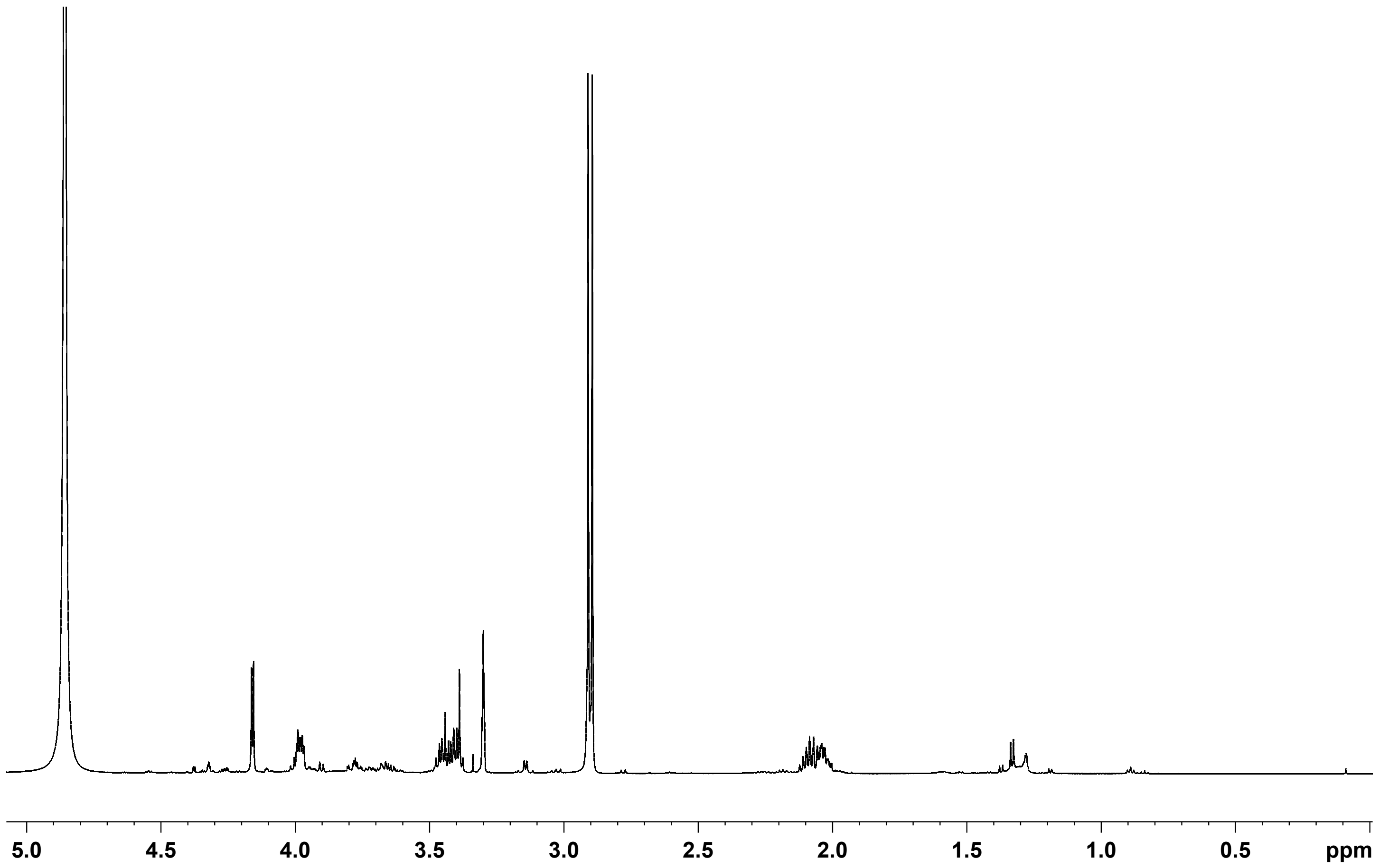

Supplementary Figure 12 | ^{1}H NMR spectrum of natural gonydiol (**5**; CD_3OD).

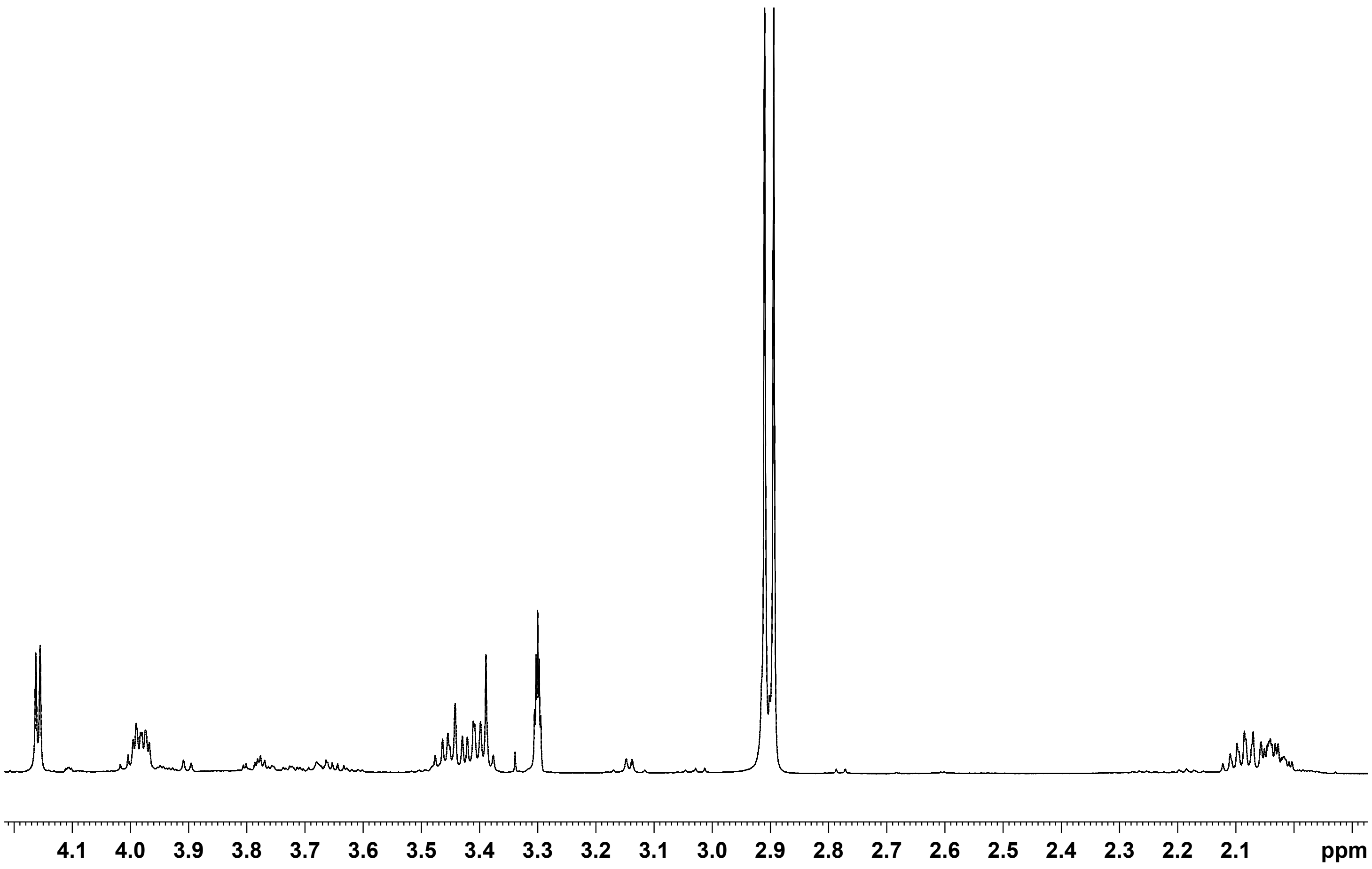

Supplementary Figure 13 | Zoom into ^{1}H NMR spectrum of natural gonydiol (**5**; CD_3OD).

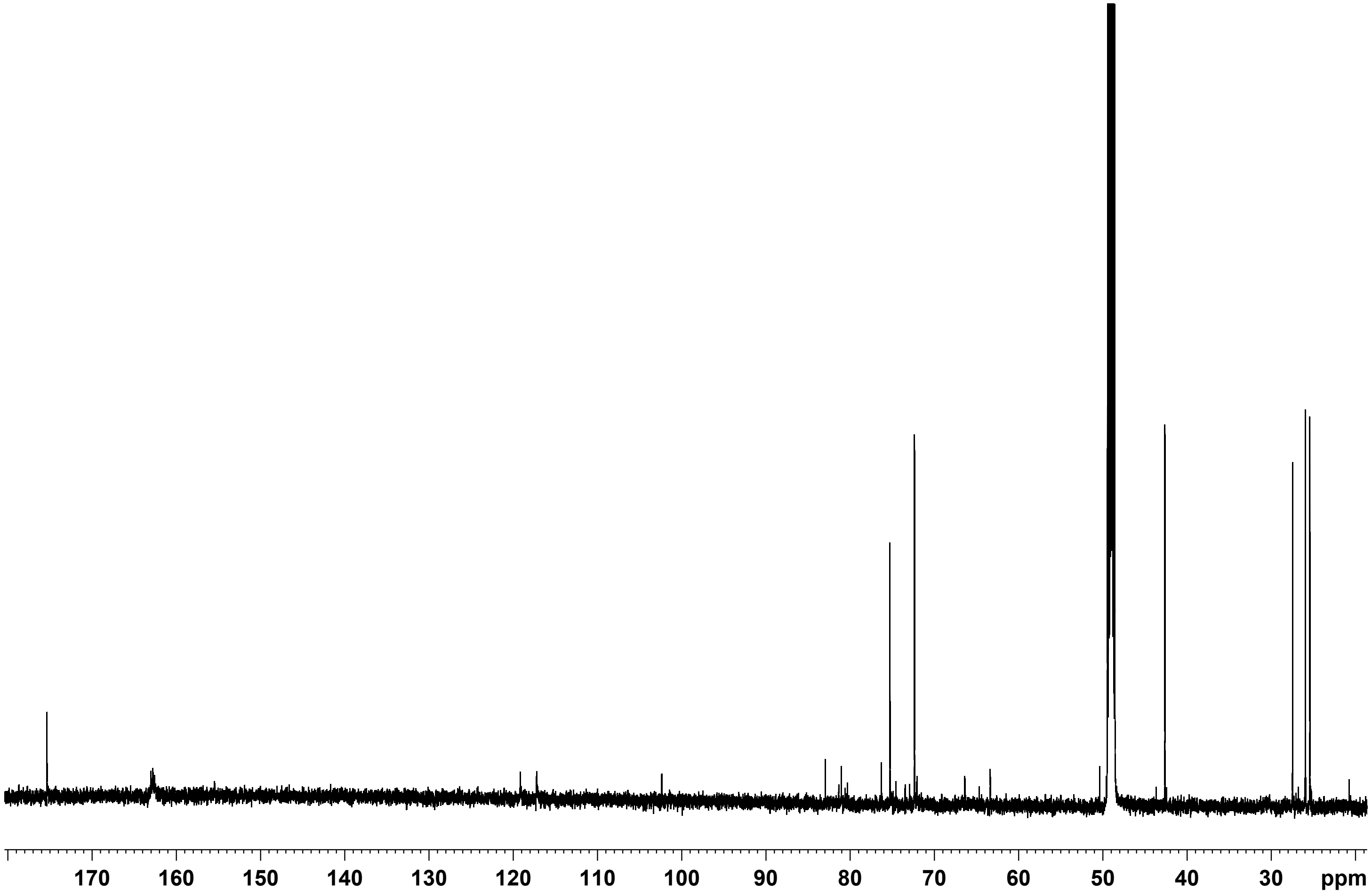

Supplementary Figure 14 | ^{13}C NMR spectrum of natural gonydiol (**5**; CD_3OD).

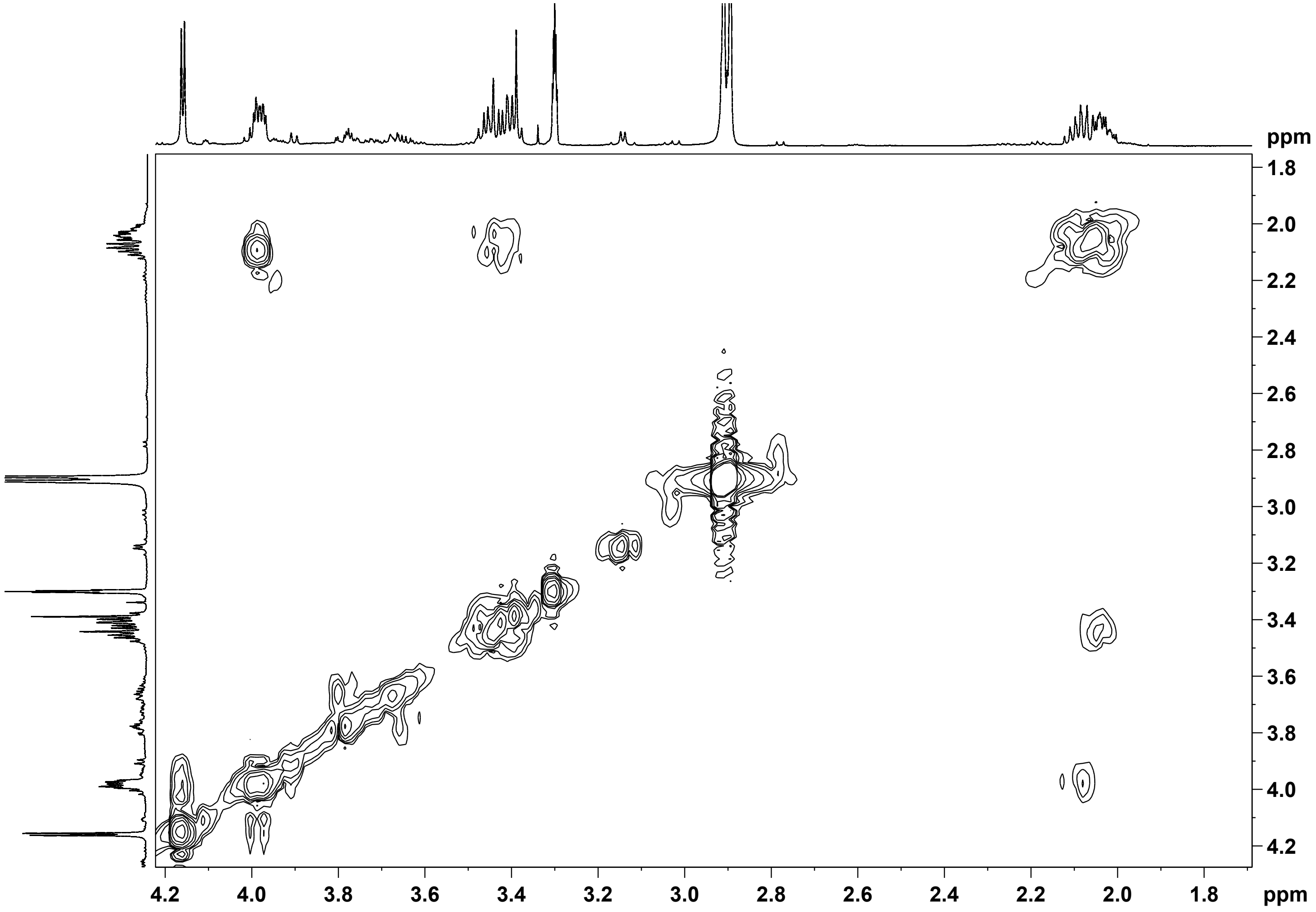

Supplementary Figure 15 | COSY spectrum of natural gonydiol (**5**; CD_3OD).

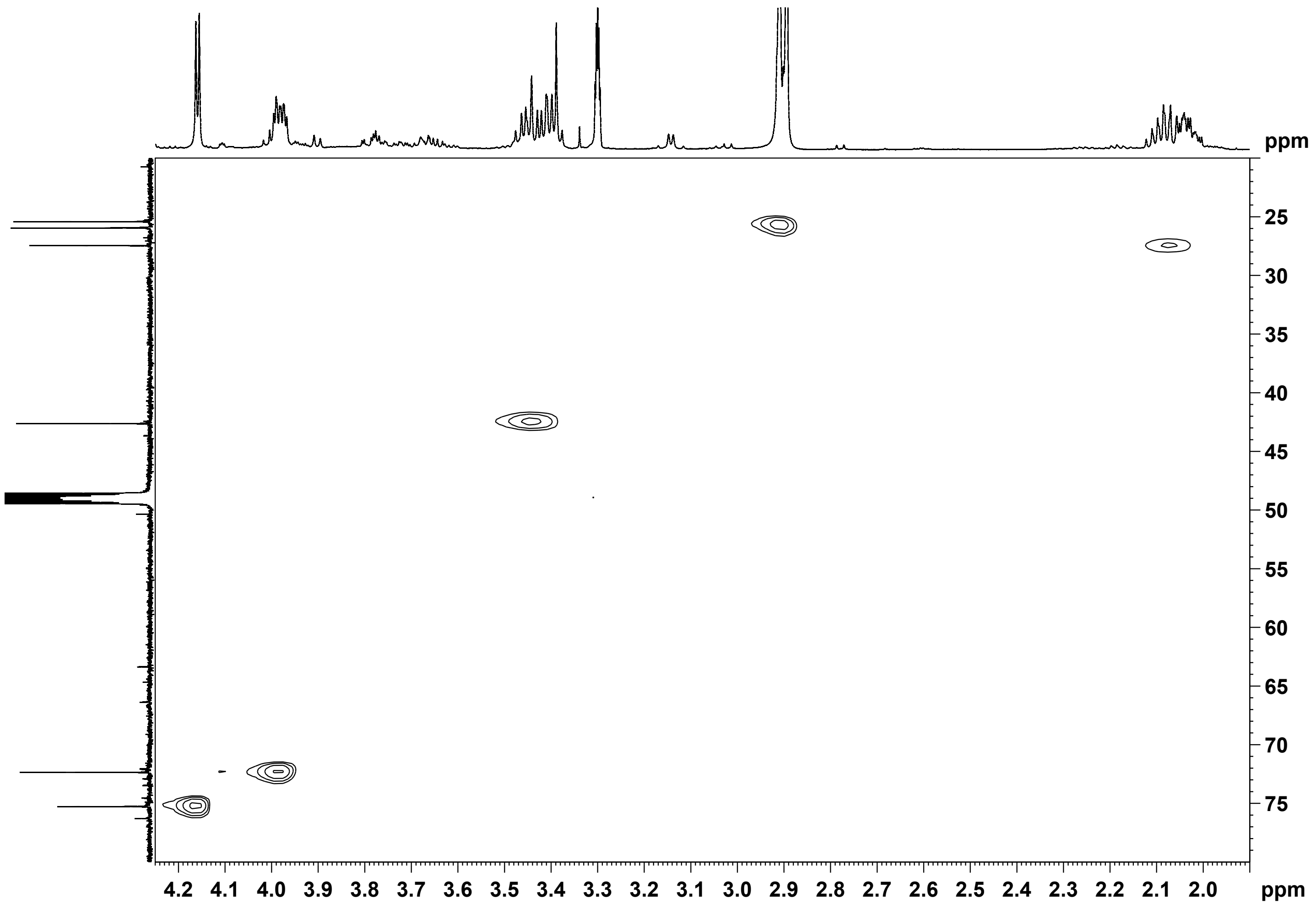

Supplementary Figure 16 | HSQC spectrum of natural gonydiol (**5**; CD_3OD).

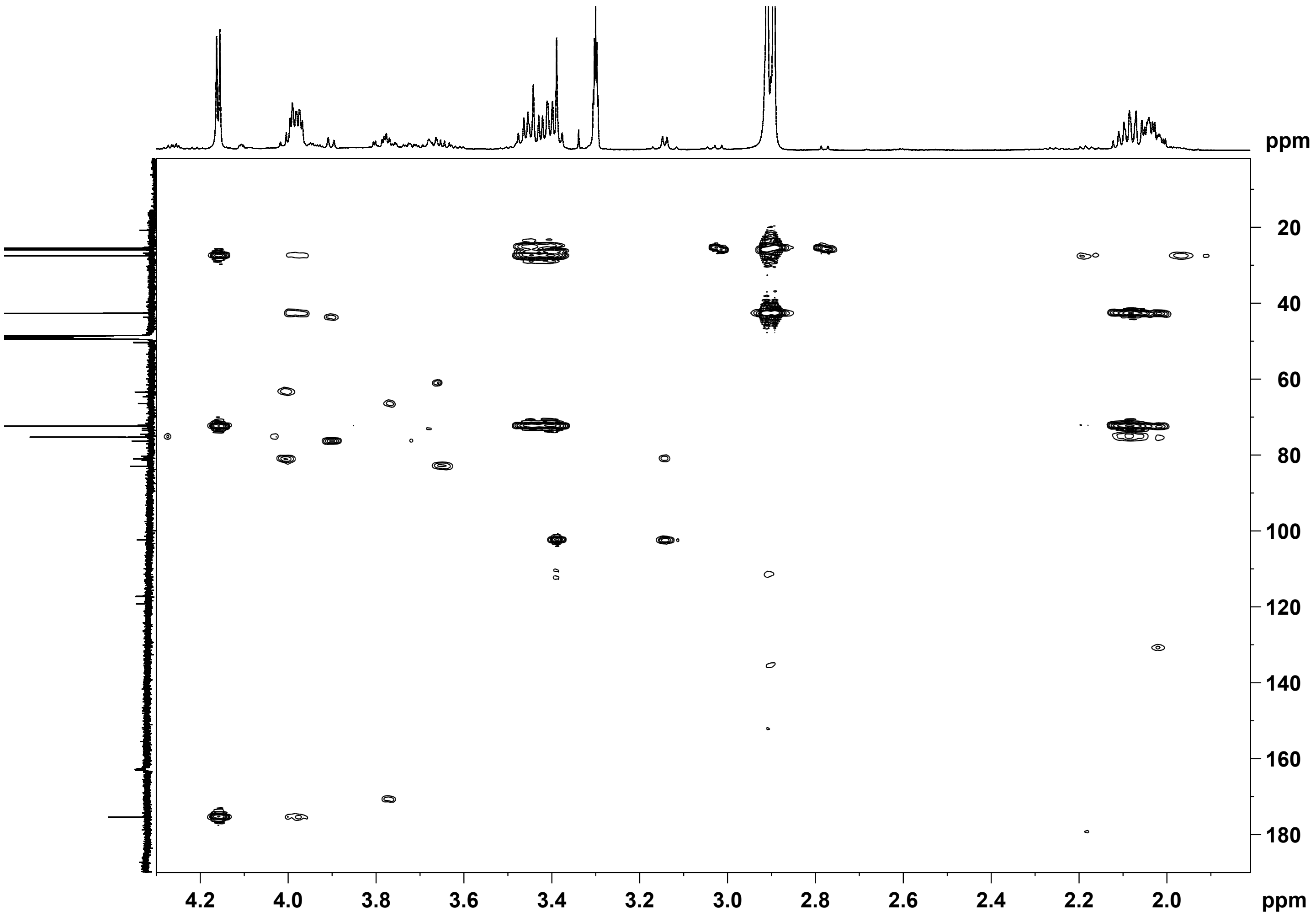

Supplementary Figure 17 | HMBC spectrum of natural gonydiol (**5**; CD_3OD).

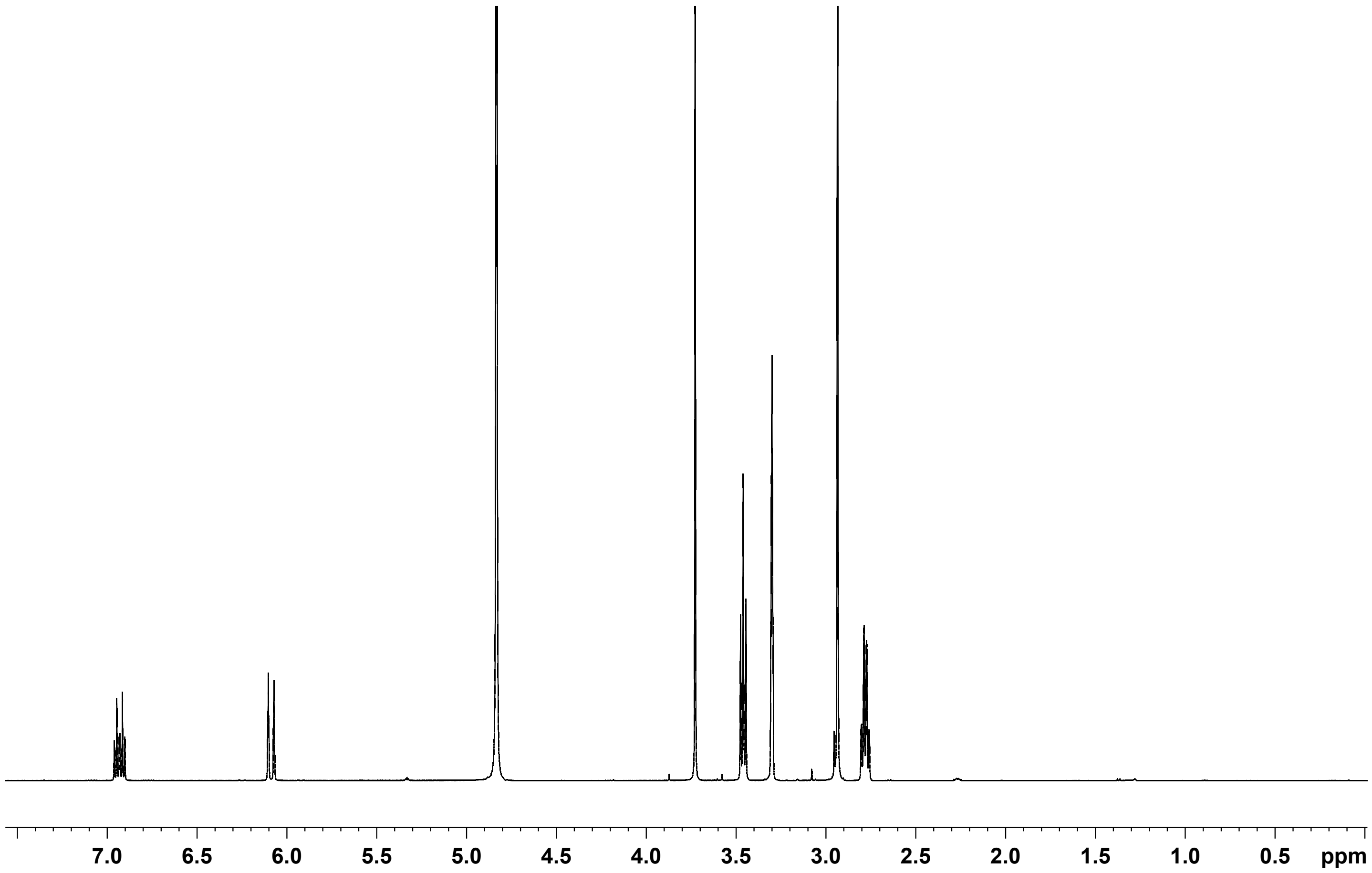

Supplementary Figure 18 | ^{1}H NMR spectrum of methyl (*E*)-5-dimethylsulfonio-pent-2-enoate (CD_3OD).

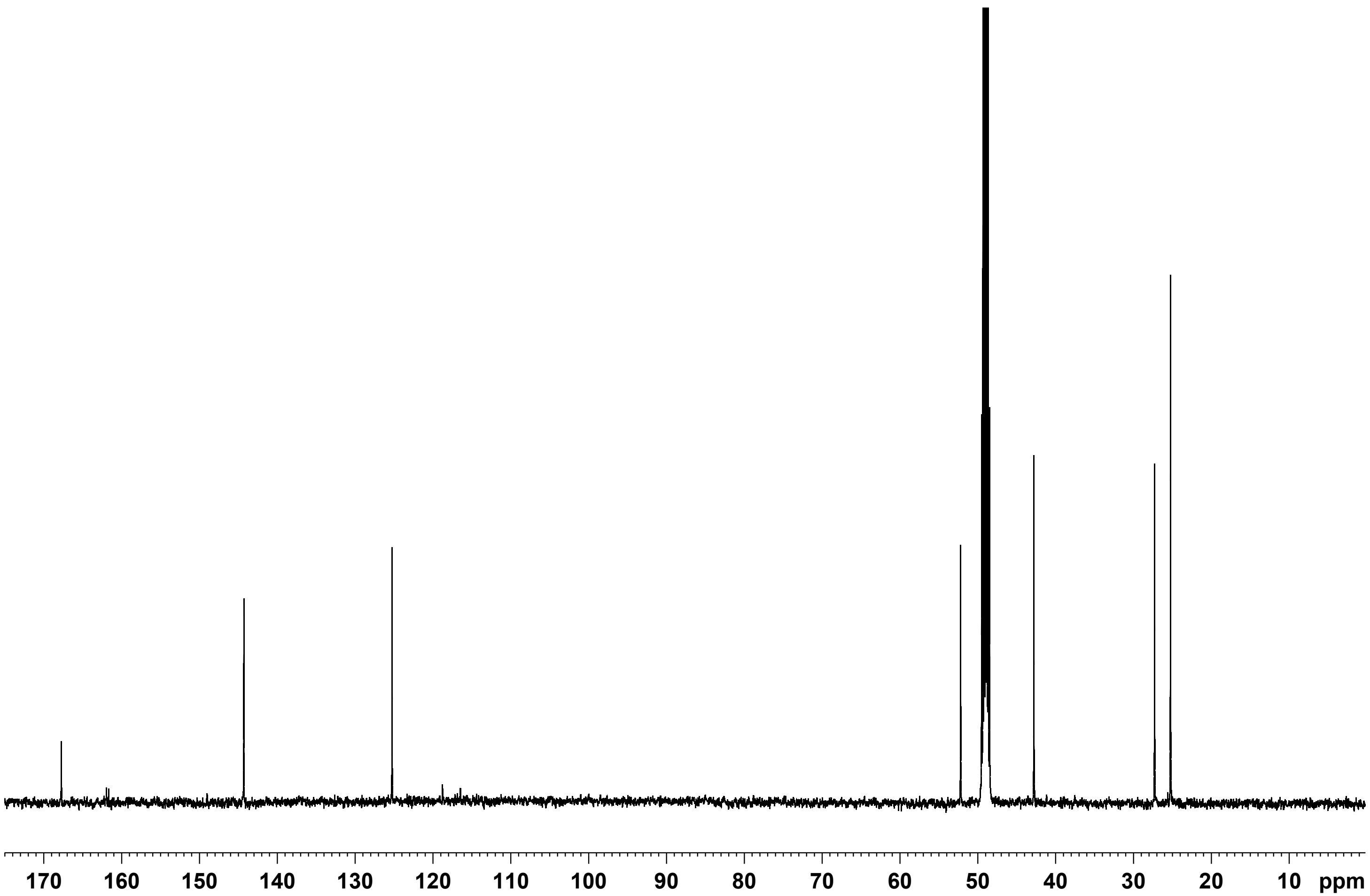

Supplementary Figure 19 | ^{13}C NMR spectrum of methyl (*E*)-5-dimethylsulfonio-pent-2-enoate (CD_3OD).

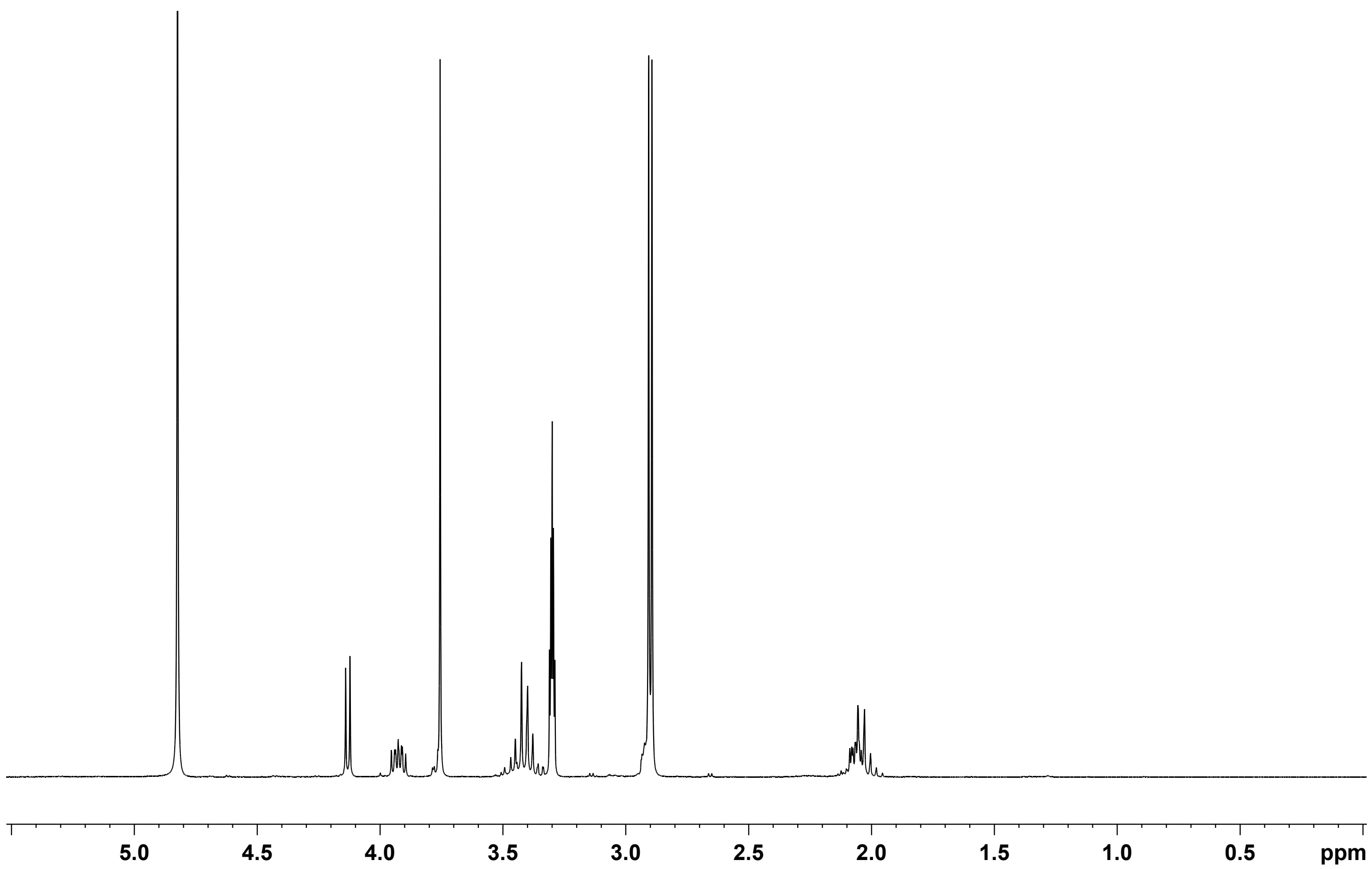

Supplementary Figure 20 | ^{1}H NMR spectrum of methyl *anti*-2,3-dihydroxy-5-dimethylsulfonio-pentanoate (CD_3OD).

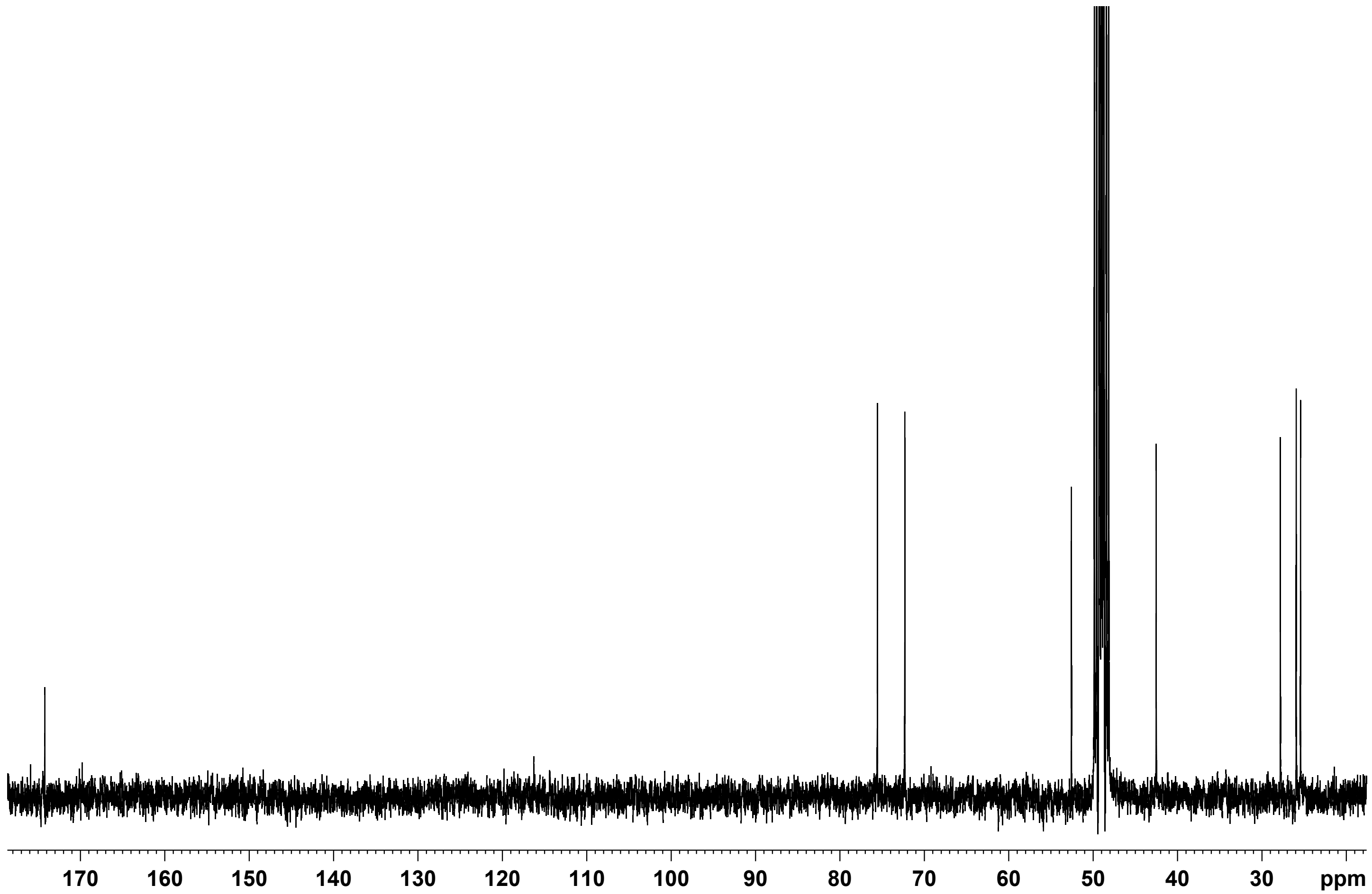

Supplementary Figure 21 | ^{13}C NMR spectrum of methyl *anti*-2,3-dihydroxy-5-dimethylsulfonio-pentanoate (CD_3OD).

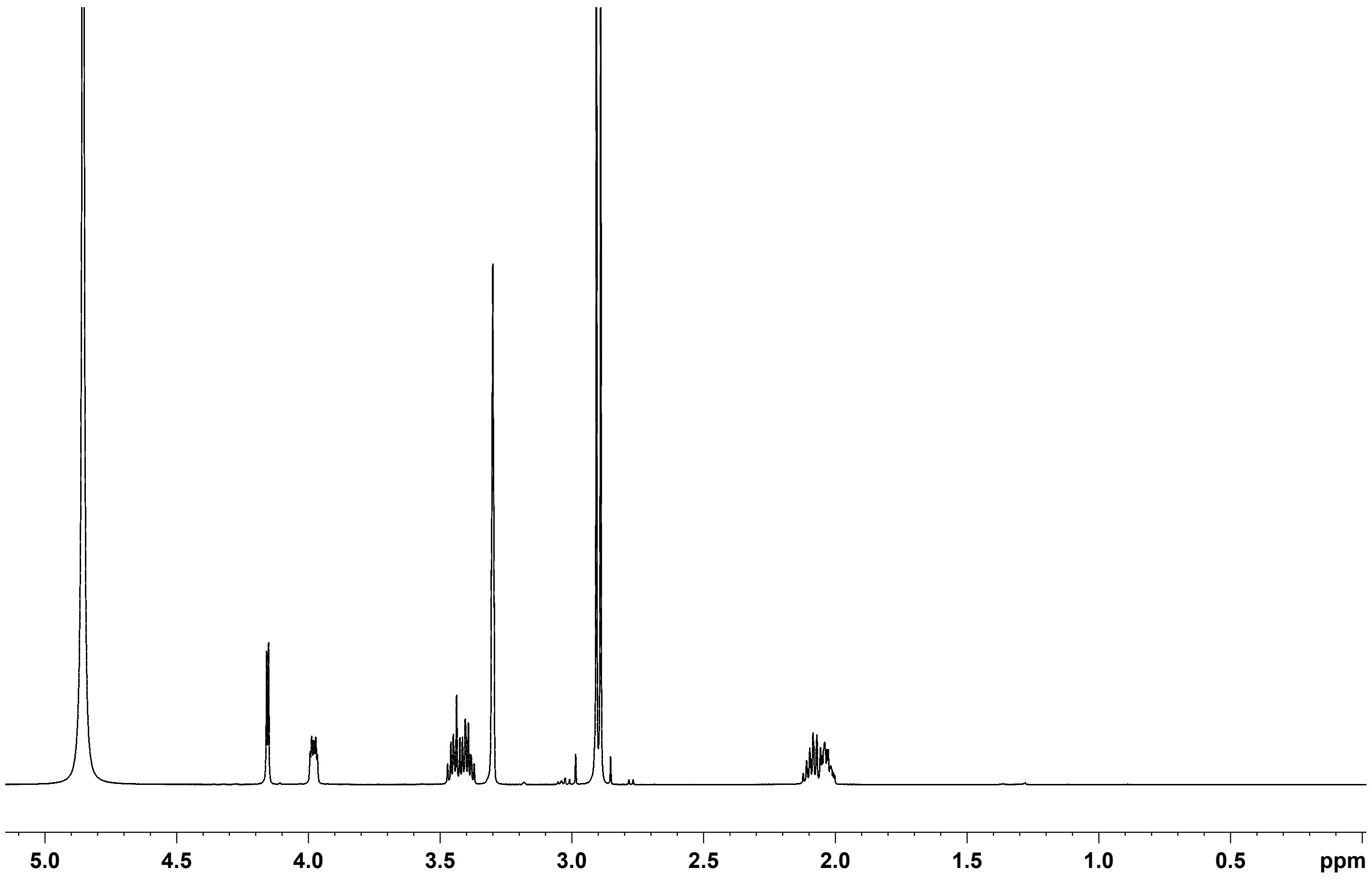

Supplementary Figure 22 | ^{1}H NMR spectrum of *anti*-gonydiol (*anti*-**5**; CD_3OD).

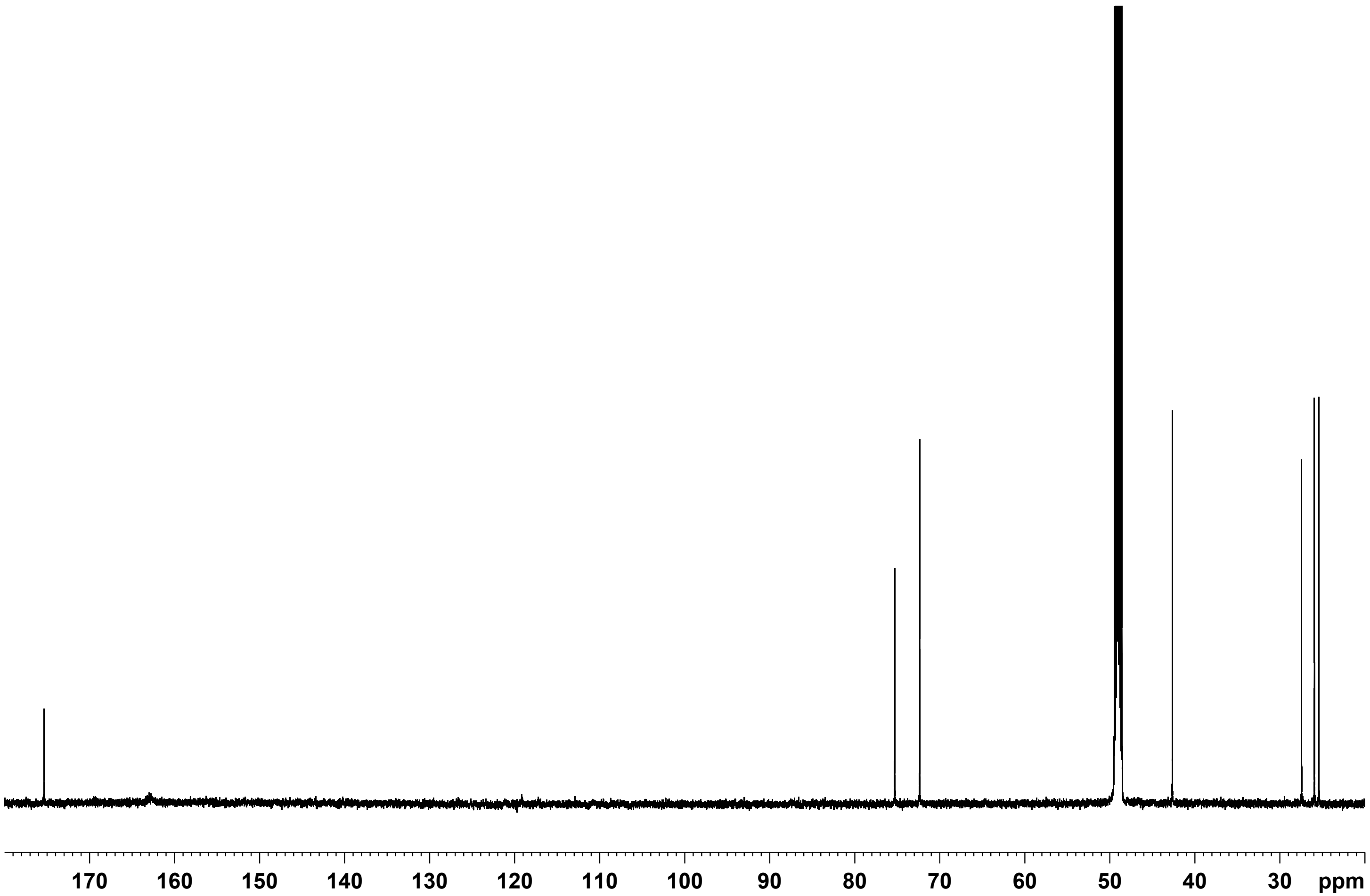

Supplementary Figure 23 | ^{13}C NMR spectrum of *anti*-gonydiol (*anti*-**5**; CD_3OD).

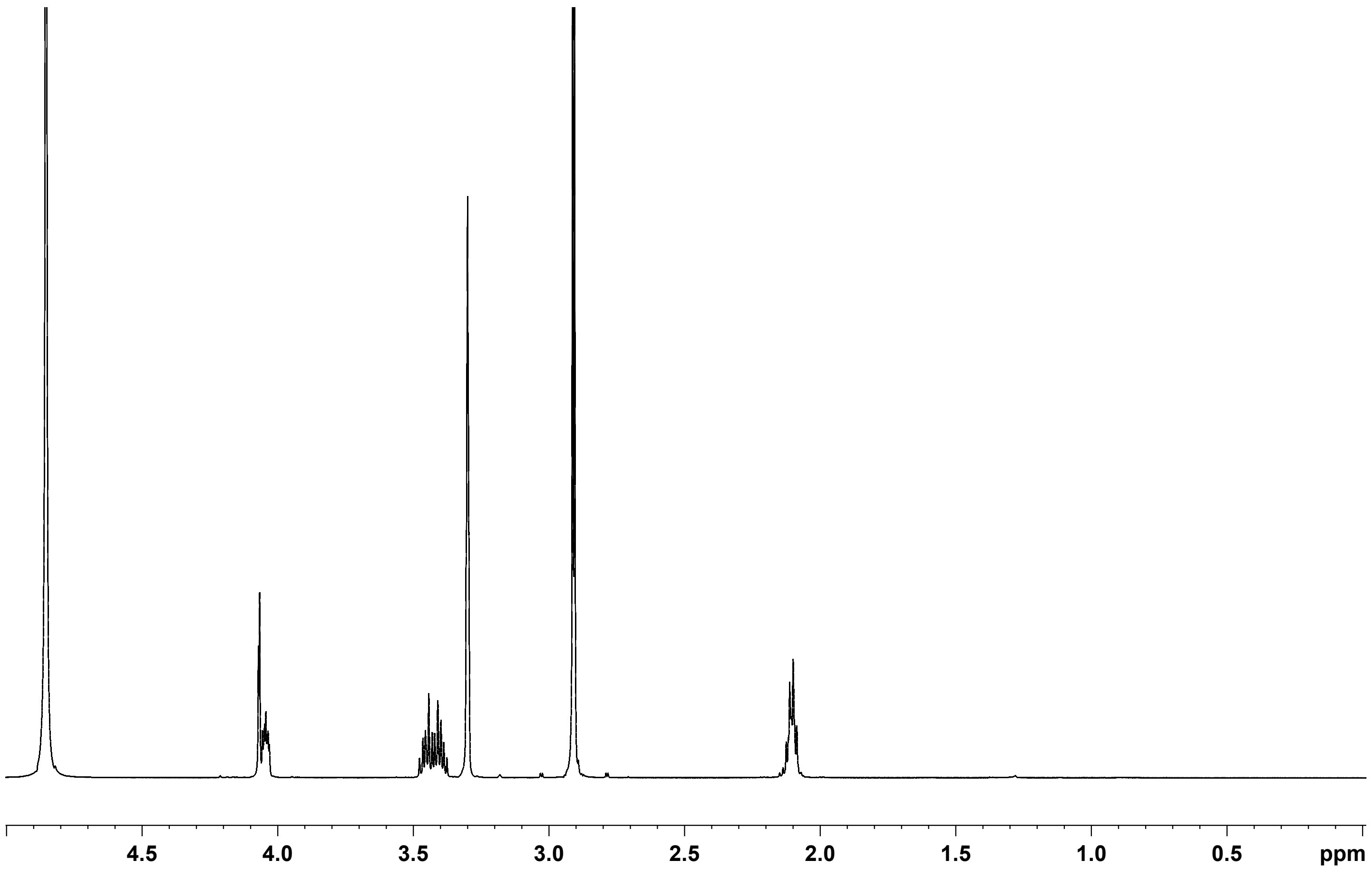

Supplementary Figure 24 | ^{1}H NMR spectrum of *syn*-gonydiol (*syn*-**5**; CD_3OD).

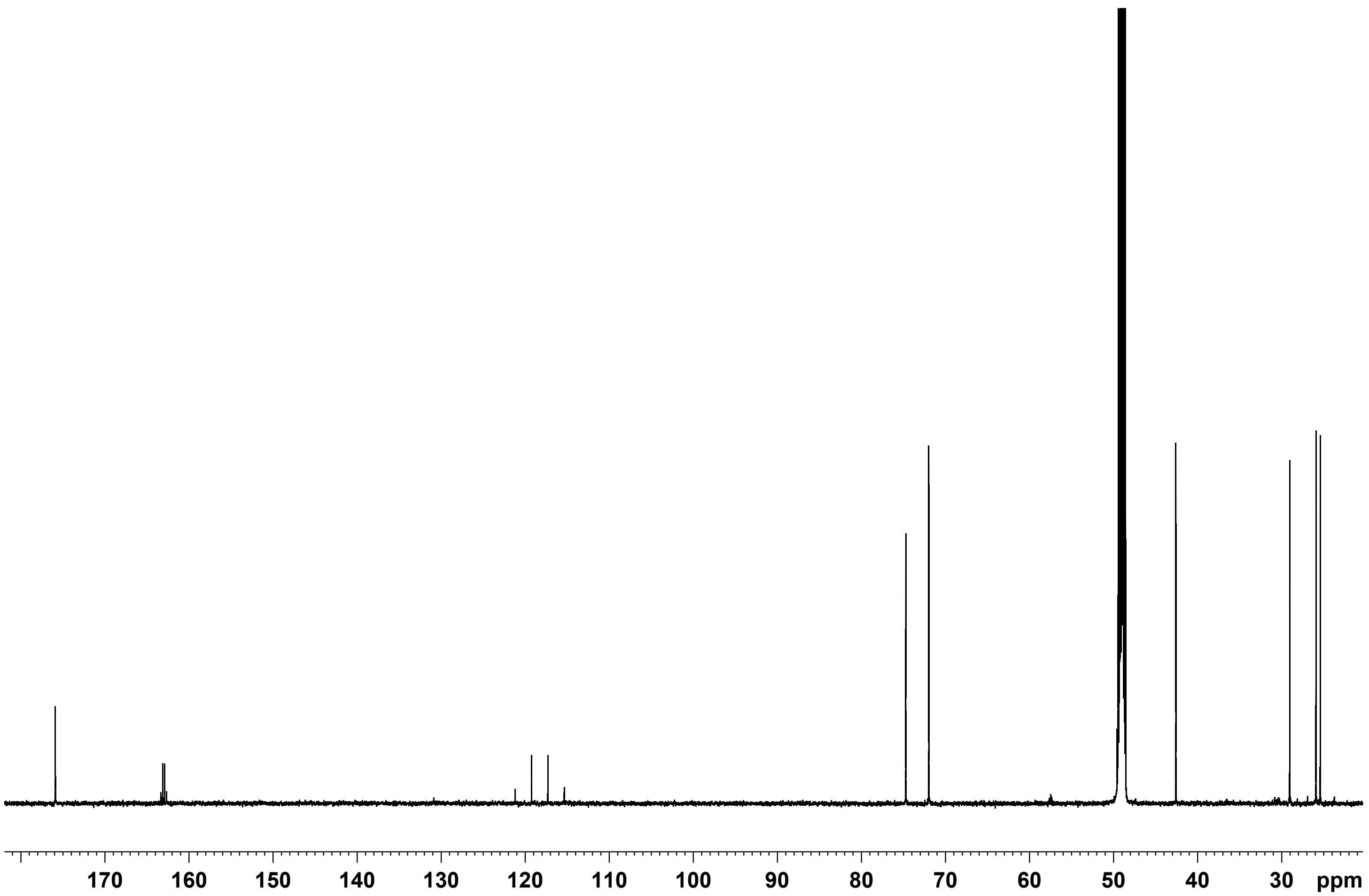

Supplementary Figure 25 | ^{13}C NMR spectrum of *syn*-gonydiol (*syn*-**5**; CD_3OD).

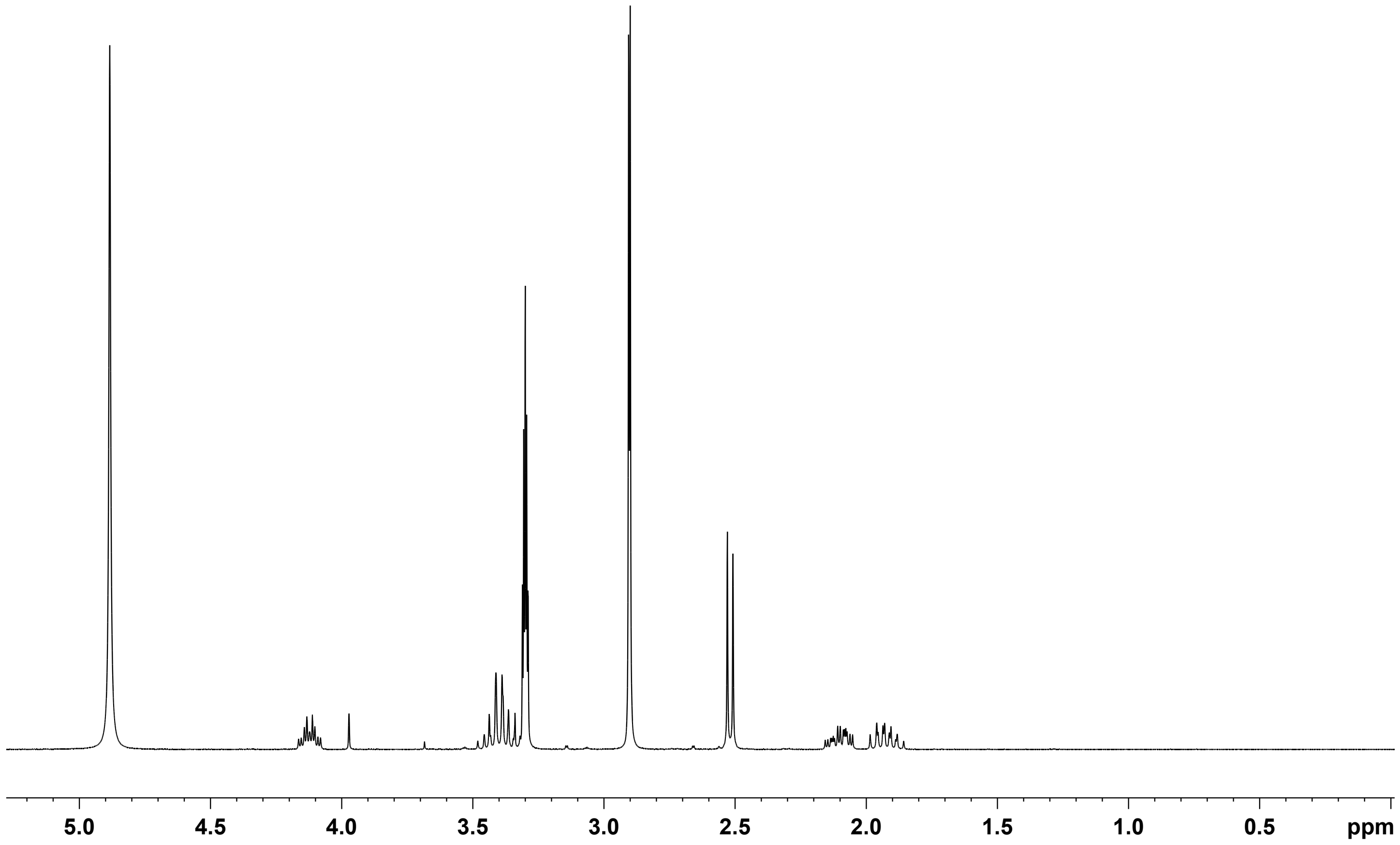

Supplementary Figure 26 | ^{1}H NMR spectrum of *R*-gonyol (*R*-**4**; CD_3OD).

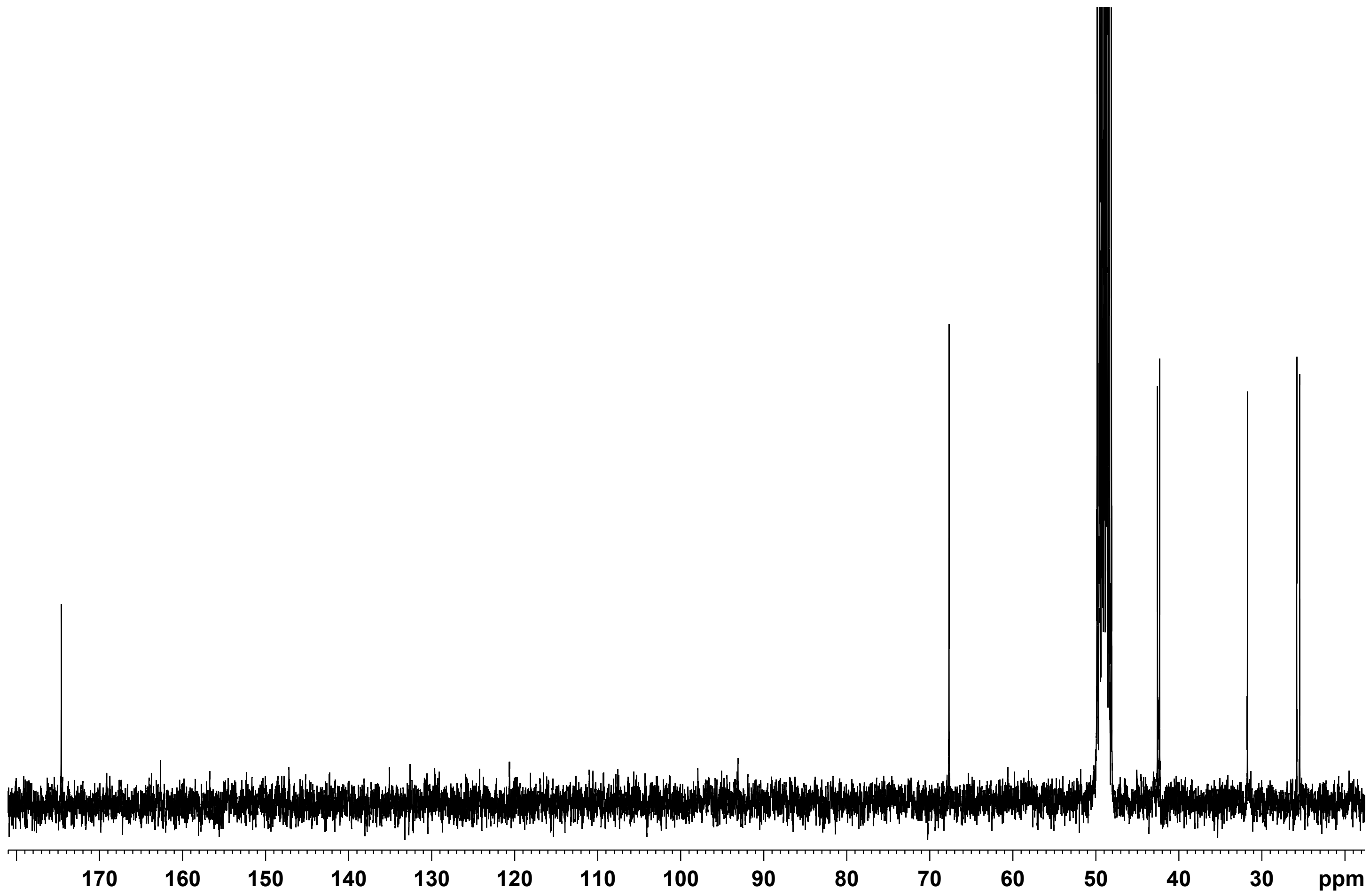

Supplementary Figure 27 | ^{13}C NMR spectrum of *R*-gonyol (*R*-**4**; CD_3OD).

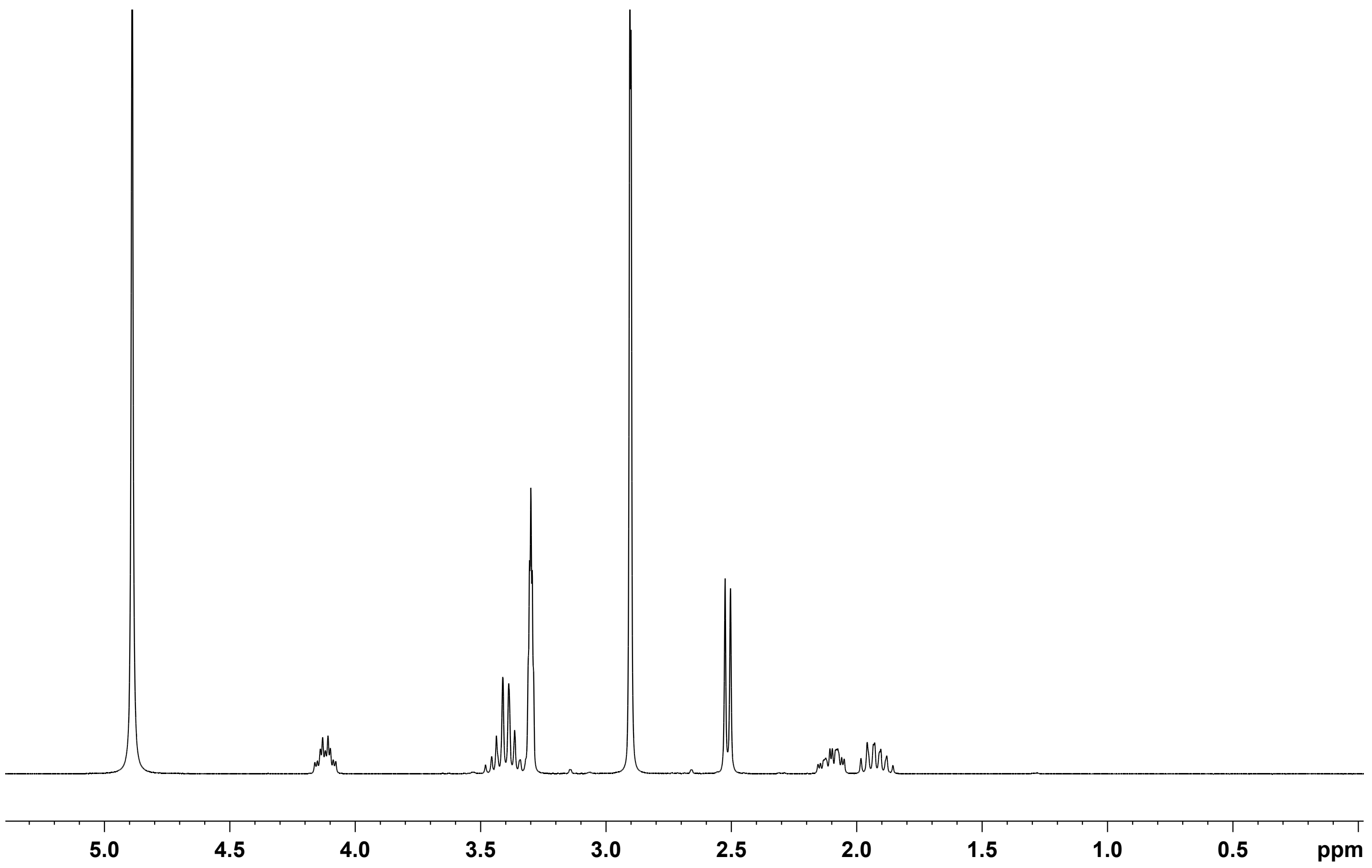

Supplementary Figure 28 | ^{1}H NMR spectrum of *S*-gonyol (*S*-**4**; CD_3OD).

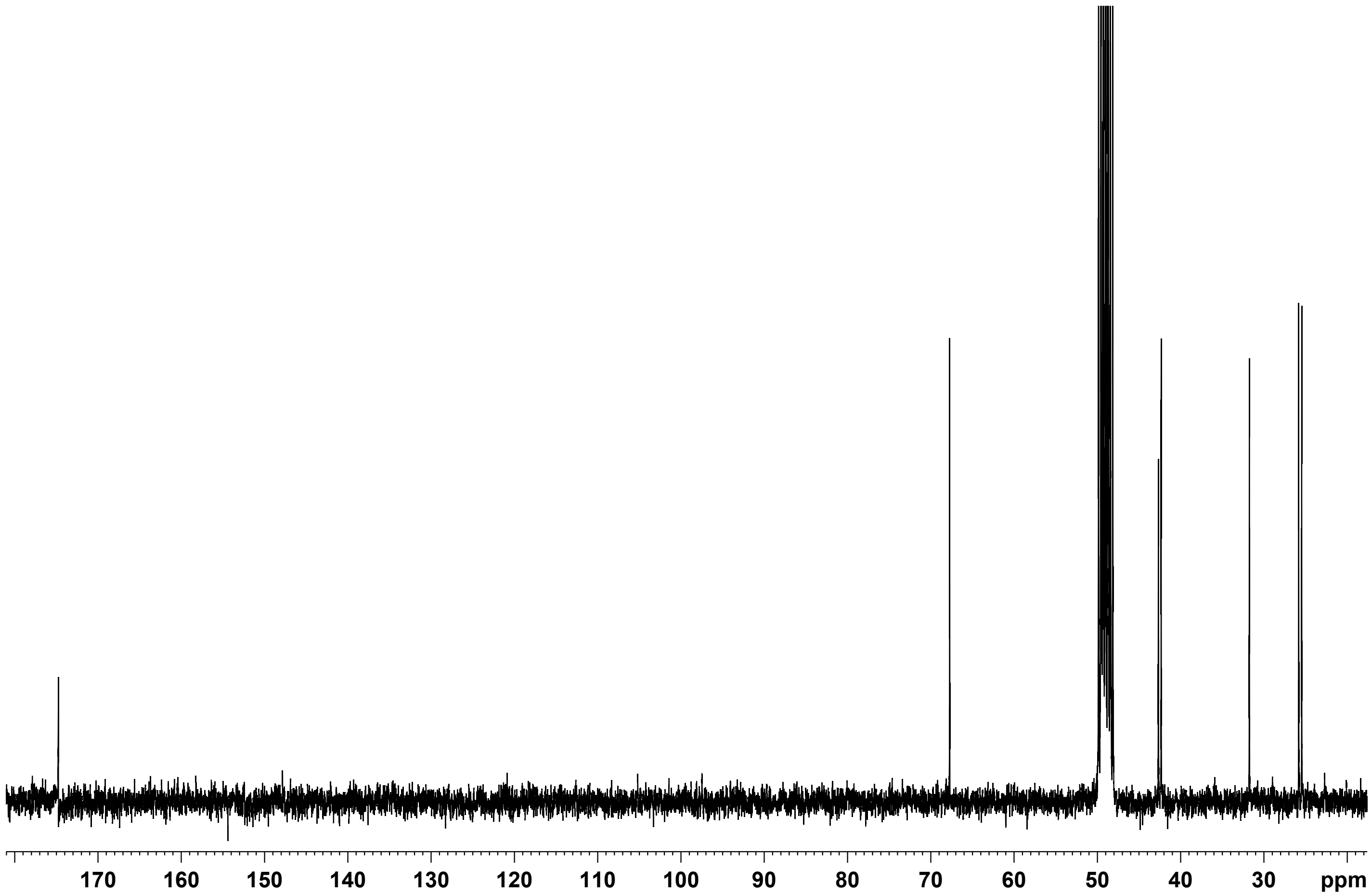

Supplementary Figure 29 | ^{13}C NMR spectrum of *S*-gonyol (*S*-**4**; CD_3OD).

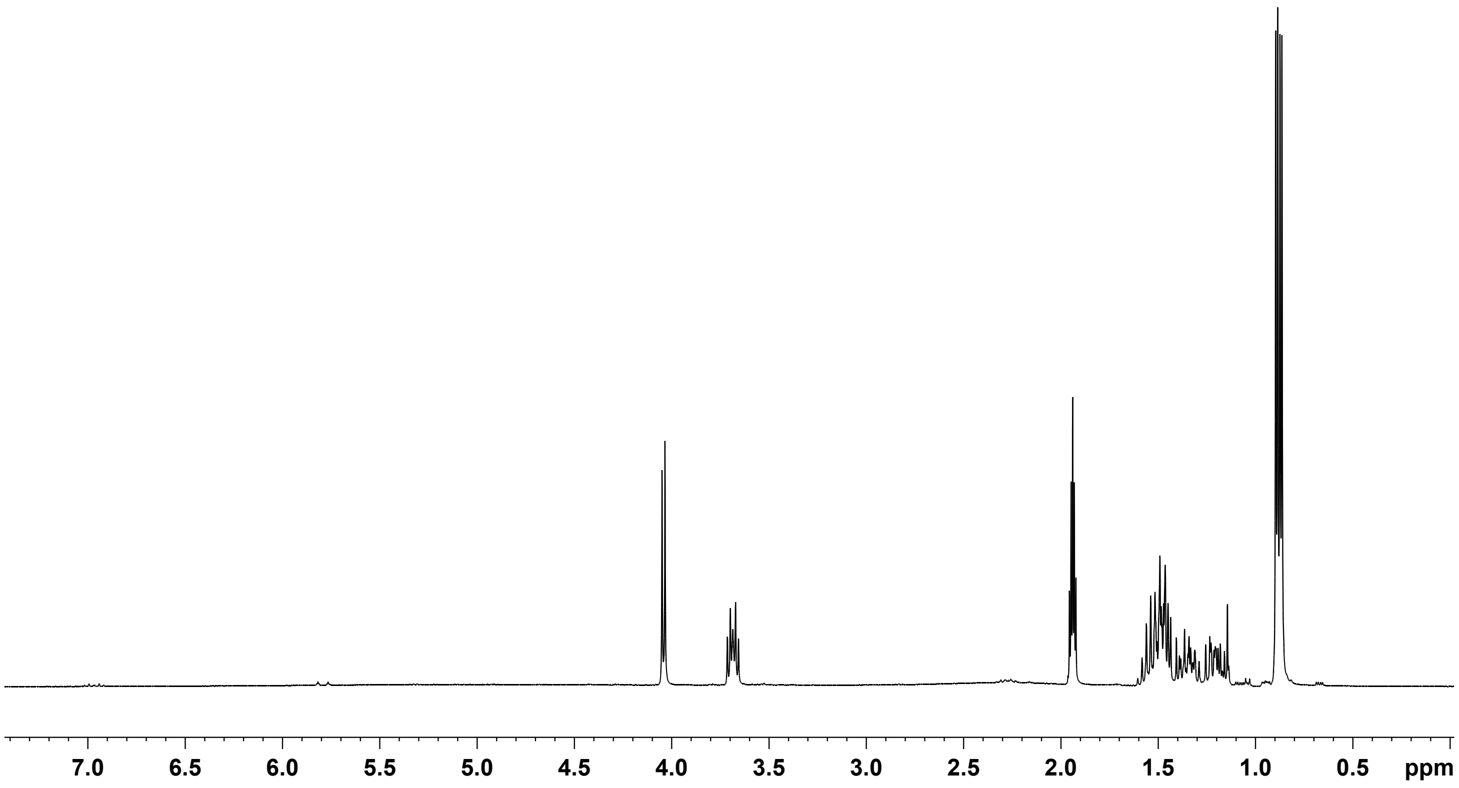

Supplementary Figure 30 | ^{1}H NMR spectrum of carba-gonydiol (**12**; CD_3CN).

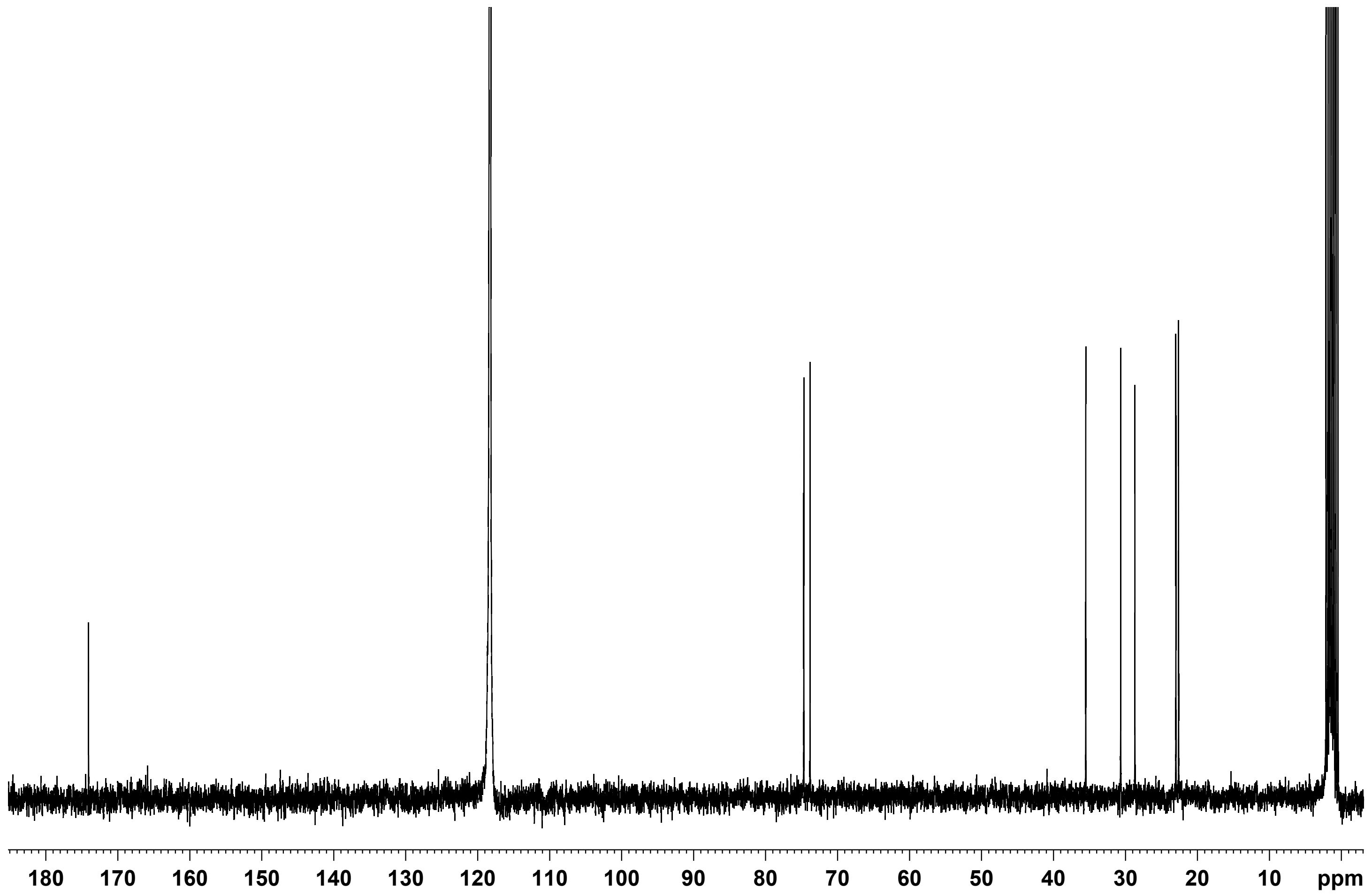

Supplementary Figure 31 | ^{13}C NMR spectrum of carba-gonydiol (**12**; CD_3CN).

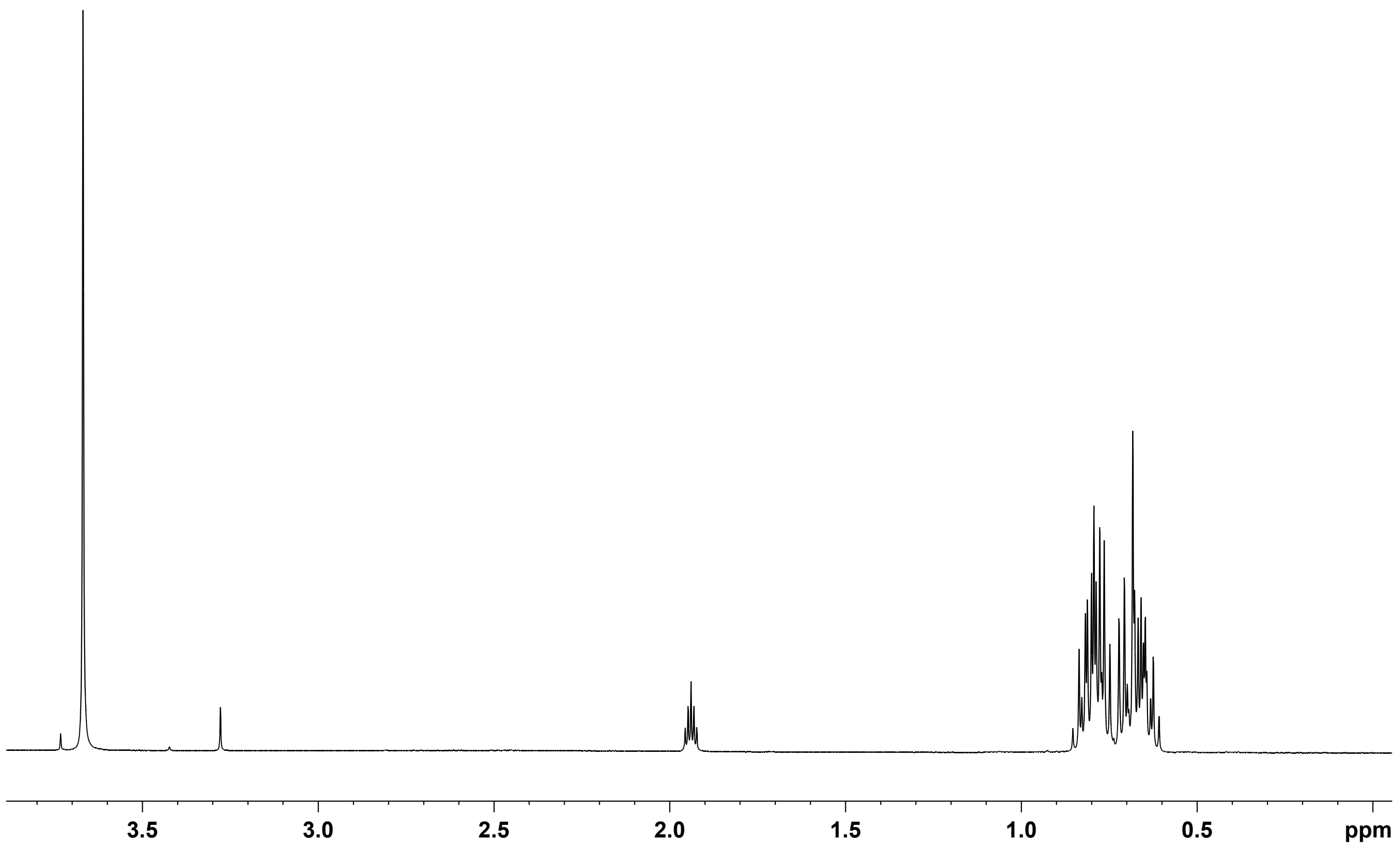

Supplementary Figure 32 | ^{1}H NMR spectrum of trigonic acid (**6**; CD_3CN).

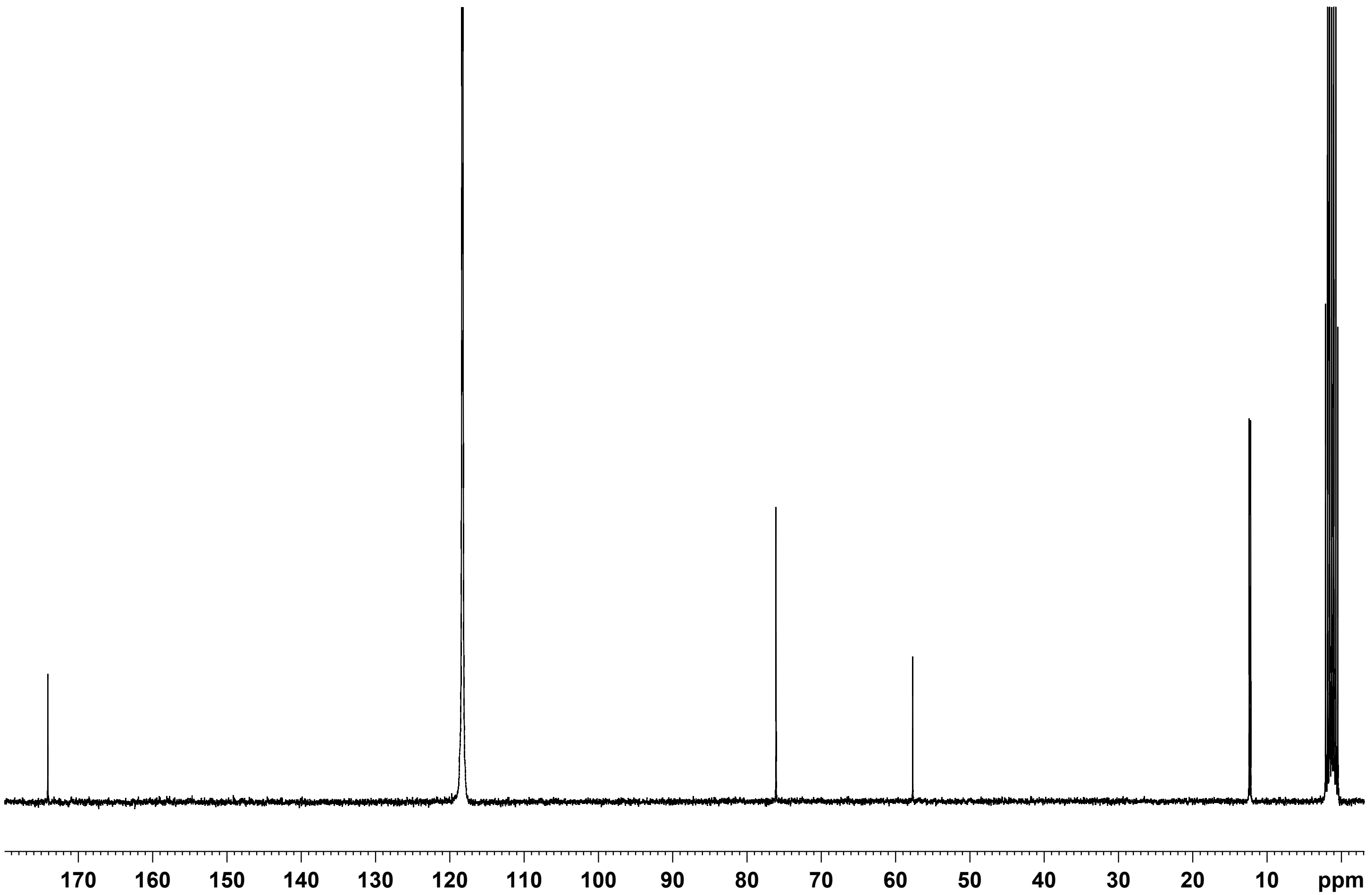

Supplementary Figure 33 | ^{13}C NMR spectrum of trigonic acid (**6**; CD_3CN).

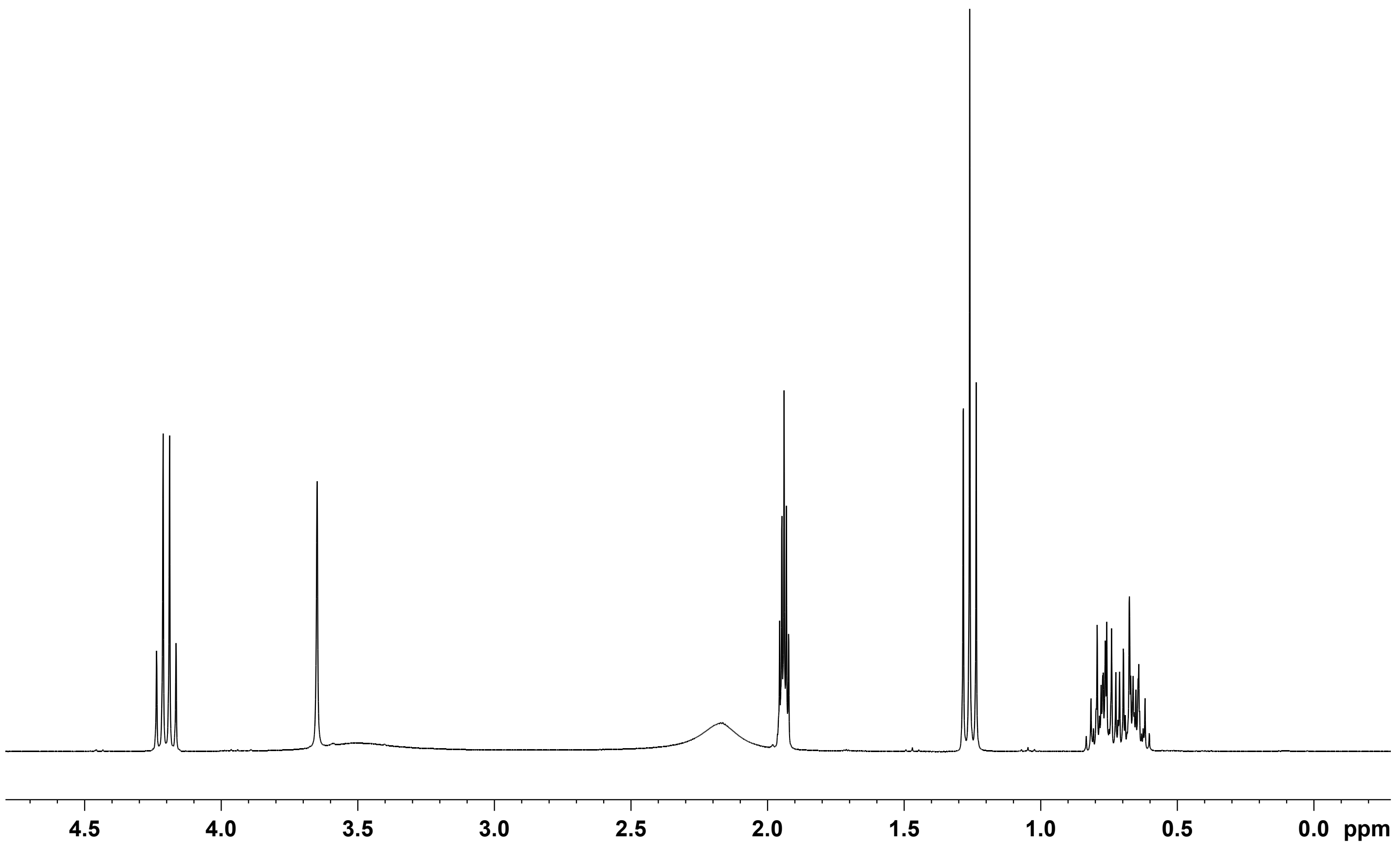

Supplementary Figure 34 | ^{1}H NMR spectrum of ethyl *R*-2-hydroxy-2-(1-hydroxycyclopropyl)acetate (CD_3CN).

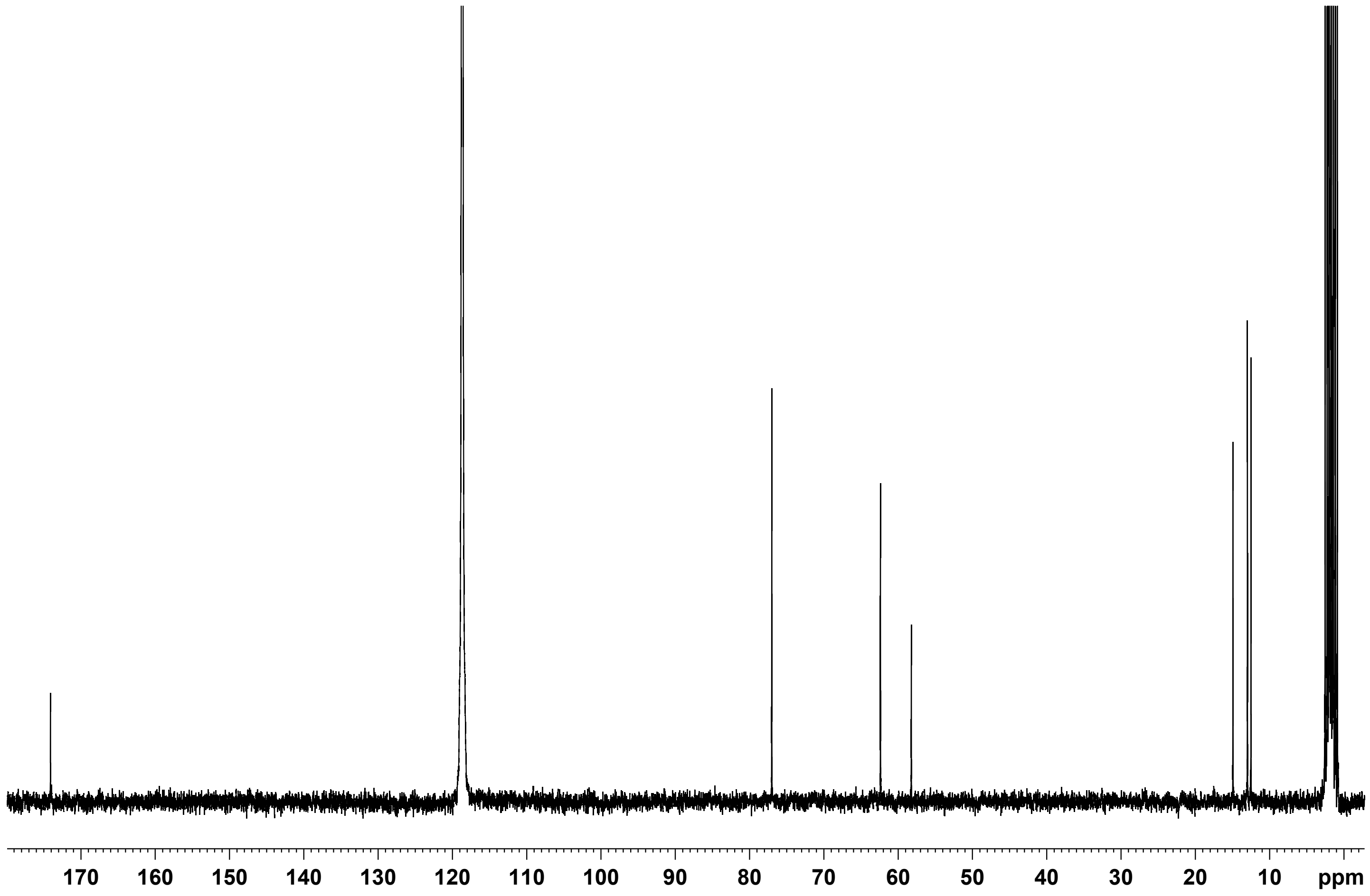

Supplementary Figure 35 | ^{13}C NMR spectrum of ethyl *R*-2-hydroxy-2-(1-hydroxycyclopropyl)acetate (CD_3CN).

Supplementary Tables

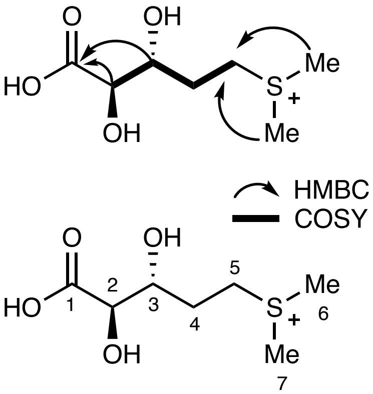

Supplementary Table 1. NMR shifts of gonydiol.

Gonydiol (**5**)		
CD$_3$OD (151/600 MHz, 298 K)		
**Position**	**δ_C**	**δ_H (m, *J* [Hz])**
1	175.4	-
2	75.3	4.16 (d, 4.4)
3	72.4	3.98 (m)
4	27.5	2.13–2.0 (m)
5	42.6	3.48–3.37 (m)
6/7	26.0/25.4	2.91 (s)/2.89 (s)

Supplementary Table 2. X-ray data collection and refinement statistics.

	BurG (holo) NAD⁺, Mg²⁺	*BurG (apo)*	*BurG:7 NAD⁺, Mg²⁺*	*BurG:8 NAD⁺, Mg²⁺*	*BurG:11 NAD⁺, Mg²⁺*
**Crystal parameters**					
Space group	$P2_12_12_1$	$P3_121$	$P2_12_12_1$	$P2_12_12_1$	$P2_12_12_1$
Cell constants	a = 75.5 Å<br>b = 82.7 Å<br>c = 100.9 Å	a = b = 61.3 Å<br>c = 190.0 Å	a = 75.6 Å<br>b = 83.3 Å<br>c = 101.2 Å	a = 75.7 Å<br>b = 84.5 Å<br>c = 46.2 Å	a = 75.7 Å<br>b = 84.0 Å<br>c = 101.8 Å
CPs / AU[a]	2	2	2	1	2
**Data collection**					
Beam line	X06SA, SLS	X06SA, SLS	X06SA, SLS	X06SA, SLS	X06SA, SLS
Wavelength (Å)	1.0	1.0	1.0	1.0	1.0
Resolution range (Å)[b]	301.85 (1.95–1.85)	30–2.9 (3.0–2.9)	30–1.9 (2.0–1.9)	30–1.9 (2.0–1.9)	30–2.05 (2.15–2.05)
No. observations	239383	46397	209399	105003	153538
No. unique reflections[c]	54278	9678	49898	23699	40538
Completeness (%)[b]	99.4 (99.7)	99.0 (100)	97.7 (99.5)	98.4 (99.1)	97.8 (98.6)
R_{merge} (%)[b, d]	9.6 (57.0)	6.5 (58.8)	8.5 (57.1)	7.4 (56.9)	9.7 (55.8)
I/σ (I)[b]	8.8 (2.5)	14.2 (2.0)	10.2 (2.1)	11.9 (2.7)	8.5 (2.9)
**Refinement (REFMAC5)**					
Resolution range (Å)	30–1.85	30–2.9	30–1.9	30–1.9	30–2.05
No. refl. working set	51548	9184	47391	22473	38498
No. refl. test set	2713	483	2494	1181	2026
No. non hydrogen	5574	2527	5545	2756	5541
No. of cofactors	2	-	2	1	2
No. of Mg^{2+} atoms	4	-	4	2	4
No. of ligands	-	-	2	1	2
Solvent	261	10	214	70	220
R_{work}/R_{free} (%)[e]	18.5/21.6	21.9/24.7	19.5/22.8	19.8/22.5	18.8 /21.8
r.m.s.d. bond (Å) / (angle)[f]	0.003/1.2	0.002/1.2	0.003/1.2	0.002/1.2	0.002/1.3
Average B-factor ($Å^2$)					
Protein	24.5	86.5	29.6	30.3	25.4
Ligand	21.9	-	28.9	28.8	22.3
Solvent	29.8	-	32.1	35.6	29.9
Ramachandran Plot (%)[g]	97.4/2.4/0.2	96.8/3.0/0.2	98.5/1.3/0.2	97.8/2.0/0.2	98.2/1.6/0.2
Clashscore, all atoms[h]	2.7	2.4	2.5	1.9	2.5
PDB accession code	7PCC	7PCE	7PCG	7PCI	7PCL

[a] Asymmetric unit

[b] The values in parentheses for resolution range, completeness, R_{merge} and I/σ (I) correspond to the highest resolution shell

[c] Data reduction was carried out with XDS and from a single crystal. Friedel pairs were treated as identical reflections

[d] $R_{merge}(I) = \Sigma_{hkl}\Sigma_j \mid I(hkl)_j - \langle I(hkl)\rangle \mid / \Sigma_{hkl} \Sigma_j I(hkl)_j$, where $I(hkl)_j$ is the jth measurement of the intensity of reflection hkl and <I(hkl)> is the average intensity

[e] $R = \Sigma_{hkl} \mid |F_{obs}| - |F_{calc}| \mid / \Sigma_{hkl} |Fobs|$, where R_{free} is calculated without a sigma cut off for a randomly chosen 5% of reflections, which were not used for structure refinement, and R_{work} is calculated for the remaining reflections

[f] Deviations from ideal bond lengths/angles

[g] Percentage of residues in favoured region / allowed region / outlier region

[h] Number of steric overlaps (> 0.4 Å) per 1000 atoms[52]

Supplementary Table 2. X-ray data collection and refinement statistics (continued).

	BurG:13	*BurG:14 (NADH, Mg^{2+})/*	*BurG-E232Q:12*	*BurG-E232Q:15*
	NADH, Mg^{2+}	*BurG:6 (NAD^+, Mg^{2+})*	*NAD^+, Mg^{2+}*	*NAD^+, Mg^{2+}*
**Crystal parameters**				
Space group	$P2_12_12_1$	$P2_1$	$P2_12_12_1$	$P2_1$
Cell constants	a = 75.8 Å<br>b = 83.6 Å<br>c = 100.7 Å	a = 55.9 Å<br>b = 76.1 Å<br>c = 79.1 Å<br>β = 105.1°	a = 75.7 Å<br>b = 83.4 Å<br>c = 104.8 Å	a = 55.3 Å<br>b = 76.0 Å<br>c = 78.2 Å<br>β = 106.1°
CPs / AU[a]	2	2	2	2
**Data collection**				
Beam line	X06SA, SLS	X06SA, SLS	X06SA, SLS	X06SA, SLS
Wavelength (Å)	1.0	1.0	1.0	1.0
Resolution range (Å)[b]	30–2.05<br>(2.15–2.05)	30–1.6<br>(1.7–1.6)	30–1.55<br>(1.65–1.55)	30–1.35<br>(1.45–1.35)
No. observations	177924	257794	321312	420339
No. unique reflections[c]	40243	82909	93303	139870
Completeness (%)[b]	98.5 (99.3)	98.2 (99.2)	96.4 (98.1)	97.3 (96.4)
R_{merge} (%)[b, d]	11.2 (59.6)	9.1 (57.3)	8.2 (54.9)	6.8 (53.6)
I/σ (I)[b]	8.2 (2.4)	8.1 (2.0)	8.2 (2.1)	8.3 (2.0)
**Refinement (REFMAC5)**				
Resolution range (Å)	30–2.05	30–1.6	30–1.55	30–1.35
No. refl. working set	38214	78752	88624	132865
No. refl. test set	2012	4145	4665	6993
No. non hydrogen	5437	5932	5765	6080
No. of cofactors	2	2/2[i]	2	2
No. of Mg^{2+} atoms	4	4	4	4
No. of ligands	2	2/4[i]	2	2[j]
Solvent	112	473	430	761
R_{work}/R_{free} (%)[e]	20.3/23.9	13.5/15.9	16.9/19.7	15.6/16.9
r.m.s.d. bond (Å) / (angle)[f]	0.003/1.3	0.006/1.4	0.007/1.5	0.006/1.4
Average B-factor ($Å^2$)				
Protein	30.9	18.9	18.7	14.0
Ligand	29.1	15.5	17.4	12.9
Solvent	27.8	28.6	26.7	25.6
Ramachandran Plot (%)[g]	96.8/3.0/0.2	97.7/2.1/0.2	97.7/2.1/0.2	98.3/1.5/0.2
Clashscore, all atoms[h]	2.4	2.3	2.4	1.4
PDB accession code	7PCM	7PCN	7PCO	7PCT

[a] Asymmetric unit

[b] The values in parentheses for resolution range, completeness, R_{merge} and I/σ (I) correspond to the highest resolution shell

[c] Data reduction was carried out with XDS and from a single crystal. Friedel pairs were treated as identical reflections

[d] $R_{merge}(I) = \Sigma_{hkl}\Sigma_j \mid I(hkl)_j - \langle I(hkl)\rangle \mid / \Sigma_{hkl} \Sigma_j I(hkl)_j$, where $I(hkl)_j$ is the jth measurement of the intensity of reflection hkl and $\langle I(hkl)\rangle$ is the average intensity

[e] $R = \Sigma_{hkl} \mid |F_{obs}| - |F_{calc}| \mid / \Sigma_{hkl} |Fobs|$, where R_{free} is calculated without a sigma cut off for a randomly chosen 5% of reflections, which were not used for structure refinement, and R_{work} is calculated for the remaining reflections

[f] Deviations from ideal bond lengths/angles

[g] Percentage of residues in favoured region / allowed region / outlier region

[h] Number of steric overlaps (> 0.4 Å) per 1000 atoms[52]

[i] The active site contains **14** and NADH as well as **6**, DMS and NAD^+ with occupancies of 0.5, respectively

[j] **15**: The metabolite is enol-oxaloacetate and was co-purified from the *E. coli* lysate (Supplementary Fig. 8).

Supplementary Table 3. Bacterial strains used in this study.

Strain	Relevant characteristics	Source or reference
E. coli		
TOP10	General cloning host strain	Invitrogen
XL1-Blue	General cloning host strain; Tet^R	Stratagene
Rosetta2 (DE3)	Protein production host strain carrying rare codon; Cm^R	Novagen
BL21 (DE3)	Protein production host strain	NEB
pET28a-*burC*	Overexpressing *burC*	This study
pET28a-*burG*	Overexpressing *burG*	This study
pET28a-*burG*_E232Q	Overexpression *burG*_E232Q	This study
B. thailandensis		
E264	Prototroph; environmental isolate	DSMZ
E264 *Pbur*	Promotor of *burA* was exchanged with Δ*PthaA*;Tet^R	[9]
E264 *Pbur, burG*::*Kan*	Kan cassette inserted into *burG*; Tet^R Kan^R	This study

Supplementary Table 4. Primers used in this study.

Primer	Nucleotide sequence (5' to 3')	Source or reference
Isomerase fw	TCG ACG ATG AAC GGC ACC CG	This study
Isomerase rv	AAG CGC TCG GCG AGC CAT CG	This study
Isomerase fw2	CGT GAT CGG CTA CGG CAT CC	This study
Isomerase rv2	GCG TAG TCG TTC GAT GCC TG	This study
burC4-fw-NdeI	GGT CAT ATG GAT AGC TTC ATC GAA CTG CAA	This study
burC-rv-EcoRI	GGT GAA TTC TCA GTC GGT CAG GCT GAC GAG	This study
burG-fw-NheI	GGT GCT AGC AAC GAT CTC ATC TAT CAG GAC GAA CAC	This study
burG-rv-HindIII	GGT AAG CTT TCA TCG CGC GGC CTC AGG TTC G	This study

Supplementary Table 5. Vectors and plasmids used in this study.

Plasmid	Relevant characteristics	Source or reference
**Vectors**		
pGEM® T-easy	TA cloning vector; Amp^R	Promega
pCR™ 2.1	TA cloning vector; Kan^R	Invitrogen
pJET 1.2	Blunt end cloning vector; Amp^R	ThermoScientific
pET28a(+)	His6-tagged protein overexpression vector using the T7 bacteriophage promotor; Kan^R	Novagen
**Plasmids**		
pGEM-Isomerase	pGEM T-easy containing *burG* from *B. thailandensis*; Amp^R	This study
pGEM-burG::Kan	pGEM-Isomerase inserting Kan^R in *burG*; Amp^R Kan^R	This study
pCR-burC	pCR 2.1 containing *burC* from *B. thailandensis*; Kan^R	This study
pET28a-burC	pET28a encoding *burC*; Kan^R	This study
pJET-burG	pJET 1.2 containing *burG*; Amp^R	This study
pET28a-burG	pET28a encoding *burG*; Kan^R	This study
pJET-burG_E232Q	pJET 1.2 containing synthetic *burG*_E232Q; Amp^R	This study
pET28a-burG_E232Q	pET28a encoding *burG*_E232Q; Kan^R	This study

Supplementary Table 6. KARI sequences used for phylogenetic analysis.

Protein	Source	Accession No.
**Dimeric bacterial KARI**		
IlvC_Avit	*Agrobacterium vitis*	WP_070165060.1
ILVC_AZOVD	*Azotobacter vinelandii* DJ	C1DFH7.1
IlvC_Bcon	*Burkholderia contaminans* (multi species)	WP_098551046.1
IlvC_Bokl	*Burkholderia oklahomensis* (multi species)	WP_010102500.1
IlvC_Bubo	*Burkholderia ubonensis* (multi species)	WP_042585926.1
ILVC_BURP6	*Burkholderia pseudomallei* 668	A3N7K4.1
ILVC_BURTA	*Burkholderia thailandensis* E264	Q2SZP8
ILVC_CHRVO	*Chromobacterium violaceum* ATCC 12472	Q7P0H9.1
ILVC_CORGL	*Corynebacterium glutamicum* ATCC 13032	Q57179.1
ILVC_CORU7	*Corynebacterium urealyticum* DSM 7109	B1VG26.1
ILVC_CROS5	*Crocosphaera subtropica* ATCC 51142	B1WNP1.1
ILVC_GLOC7	*Gloeothece citriformis* PCC 7424	B7KF23.1
ILVC_GLOVI	*Gloeobacter violaceus* PCC 7421	Q7NH80.1
IlvC_Mnig	*Micromonospora nigra*	WP_091084837.1
ILVC_MYCTU	*Mycobacterium tuberculosis* H37Rv	P9WKJ7.2
ILVC_MYCUA	*Mycobacterium ulcerans* Agy99	A0PPY3
IlvC_Niri	*Nitrospirillum iridis*	WP_184805695.1
ILVC_PROM0	*Prochlorococcus marinus* str. MIT 9301	A3PEE9.1
ILVC_PROM3	*Prochlorococcus marinus* str. MIT 9303	A2CB87.1
IlvC_PIrc	*Pseudomonas* sp. Irchel s3b6	WP_003228216.1
IlvC_Ssyr	*Saccharothrix syringae*	WP_033431529.1
ILVC_STAA	*Staphylococcus aureus*	6VO2
ILVC_STAA8	*Staphylococcus aureus* subsp. *aureus* NCTC 8325	Q2FWK4.1
LVC_STRAW	*Streptomyces avermitilis* MA-4680	Q59818.2
ILVC1_STRCO	*Streptomyces coelicolor* A3(2)	Q9Z565.1
IlvC_Sgle	*Streptomyces glebosus* (multi species)	WP_085925153.1
ILVC_STRGG	*Streptomyces griseus* subsp. *griseus* NBRC 13350	B1VZ72.1
ILVC_Smob	*Streptomyces mobaraensis* DSM 40847	EME98748.1
ILVC_SYNE7	*Synechococcus elongatus* PCC 7942	Q31MY7.2
ILVC_SYNJA	*Synechococcus* sp. JA-3-3Ab	Q2JXL2.1
ILVC_XANOP	*Xanthomonas oryzae* pv. *oryzae* PXO99A	B2SNG5
ILVC_XANCP	*Xanthomonas campestris* pv. *campestris* ATCC 33913	Q8P5L5
**Pseudodimeric bacterial KARI**		
ILVC_BUCAI	*Buchnera aphidicola* subsp. *Acyrthosiphon pisum* APS	P57655
ILVC_BUCAP	*Buchnera aphidicola* subsp. *Schizaphis graminum* Sg	O51888
ILVC_ECOLI	*E. coli*	P05793.4
ILVC_ECO5E	*E. coli*	B5YY23.1
ILVC_HAEIN	*Haemophilus influenzae* ATCC 51907	P44822
ILVC_HAEIG	*Haemophilus influenzae* PittEE	A5UHH1.1
ILVC_SALAR	*Salmonella arizonae* ATCC BAA-731	A9MJN4
ILVC_SALCH	*Salmonella choleraesuis* SC-B67	Q57HU2
ILVC_VIBCH	*Vibrio cholerae* ATCC 39315	Q9KVI4
ILVC_VIBPA	*Vibrio parahaemolyticus* RIMD 2210633	Q87TN4
ILVC_YERE8	*Yersinia enterocolitica* NCTC 13174	A1JI57
ILVC_YERPY	*Yersinia pseudotuberculosis* strain YPIII	B1JQ26

**Eucaryotic KARI**		
ILV5_ARATH	chloroplastic *Arabidopsis thaliana*	Q05758
ILV5_NEUCR	mitochondrial *Neurospora crassa* ATCC 24698	P38674
ILV5_ORYSJ	chloroplastic *Oryza sativa* subsp. *japonica*	Q65XK0
ILV5_SCHPO	mitochondrial *Schizosaccharomyces pombe* ATCC 24843	P78827
ILV5_SPIOL	chloroplastic *Spinacia oleracea*	Q01292
ILV5_YEAST	mitochondrial *Saccharomyces cerevisiae* ATCC 204508	P06168
**BurG in *Burkholderia pseudomallei* complex bacteria**		
BurG_Bcon	*Burkholderia contaminans*	WP_047853400.1
BurG_Bokl	*Burkholderia oklahomensis*	WP_010107715.1
BurG_Bpse	*Burkholderia pseudomallei*	WP_009975226.1
BurG_Btha	*Burkholderia thailandensis* E264	ABC34926.1
BurG_Bubo	*Burkholderia ubonensis*	WP_059480468.1
**BurG orthologues in other bacteria**		
BurG_Avit	*Agrobacterium vitis*	WP_071204337.1
BurG_Mnig	*Micromonospora nigra*	WP_091082404.1
BurG_Msp	*Micromonospora* sp. MMS20-R2-29	MBM7086350.1
BurG_Niri	*Nitrospirillum iridis*	WP_184807076.1
BurG_Nchr	*Nocardiopsis chromatogenes*	WP_017624912.1
BurG_PIrc	*Pseudomonas* sp. Irchel s3b6	WP_095144278.1
BurG_Sbry	*Streptomyces bryophytorum*	MBM9435269.1
BurG_Sgle	*Streptomyces glebosus*	GFE12740.1
BurG_Smob	*Streptomyces mobaraensis* DSM 40847	EME99410.1
BurG_Ssyr	*Saccharothrix syringae*	WP_033429234.1

References

1 Galyov, E. E., Brett, P. J. & DeShazer, D. Molecular insights into *Burkholderia pseudomallei* and *Burkholderia mallei* pathogenesis. *Annu. Rev. Microbiol.* **64**, 495-517, (2010).

2 Cruz-Migoni, A. *et al.* A *Burkholderia pseudomallei* toxin inhibits helicase activity of translation factor eIF4A. *Science* **334**, 821-824, (2011).

3 Franke, J., Ishida, K. & Hertweck, C. Evolution of siderophore pathways in human pathogenic bacteria. *J. Am. Chem. Soc.* **136**, 5599-5602, (2014).

4 Franke, J., Ishida, K. & Hertweck, C. Plasticity of the malleobactin pathway and its impact on siderophore action in human pathogenic bacteria. *Chem. Eur. J.* **21**, 8010-8014, (2015).

5 Franke, J., Ishida, K., Ishida-Ito, M. & Hertweck, C. Nitro versus hydroxamate in siderophores of pathogenic bacteria: effect of missing hydroxylamine protection in malleobactin biosynthesis. *Angew. Chem. Int. Ed.* **52**, 8271-8275, (2013).

6 Biggins, J. B., Kang, H. S., Ternei, M. A., DeShazer, D. & Brady, S. F. The chemical arsenal of *Burkholderia pseudomallei* is essential for pathogenicity. *J. Am. Chem. Soc.* **136**, 9484-9490, (2014).

7 Klaus, J. R. *et al.* Secondary metabolites from the *Burkholderia pseudomallei* complex: structure, ecology, and evolution. *J. Ind. Microbiol. Biotechnol.* **47**, 877-887, (2020).

8 Biggins, J. B., Ternei, M. A. & Brady, S. F. Malleilactone, a polyketide synthase-derived virulence factor encoded by the cryptic secondary metabolome of *Burkholderia pseudomallei* group pathogens. *J. Am. Chem. Soc.* **134**, 13192-13195, (2012).

9 Franke, J., Ishida, K. & Hertweck, C. Genomics-driven discovery of burkholderic acid, a noncanonical, cryptic polyketide from human pathogenic *Burkholderia* species. *Angew. Chem. Int. Ed.* **51**, 11611-11615, (2012).

10 Gutierrez, M. G., Yoder-Himes, D. R. & Warawa, J. M. Comprehensive identification of virulence factors required for respiratory melioidosis using Tn-seq mutagenesis. *Front. Cell. Infect. Microbiol.* **5**, 78, (2015).

11 Moule, M. G. *et al.* Characterization of New Virulence Factors Involved in the Intracellular Growth and Survival of *Burkholderia pseudomallei*. *Infect. Immun.* **84**, 701-710, (2016).

12 Klaus, J. R. *et al.* Malleilactone Is a Burkholderia pseudomallei Virulence Factor Regulated by Antibiotics and Quorum Sensing. *J. Bacteriol.* **200**, e00008-00018, (2018).

13 Trottmann, F. *et al.* Cyclopropanol Warhead in Malleicyprol Confers Virulence of Human- and Animal-Pathogenic *Burkholderia* Species. *Angew. Chem. Int. Ed.* **58**, 14129-14133, (2019).

14 Nicolaev, A. & Orellana, A. Transition-Metal-Catalyzed C–C and C–X Bond-Forming Reactions Using Cyclopropanols. *Synthesis* **48**, 1741-1768, (2016).

15 Rosa, D., Nikolaev, A., Nithiy, N. & Orellana, A. Palladium-Catalyzed Cross-Coupling Reactions of Cyclopropanols. *Synlett* **26**, 441-448, (2015).

16 Shunsuke, C., Zhengyan, C., Atta Atta, E. B. S. & Koichi, N. Generation of β-Keto Radicals from Cyclopropanols Catalyzed by $AgNO_3$. *Chem. Lett.* **35**, 18-19, (2006).

17 Trottmann, F. *et al.* Sulfonium Acids Loaded onto an Unusual Thiotemplate Assembly Line Construct the Cyclopropanol Warhead of a *Burkholderia* Virulence Factor. *Angew. Chem. Int. Ed.* **59**, 13511-13515, (2020).

18 Ueoka, R., Bortfeld-Miller, M., Morinaka, B. I., Vorholt, J. A. & Piel, J. Toblerols: Cyclopropanol-Containing Polyketide Modulators of Antibiosis in Methylobacteria. *Angew. Chem. Int. Ed.* **57**, 977-981, (2018).
19 Patel, K. M. *et al.* Crystal Structures of *Staphylococcus aureus* Ketol-Acid Reductoisomerase in Complex with Two Transition State Analogues that Have Biocidal Activity. *Chem. Eur. J.* **23**, 18289-18295, (2017).
20 Brown, S. P. *et al.* Compounds that inhibit MCL-1 Protein. WO2017/147410A1 (2017).
21 Kolb, H. C., VanNieuwenhze, M. S. & Sharpless, K. B. Catalytic Asymmetric Dihydroxylation. *Chem. Rev.* **94**, 2483-2547, (1994).
22 Gabler, F. *et al.* Protein Sequence Analysis Using the MPI Bioinformatics Toolkit. *Curr. Protoc. Bioinform.* **72**, e108, (2020).
23 Brinkmann-Chen, S. *et al.* General approach to reversing ketol-acid reductoisomerase cofactor dependence from NADPH to NADH. *Proc. Natl. Acad. Sci. U.S.A.* **110**, 10946-10951, (2013).
24 Brinkmann-Chen, S., Cahn, J. K. B. & Arnold, F. H. Uncovering rare NADH-preferring ketol-acid reductoisomerases. *Metab. Eng.* **26**, 17-22, (2014).
25 Stein, A. J. & Geiger, J. H. The crystal structure and mechanism of 1-L-myo-inositol- 1-phosphate synthase. *J. Biol. Chem.* **277**, 9484-9491, (2002).
26 Thoden, J. B., Frey, P. A. & Holden, H. M. Molecular structure of the NADH/UDP-glucose abortive complex of UDP-galactose 4-epimerase from *Escherichia coli*: implications for the catalytic mechanism. *Biochemistry* **35**, 5137-5144, (1996).
27 Yamauchi, N. & Kakinuma, K. Enzymic carbocycle formation in microbial secondary metabolism. The mechanism of the 2-deoxy-scyllo-inosose synthase reaction as a crucial step in the 2-deoxystreptamine biosynthesis in *Streptomyces fradiae*. *J. Org. Chem* **60**, 5614-5619, (2002).
28 Holzer, H. & Holldorf, A. Hydroxypyruvate in *Methods of Enzymatic Analysis* (ed. Bergmeyer, H.-U.) 260-262 (Academic Press, New York and London, 1965).
29 Tadrowski, S. *et al.* Metal Ions Play an Essential Catalytic Role in the Mechanism of Ketol-Acid Reductoisomerase. *Chem. Eur. J.* **22**, 7427-7436, (2016).
30 Gu, L. *et al.* Metamorphic enzyme assembly in polyketide diversification. *Nature* **459**, 731-735, (2009).
31 Jin, W. B., Wu, S., Jian, X. H., Yuan, H. & Tang, G. L. A radical *S*-adenosyl-L-methionine enzyme and a methyltransferase catalyze cyclopropane formation in natural product biosynthesis. *Nat. Commun.* **9**, 2771, (2018).
32 Neumann, C. S. & Walsh, C. T. Biosynthesis of (-)-(1*S*,2*R*)-allocoronamic acyl thioester by an Fe(II)-dependent halogenase and a cyclopropane-forming flavoprotein. *J. Am. Chem. Soc.* **130**, 14022-14023, (2008).
33 Ramalingam, K., Lee, K. M., Woodard, R. W., Bleecker, A. B. & Kende, H. Stereochemical course of the reaction catalyzed by the pyridoxal phosphate-dependent enzyme 1-aminocyclopropane-1-carboxylate synthase. *Proc. Natl. Acad. Sci. U.S.A.* **82**, 7820-7824, (1985).
34 Vaillancourt, F. H., Yeh, E., Vosburg, D. A., O'Connor, S. E. & Walsh, C. T. Cryptic chlorination by a non-haem iron enzyme during cyclopropyl amino acid biosynthesis. *Nature* **436**, 1191-1194, (2005).
35 Zha, L. *et al.* Colibactin assembly line enzymes use *S*-adenosylmethionine to build a cyclopropane ring. *Nat. Chem. Biol.* **13**, 1063-1065, (2017).

36 Vizcaino, M. I. & Crawford, J. M. The colibactin warhead crosslinks DNA. *Nat. Chem.* **7**, 411-417, (2015).
37 Wilson, M. R. *et al.* The human gut bacterial genotoxin colibactin alkylates DNA. *Science* **363**, (2019).
38 Thibodeaux, C. J., Chang, W. C. & Liu, H. W. Enzymatic chemistry of cyclopropane, epoxide, and aziridine biosynthesis. *Chem. Rev.* **112**, 1681-1709, (2012).
39 Coelho, P. S., Brustad, E. M., Kannan, A. & Arnold, F. H. Olefin Cyclopropanation via Carbene Transfer Catalyzed by Engineered Cytochrome P450 Enzymes. *Science* **339**, 307-310, (2013).
40 Katoh, K., Rozewicki, J. & Yamada, K. D. MAFFT online service: multiple sequence alignment, interactive sequence choice and visualization. *Brief. Bioinform.* **20**, 1160-1166, (2017).
41 Nguyen, L.-T., Schmidt, H. A., von Haeseler, A. & Minh, B. Q. IQ-TREE: A Fast and Effective Stochastic Algorithm for Estimating Maximum-Likelihood Phylogenies. *Mol. Biol. Evol* **32**, 268-274, (2014).
42 Kalyaanamoorthy, S., Minh, B. Q., Wong, T. K. F., von Haeseler, A. & Jermiin, L. S. ModelFinder: fast model selection for accurate phylogenetic estimates. *Nat. Methods* **14**, 587-589, (2017).
43 Minh, B. Q., Nguyen, M. A. T. & von Haeseler, A. Ultrafast Approximation for Phylogenetic Bootstrap. *Mol. Biol. Evol.* **30**, 1188-1195, (2013).
44 Kumar, S., Stecher, G. & Tamura, K. MEGA7: Molecular Evolutionary Genetics Analysis Version 7.0 for Bigger Datasets. *Mol. Biol. Evol.* **33**, 1870-1874, (2016).
45 Taber, D. F., DeMatteo, P. W. & Hassan, R. A. Simplified Preparation of Dimethyldioxirane (DMDO). *Org. Synth.* **90**, 350-357, (2013).
46 Esnouf, R. M. An extensively modified version of MolScript that includes greatly enhanced coloring capabilities. *J. Mol. Graph. Model.* **15**, 132-134, (1997).
47 Kabsch, W. XDS. *Acta Crystallogr. D* **66**, 125-132, (2010).
48 McCoy, A. J. *et al.* Phaser crystallographic software. *J. Appl. Crystallogr.* **40**, 658-674, (2007).
49 Emsley, P. & Cowtan, K. Coot: model-building tools for molecular graphics. *Acta Crystallogr. D* **60**, 2126-2132, (2004).
50 Murshudov, G. N. *et al.* REFMAC5 for the refinement of macromolecular crystal structures. *Acta Crystallogr. D* **67**, 355-367, (2011).
51 Langer, G., Cohen, S. X., Lamzin, V. S. & Perrakis, A. Automated macromolecular model building for X-ray crystallography using ARP/wARP version 7. *Nat. Protoc.* **3**, 1171-1179, (2008).
52 Chen, V. B. *et al.* MolProbity: all-atom structure validation for macromolecular crystallography. *Acta Crystallogr. D* **66**, 12-21, (2010).
