# Supplementary material for: Pathogenic bacteria remodel central metabolic enzyme to build a cyclopropanol warhead
Source: Nat Chem. 2022 Jul 29;14(8):884–90. doi: 10.1038/s41557-022-01005-z (PMC9359912; doi:10.1038/s41557-022-01005-z)
Supplement: Supplementary file 2 — Reporting Summary [file 41557_2022_1005_MOESM2_ESM.pdf]

nature portfolio

Corresponding author(s): Michael Groll, Christian Hertweck

Last updated by author(s): May 18, 2022

Reporting Summary

Nature Portfolio wishes to improve the reproducibility of the work that we publish. This form provides structure for consistency and transparency in reporting. For further information on Nature Portfolio policies, see our Editorial Policies and the Editorial Policy Checklist.

Statistics

For all statistical analyses, confirm that the following items are present in the figure legend, table legend, main text, or Methods section.

n/a	Confirmed	
☐	☒	The exact sample size (*n*) for each experimental group/condition, given as a discrete number and unit of measurement
☐	☒	A statement on whether measurements were taken from distinct samples or whether the same sample was measured repeatedly
☒	☐	The statistical test(s) used AND whether they are one- or two-sided<br>*Only common tests should be described solely by name; describe more complex techniques in the Methods section.*
☒	☐	A description of all covariates tested
☒	☐	A description of any assumptions or corrections, such as tests of normality and adjustment for multiple comparisons
☐	☒	A full description of the statistical parameters including central tendency (e.g. means) or other basic estimates (e.g. regression coefficient) AND variation (e.g. standard deviation) or associated estimates of uncertainty (e.g. confidence intervals)
☒	☐	For null hypothesis testing, the test statistic (e.g. *F*, *t*, *r*) with confidence intervals, effect sizes, degrees of freedom and *P* value noted<br>*Give P values as exact values whenever suitable.*
☒	☐	For Bayesian analysis, information on the choice of priors and Markov chain Monte Carlo settings
☒	☐	For hierarchical and complex designs, identification of the appropriate level for tests and full reporting of outcomes
☒	☐	Estimates of effect sizes (e.g. Cohen's *d*, Pearson's *r*), indicating how they were calculated

Our web collection on statistics for biologists contains articles on many of the points above.

Software and code

Policy information about availability of computer code

Data collection	BLAST+, Version 2.11.0 was used to identify orthologues of BurG.<br>MAFFT, Version 7 was used to align amino acid sequences for phylogenetic tree construction.<br>IQ-TREE was used for construction of the phylogenetic tree.<br>NMR spectra were recorded using TopSpin, Version 3.2 (Bruker).<br>In silico cloning was performed with Clone Manager, Version 10.<br>Extinction coefficients for the determination of protein concentrations were determined with ProtParam.
Data analysis	HHpred was used for protein sequence analysis of BurG.<br>MEGA7 was used to display the phylogenetic tree.<br>Compound Discoverer, Version 2.1, SP1, Thermo Fisher Scientific was used for metabolomics analysis.<br>NMR spectra were visualised and analysed using TopSpin Version 3.2.<br>Mass spectrometry data was analysed using Xcalibur version 4.3.73.11 (LCMS) and Chromeleon Version 7.3 (GCMS); both from Thermo Fisher Scientific.<br>Crystallographic characterisation of BurG: XDS, CCP4, Coot, Pymol, CHARMM, TURBOMOLE, VMD, Compass.<br>GraphPad Prism (8.2.1) was used to analyse data obtained from substrate saturation kinetics and to construct graphs from raw data.<br>Adobe Illustrator (25.3.1) was used to construct figures.

For manuscripts utilizing custom algorithms or software that are central to the research but not yet described in published literature, software must be made available to editors and reviewers. We strongly encourage code deposition in a community repository (e.g. GitHub). See the Nature Portfolio guidelines for submitting code & software for further information.

Data

Policy information about availability of data

All manuscripts must include a data availability statement. This statement should provide the following information, where applicable:

- Accession codes, unique identifiers, or web links for publicly available datasets
- A description of any restrictions on data availability
- For clinical datasets or third party data, please ensure that the statement adheres to our policy

All data are available in the main text, the Supplementary Information, the source data or via the RCSB Protein Data Bank. The corresponding PDB numbers are 7PCC, 7PCE, 7PCG, 7PCI, 7PCL, 7PCM, 7PCN, 7PCO, 7PCT. NMR raw files of natural gonydiol and MS raw files used for comparative metabolomics analysis (Fig. 2a, Supplementary Fig. 1) are deposited on Zenodo (https://doi.org/10.5281/zenodo.6554506) and available without restrictions.

Field-specific reporting

Please select the one below that is the best fit for your research. If you are not sure, read the appropriate sections before making your selection.

☒ Life sciences ☐ Behavioural & social sciences ☐ Ecological, evolutionary & environmental sciences

For a reference copy of the document with all sections, see nature.com/documents/nr-reporting-summary-flat.pdf

Life sciences study design

All studies must disclose on these points even when the disclosure is negative.

Sample size	N/A
Data exclusions	N/A
Replication	See the Statistics and Reproducibility Section of the manuscript for detailed information on how often each experiment was replicated.<br><br>In the case of protein preparations and the corresponding assays, biological replicates are defined as independent protein preparation which were obtained on independent days from independent protein producing cultures; technical replicates refer to repetition of similar experiments on independent days with protein preparations obtained from the same purification with storage at cryonic conditions.<br><br>For analysis of metabolite production by various mutants, biological replicates are defined as culture extract that were obtained from independent bacterial cultures grown from independent cryonic stocks; technical replicates refer to the analysis of the same culture extract in independent chromatographic runs.
Randomization	N/A
Blinding	N/A

Reporting for specific materials, systems and methods

We require information from authors about some types of materials, experimental systems and methods used in many studies. Here, indicate whether each material, system or method listed is relevant to your study. If you are not sure if a list item applies to your research, read the appropriate section before selecting a response.

Materials & experimental systems

n/a	Involved in the study	
☒	☐	Antibodies
☒	☐	Eukaryotic cell lines
☒	☐	Palaeontology and archaeology
☒	☐	Animals and other organisms
☒	☐	Human research participants
☒	☐	Clinical data
☒	☐	Dual use research of concern

Methods

n/a	Involved in the study	
☒	☐	ChIP-seq
☒	☐	Flow cytometry
☒	☐	MRI-based neuroimaging
